# Cell type–specific 3D-genome organization and transcription regulation in the brain

**DOI:** 10.1126/sciadv.adv2067

**Published:** 2025-02-26

**Authors:** Shiwei Liu, Cosmos Yuqi Wang, Pu Zheng, Bojing Blair Jia, Nathan R. Zemke, Peter Ren, Hannah L. Park, Bing Ren, Xiaowei Zhuang

**Affiliations:** ^1^ Howard Hughes Medical Institute, Department of Chemistry and Chemical Biology, Department of Physics, Harvard University, Cambridge, MA, USA.; ^2^ Department of Molecular and Cellular Biology, Harvard University, Cambridge, MA, USA.; ^3^ Bioinformatics and Systems Biology Graduate Program, Medical Scientist Training Program, University of California San Diego, La Jolla, CA, USA.; ^4^ Department of Cellular and Molecular Medicine and Center for Epigenomics, University of California, San Diego School of Medicine, La Jolla, CA, USA.; ^5^ Graduate Program in Biophysics, Harvard University, Cambridge, MA, USA.

## Abstract

3D organization of the genome plays a critical role in regulating gene expression. How 3D-genome organization differs among different cell types and relates to cell type–dependent transcriptional regulation remains unclear. Here, we used genome-scale DNA and RNA imaging to investigate 3D-genome organization in transcriptionally distinct cell types in the mouse cerebral cortex. We uncovered a wide spectrum of differences in the nuclear architecture and 3D-genome organization among different cell types, ranging from the size of the cell nucleus to higher-order chromosome structures and radial positioning of chromatin loci within the nucleus. These cell type–dependent variations in nuclear architecture and chromatin organization exhibit strong correlations with both the total transcriptional activity of the cell and transcriptional regulation of cell type–specific marker genes. Moreover, we found that the methylated DNA binding protein MeCP2 promotes active-inactive chromatin segregation and regulates transcription in a nuclear radial position–dependent manner that is highly correlated with its function in modulating active-inactive chromatin compartmentalization.

## Teaser

Genome-scale imaging reveals 3D-genome and transcription regulation across brain cell types and MeCP2 mutations.

## INTRODUCTION

The cell nucleus is the central hub for essential genomic functions, ranging from transcription and gene regulation to the replication of DNA. The morphology and molecular architecture of the cell nucleus are among the most distinct indicators of cell differentiation, aging, and disease progression (*1*, *2*). Imaging and biochemical studies have provided rich insights into the nuclear architecture and chromatin organization across scales (*3*–*10*). Recent development of high-throughput sequencing- and imaging-based assays for 3D-genome mapping has substantially advanced our understanding of the chromatin organization in the cell nucleus with a genome-wide view (*3*–*5*, *7*, *9*–*16*), revealing prominent chromatin structures such as A/B compartments (*17*), topologically associating domains (*18*–*20*), and chromatin loops (*21*). For example, both sequencing- and imaging-based methods have shown that chromatin in the interphase nucleus are segregated into compartments enriched for active and inactive chromatin marks (termed A and B compartments, respectively), with compartment-A chromatin being preferentially enriched in the nuclear interior and compartment-B chromatin enriched at the nuclear periphery and nucleoli (*17*, *22*–*28*).

Recent studies comparing different cell types in vitro and in the brain have begun to reveal cell type–dependent variations in 3D-genome organization and their relationship with transcription (*29*–*38*). However, what differences in chromatin organization are present among the large number of transcriptionally distinct cell types in complex tissues remains incompletely understood. What properties of the chromatin organization are related to transcription and what molecular mechanisms underlie these connections are also unclear.

3D organization of the genome is regulated by a variety of protein factors that establish, maintain, or modulate chromatin structures (*39*, *40*). However, for many transcription regulators, how they control or modulate chromatin organization is unclear. Many of these factors bind to gene regulatory elements marked by DNA or histone modifications. Among these proteins, the methyl-CpG binding protein 2 (MeCP2) binds to methylated DNA (*41*), as well as active chromatin, such as transcription start sites (TSSs) (*42*), and regulates transcription (*43*, *44*). Mutations in *MECP2* can cause Rett syndrome, a progressive neurological disorder (*45*–*48*), and other neurological disorders (*49*). Although *Mecp2* deletion or overexpression has been reported to change the size of the cell nucleus and gross nuclear architecture, as reflected by DAPI and histone mark stains (*50*–*53*), how MeCP2 affects higher-order chromosome structures and how the chromatin-organizing function of MeCP2 relates to transcriptional regulation remain unclear.

In this work, we studied the three-dimensional (3D)–genome organization and the role of MeCP2 in chromatin organization across different cell types in the brain using multiplexed error-robust fluorescence in situ hybridization (MERFISH), a genome-scale imaging method (*54*). We have previously demonstrated in situ cell-type identification in the brain using RNA-MERFISH (*55*–*57*) and the determination of 3D-genome organization in cultured cells using DNA-MERFISH (*25*). Here, we extended DNA-MERFISH to intact tissue samples and combined it with RNA-MERFISH to probe 3D-genome organization in ~110,000 cells of 21 major cell types in the cortex of the wild-type (WT) and *Mecp2* mutant mouse brains. Using this approach, we observed multiple levels of differences in the nuclear architecture and chromatin organization among different cell types that were related to transcriptional regulation. First, we showed that the cell-nucleus sizes varied substantially across different cell types, in a manner that was strongly correlated with the total transcriptional activity of the cells. Second, we observed different 1D-to-3D scaling (i.e., spatial distance versus genomic distance scaling) behaviors of chromatin in different cell types, and this scaling varied with the transcriptional activity in markedly different manners at different genomic length scales. Third, we observed both global and local cell type–dependent variations in higher-order chromosome structures. At the global level, we observed a counterbalance between two major chromosome structures, megadomains and A/B compartment, with the balance shifting from the former to the latter as the overall transcriptional activity increased from non-neuronal cells to neurons. Locally, the transcriptional activity of genes differentially expressed between different cell types correlated with the enrichment of compartment-A chromatin near these genes. In addition, we observed different nuclear radial positioning of genomic loci in different cell types with a stronger segregation of transcriptional active and inactive chromatin along the nuclear radial axis in non-neuronal cells than in neurons. Last, we found a genome-wide nuclear radial position dependence in the transcriptional regulation by MeCP2 and a role of MeCP2 in chromatin organization. *Mecp2* deletion caused down-regulation of transcription for genes at the nuclear interior and up-regulation of transcription for genes at the nuclear periphery, and this effect was substantially stronger in neurons than in non-neuronal cells. At the chromatin-organization level, we found that *Mecp2* deletion caused a weakening of A/B-compartment chromatin segregation, resulting in less active chromatin environment at the nuclear interior and more active chromatin environment at the nuclear periphery, providing a potential mechanism that could explain the nuclear radial position dependence in the transcriptional regulation by MeCP2. Overall, our data provide rich insights into how 3D chromatin organization varies in different cell types and relates to transcriptional activities in a cell type–specific manner in the brain.

## RESULTS

### Integrated RNA- and DNA-MERFISH for cell type–specific chromatin-organization mapping

We chose the mouse primary motor cortex (MOp) as a model system to study the cell type–specific chromatin organization and its relationship to transcriptional regulation. A cell-type atlas of the MOp has been generated recently by the BRAIN Initiative Cell Census Network (*58*). Specifically, single-cell/single-nucleus RNA sequencing (sc/snRNA-seq) and epigenomic sequencing studies have extensively characterized the cell-type taxonomy and the transcriptional regulatory landscape across different cell types in the MOp (*59*–*61*). In parallel, we have generated a spatially resolved cell atlas of the MOp by RNA-MERFISH, which describes transcriptionally distinct cell types and their spatial organization in this brain region (*62*).

Here, we sought to combine RNA-MERFISH and DNA-MERFISH for simultaneous determination of the cell-type identity and chromatin structures in individual cells, thereby characterizing cell type–specific chromatin organization. We introduced several modifications to our previous MERFISH protocols to facilitate the combination of RNA- and DNA-MERFISH in tissues. To preserve 3D chromatin structures in their native environment and facilitate DNA- and RNA-MERFISH in the same sample, we omitted the gel embedding–based tissue clearing (*63*) previously used in RNA-MERFISH characterizations of the brain, including the MOp (*62*). To compensate for the decrease in the signal-to-noise ratio resulted from omitting tissue clearing, we amplified the signal by using adaptor probes to link dye-labeled readout probes to the encoding probes targeting the RNA or DNA (*25*). This approach allows more dye-labeled readout probes to be bound to each encoding probe and hence increases signals from individual RNA molecules and genomic loci. In addition, we used photobleaching to reduce autofluorescence of the tissue slices (*56*). With these modifications, we performed RNA-MERFISH measurements on coronal mouse brain sections encompassing the MOp and its vicinity (fig. S1). Subsequently, we conducted DNA-MERFISH on the same tissue sections and registered the DNA and RNA images to obtain transcriptional profiles and 3D-genome organization in the same cells (fig. S1).

To systematically study the cell type–dependent chromatin organization, we designed encoding probe libraries to target RNAs and chromatin in the following manner. For RNA-MERFISH, we utilized the same MOp encoding-probe library as in our previous work, targeting 242 marker genes for cell-type identification (*62*). For DNA-MERFISH, we designed three different encoding-probe libraries respectively targeting three groups of genomic loci (1981 loci in total): (i) 988 loci evenly distributed across the mouse genome with ~2.5-Mb spacing; (ii) 28 loci centered around TSSs for representative marker genes for the major cell types in the MOp; (iii) 965 loci centered around candidate super-enhancers, genomic regions comprising multiple putative enhancers and implicated in cell-type and gene-expression specification (*64*, *65*), selected using the published single-nucleus Assay for Transposase-Accessible Chromatin with sequencing (snATAC-seq) data (*66*).

**Fig. 1.**
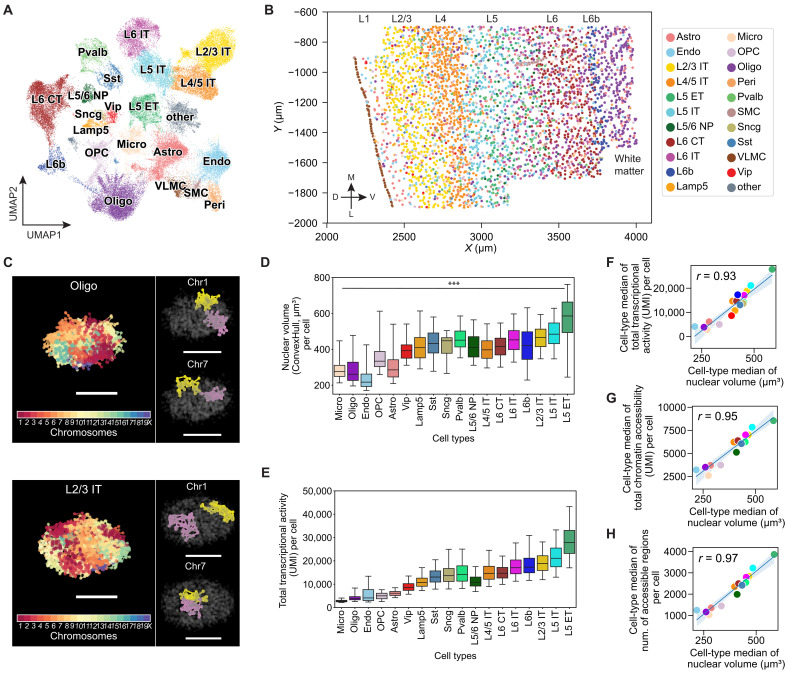
Integrated 3D-genome and transcriptome imaging revealed cell type–dependent variations of and correlation between cell-nucleus size and transcriptional activity. (**A**) Uniform manifold approximation and projection (UMAP) plot of MOp cell types determined by RNA-MERFISH. Cell-type identities are colored consistently across all panels. Micro, microglia; Oligo, oligodendrocytes; OPC, Oligodendrocyte progenitor cells; Astro, astrocytes; Endo, endothelial cells; Peri, Pericytes. Vip, Lamp5, Sst, Sncg and Pvalb: inhibitory neurons; L2/3 IT, L4/5 IT, L5 IT, L6 IT, L5 ET, L5/6 NP, L6 CT and L6b: excitatory neurons. (**B**) Spatial map of cell types in a coronal slice containing the MOp, with cortical layers (L1-L6b) indicated. D, dorsal; L, lateral; M, medial; V, ventral. (**C**) 3D renderings of imaged chromosome loci in an oligodendrocyte (top) and a L2/3 IT neuron (bottom) determined by DNA-MERFISH. Left: All loci colored by their chromosome identities. Right: homologs of Chr1 and Chr7 distinctively colored. Scale bars, 5 μm. (**D**) Boxplot of nuclear volume distributions across cells by cell type. Nuclear volume per cell was calculated by the convex hull volume of all chromosome loci. ***P ≤ 0.0001 for both one-way analysis of variance (ANOVA) across all cell types and Tukey’s post hoc multiple comparison test between L5 ET and Micro. (**E**) Boxplot of distributions of total transcriptional activity—approximated by the sum of unique molecular identifiers (UMIs) from snRNA-seq data (*59*)—across cells by cell type. (**F**) Scatterplot of cell-type medians of total transcriptional activity versus nuclear volume. (**G** and **H**) Scatterplots of cell-type medians of nuclear volume versus total chromatin accessibility (G) or versus number of accessible chromatin regions (H). Chromatin accessibility was calculated from snATAC-seq data (*59*). In (F) to (H), each dot represents a cell type, with Pearson correlation coefficients (*r*) indicated.

Using this design, we profiled transcription of the 242 genes in ~46,000 cells in the WT mouse brain and identified 21 cell types at the subclass level in the MOp and adjacent areas by de novo cell clustering (Fig. 1A). These include eight subclasses of excitatory neurons [layer 2/3 (L2/3) intratelencephalic (IT), L4/5 IT, L5 IT, and L6 IT, L5 extratelencephalic (ET), L5/6 near projection (NP), L6 cortical-thalamic projection (CT), and L6b neurons], five subclasses of inhibitory neurons annotated by their canonical marker genes (*Pvalb*, *Sst*, *Lamp5*, *Vip*, and *Sncg*) and eight subclasses of non-neuronal cells [astrocytes, oligodendrocytes, oligodendrocyte progenitor cells (OPCs), microglia, endothelial cells, pericytes, vascular leptomeningeal cells (VLMCs), and smooth muscle cells (SMCs)] (Fig. 1, A and B). The overall expression profiles of MOp cells obtained using our modified RNA-MERFISH protocol agreed well with both our previous results from tissue-cleared RNA-MERFISH (fig. S2A) (*62*) and bulk RNA-seq data (fig. S2B). Our data also showed high reproducibility between experimental replicates (fig. S2C). The cell types identified using this modified RNA-MERFISH protocol showed a one-to-one correspondence with the cell types determined from tissue-cleared RNA-MERFISH data (*62*), except for the low abundance perivascular macrophages (PVMs), which were not identified presumably due to fewer cells measured here (fig. S2, D and E).

Next, we determined the 3D-genome organization for each identified cell type. We used 99-bit and 95-bit error-correcting Hamming codes to encode the 988 genome-wide loci and 965 super-enhancer loci, respectively, and imaged these 1953 loci using two back-to-back DNA-MERFISH measurements. In addition, we imaged the 28 marker gene TSS loci using sequential multicolor DNA-FISH after DNA-MERFISH measurements, all on the same samples. We analyzed the DNA-MERFISH images by adapting the recently reported spatial genome aligner algorithm (*67*), which allowed us to determine the copy number of chromosomes and trace chromatin loci within each chromosome (Fig. 1C). We found that the median pairwise spatial distance between our imaged loci showed a high correlation with the pairwise contact frequency recently measured by single-nucleus methylome (snm) and chromatin conformation capture (3C) sequencing (snm3C-seq) (fig. S3, A and B) (*35*) and high reproducibility between experimental replicates (fig. S3, C and D), demonstrating that the 3D chromosome structures were well preserved in their native tissue contexts in our integrated RNA- and DNA-MERFISH measurements.

### Cell type–dependent variations in nucleus and chromosome-territory sizes and their relationship with transcriptional activity

We characterized the size of the cell nucleus in each cell type and observed substantially different cell-nucleus volume for different cell types, with non-neuronal cells, inhibitory neurons, and excitatory neurons exhibiting increasingly larger nuclear volume (*P* < 0.0001; threefold difference between cell types with the smallest and largest nuclear volume; Fig. 1D and fig. S4). Within major cell classes, different subclasses of cells also showed different nuclear volumes. For example, among excitatory neurons, L5 ET and L4/5 IT showed the largest and smallest nuclear volume, respectively (~1.5-fold difference, *P* < 0.0001; Fig. 1D). Even among IT neurons, cells in different cortical layers showed different nuclear sizes (*P* < 0.0001; Fig. 1D). These results expanded upon previous knowledge (*36*, *68*, *69*) and provided a higher-granularity characterization of cell-nucleus sizes across different cell types in the mouse cortex.

Next, we investigated the relationship between the cell-nucleus size and transcription by using a published snRNA-seq dataset (*59*) to obtain near genome-wide RNA expression levels in different cell types in the MOp. Notably, we observed a strong correlation between the cell-nucleus size and total transcriptional activity of the cell across different cell types using total nuclear RNA count as an approximation for total transcriptional activity (Fig. 1, D to F). The cell type with the largest nuclear volume, L5 ET, showed a ~10-fold higher total transcript counts than that observed in the cell types with the smallest nuclear sizes, i.e., microglia, oligodendrocytes, and endothelial cells (Fig. 1, D and E). Even among neurons, L5 ET showed a ~3-fold greater total transcript counts compared to the *Vip*-positive inhibitory neurons, the neuronal cell type with the smallest nuclear volume (Fig. 1, D and E). A similar trend in transcriptional activity changes across cell types was also observed in the human cortex (fig. S5) (*56*, *70*). We also observed a strong correlation between the cell-nucleus size and chromatin accessibility across different cell types (Fig. 1, G and H) using a published snATAC-seq dataset (*59*) to assess chromatin accessibility. Moreover, we found that the nuclear volume was also positively correlated with the total transcript counts of the 242 genes measured by RNA-MERFISH at single-cell level (fig. S6), suggesting that the cell-nucleus size is correlated with the total transcriptional activity not only across cell types but also likely across individual cells within the same cell type.

Next, we investigated whether transcriptional activity was also correlated with the physical sizes of chromosome territories. Using the radius of gyration of the imaged chromatin loci within individual chromosomes to approximate the chromosome-territory size, we observed that cell types with larger nuclear volume generally had larger chromosome-territory sizes, except that most inhibitory neurons exhibited disproportionately larger chromosome-territory sizes compared to excitatory neurons with the same nucleus size (fig. S7, A and B). Consistent with this observation, inhibitory neurons displayed more intermixing of chromosome territories than excitatory neurons (fig. S7C). We also observed a positive correlation between the overall transcriptional activity of the chromosomes, estimated from the snRNA-seq data, and the chromosome-territory sizes across different cell types (fig. S7D). This correlation was weaker compared to the correlation with the nuclear volume (Fig. 1F), possibly due to the stronger influence that each chromosome received from other chromosomes invading its territory in inhibitory neurons. Consistent with this notion, excitatory neurons, which exhibited stronger segregation between chromosome territories (fig. S7C), also showed higher correlation between transcriptional activity and chromosome-territory sizes (fig. S7E).

### Cell type–dependent variations in chromatin scaling and their relationship with transcriptional activity

How the distances between genomic loci in the 3D space scale with their 1D distances along the genomic coordinate provides insights into how chromatin, as a polymer-like structure, is packaged in space. For example, Hi-C measurements of how chromatin contact frequency changes with genomic separation at the megabase scale suggest that chromatin packaging in cultured cells approximately follows the fractal globule–like scaling, which reflects confinement of an unknotted polymer to a small volume (*17*). Super-resolution imaging of cultured cells has revealed distinct 1D-to-3D scaling properties for chromatin in different epigenetic states (*71*). Some cell type–dependent variations in the spatial-vs-genomic distance curves have also been observed, but without quantitative analysis (*36*). A systematic understanding of how the scaling properties of chromatin differ among different brain cell types and how chromatin scaling is related to transcriptional activity is still missing.

**Fig. 2.**
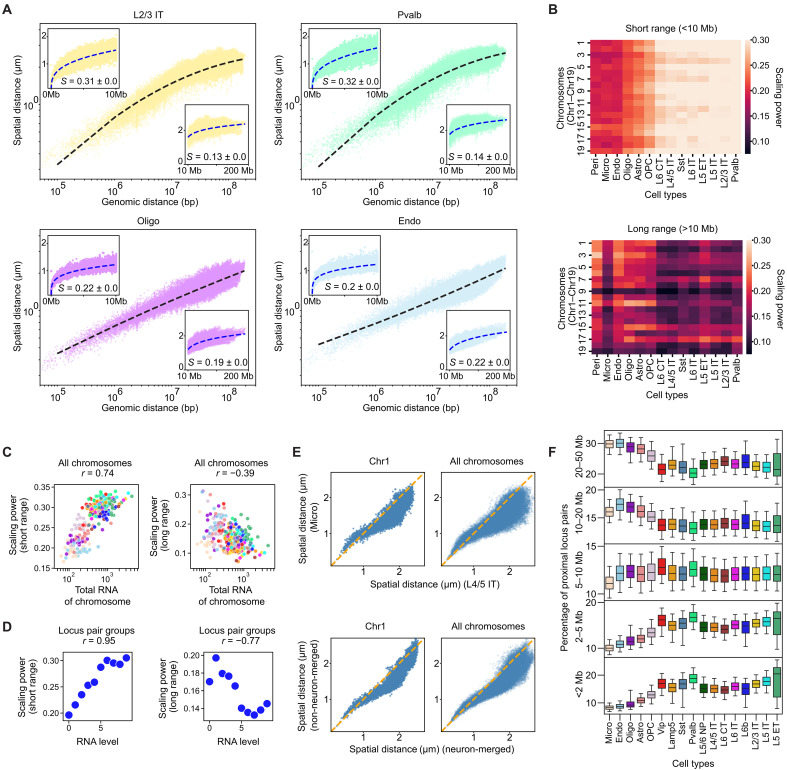
Cell type–dependent variations in chromatin scaling and their relationship to transcriptional activity. (**A**) Scatterplots of spatial versus genomic distances on log-log scale for cis-chromosomal locus pairs from all chromosomes for various cell types. Insets: data on linear scale for short (<10 Mb, top left) and long (>10 Mb, bottom right) genomic distance ranges. Dashed lines in main plots and insets indicate a second-order polynomial fit and a power law fit, respectively. S, scaling power exponents. (**B**) Heatmaps of scaling power exponents by chromosomes (Chr1 to Chr19) and cell types at short (<10 Mb, top) and long (>10 Mb, bottom) genomic distance ranges. (**C**) Scatterplots of scaling power exponents versus total transcriptional activity of chromosomes [transcriptional activity derived from snRNA-seq data (*59*)] at short (<10 Mb, left) and long (>10 Mb, right) genomic distance ranges. Each dot represents a chromosome in a cell type, color-coded by the cell type as in Fig. 1A. (**D**) Scatterplots of scaling power exponents versus transcriptional activity for binned locus-pair groups at short (<10 Mb, left) and long (>10 Mb, right) genomic distance ranges. Each dot represents a group of genomic locus pairs, grouped by their transcriptional activity across all chromosomes. In (C) and (D), Spearman correlation coefficients (*r*) are indicated. (**E**) Scatterplots of cell-type median spatial distances for cis-chromosomal locus pairs between microglia and L4/5 IT neurons (top) and between non-neuronal cells and neurons (bottom). Left and right: Locus pairs from Chr1 and all chromosomes, respectively. Dashed lines indicate equality. (**F**) Boxplots of distributions of the percent of spatially proximal locus pairs (within 750 nm) in specified genomic distance ranges (over all detected proximal pairs) by cell type. A small fraction of spatially proximal locus pairs in the genomic distance range of >50 Mb are not shown.

We investigated how the 3D spatial distances between imaged loci scaled with their 1D genomic distances in the different cell types observed in the MOp. We observed cell type–dependent variations in the spatial versus genomic distance scaling that differed at different genomic length scales. This scaling followed a simple power law with a single power exponent for most non-neuronal cell types, as reflected by a linear relationship in the log-log plots (Fig. 2A and fig. S8). In contrast, the spatial versus genomic distance curves showed a clear bending at ~10-Mb genomic distance for neurons (Fig. 2A and fig. S8). To further quantify the scaling behavior, we determined the exponents of the power-law fitting function for the spatial versus genomic distance curves in two genomic distance ranges (<10 Mb and >10 Mb). Consistent with the visual impression, the scaling power exponents were essentially identical in the two ranges for most non-neuronal cell types but substantially different in these two ranges for the neuron cell types. At the short-range (<10 Mb), the scaling power exponents in neurons were larger than those in non-neuronal cells, whereas at the long range (>10 Mb), the scaling power exponents in neurons were smaller than or comparable to those in non-neuronal cells (Fig. 2, A and B, and fig. S8).

Next, we asked whether our observed cell type–dependent variations in chromatin scaling are related to transcriptional activity. To this end, we performed two analyses. First, we quantified both the scaling power exponents and the total transcriptional activity for individual chromosomes (Chr1 to Chr19) within individual cell types and found that the scaling power was positively correlated with the transcriptional activity at the short genomic distance range (<10 Mb) but negatively correlated with the transcriptional activity at the long genomic distance range (>10 Mb; Fig. 2C). In the second analysis, we used transcriptional activity of individual chromatin loci to group loci from all chromosomes across all cells into 10 bins and determined the scaling power for locus pairs within each bin. A similar relationship between the scaling power and transcriptional activity was observed with this latter analysis: The scaling power increased with the transcriptional activity at the short genomic distance range (<10 Mb) but largely decreased with transcriptional activity at the long genomic distance range (>10 Mb; Fig. 2D). Together, these results suggest a previously unidentified relationship between chromatin scaling and transcription.

At the smallest length scale probed in this work (~1 to 2 Mb), the spatial distances between locus pairs were smaller in neurons than in non-neuronal cells (Fig. 2E, above the *y* = *x* dashed line), although neurons had larger cell-nucleus and chromosome-territory sizes than non-neuronal cells (Fig. 1D and fig. S7A). Because of the greater scaling power of spatial-vs-genomic distance relationship in neurons than in non-neuronal cells at <10 Mb (Fig. 2B), the difference in spatial distances between neurons and non-neuronal cells observed at the smallest genomic distances diminished and then reversed sign as the distance increased, such that the spatial distances became greater in neurons than in non-neuronal cells at relatively large distances (Fig. 2E). The difference eventually diminished again (Fig. 2E) due to the smaller scaling power in neurons as compared to non-neuronal cells at >10 Mb (Fig. 2B). As a corollary, we observed different trends for the cell-type dependence in the spatial proximity frequencies between locus pairs at different length scales: Among the locus pairs that were proximal with each other (<750-nm cutoff distance), the percentage of them that had relatively small genomic distances tended to be greater in neurons than in non-neuronal cells, whereas the percentage that had relatively large genomic distances tended to be smaller in neurons than in non-neuronal cells (Fig. 2F), consistent with sequencing-based studies reporting that non-neuronal cells and neurons exhibit distinct preferences for cis-chromosomal contacts at different genomic distances (*32*–*34*, *37*).

### Cell type–dependent variations in higher-order chromosome structures and their relationship with transcriptional activity

**Fig. 3.**
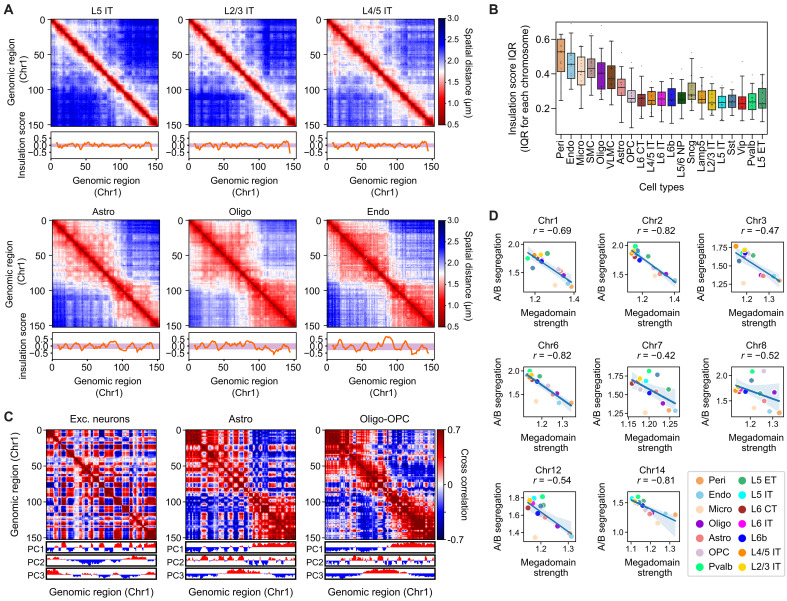
Cell type–dependent variations in higher-order chromosome structures and their relationship to transcriptional activity. (**A**) Median pairwise spatial distance matrices for Chr1 in various cell types, with normalized insulation scores (Additional Materials and Methods in Supplementary Materials) along the genomic coordinate shown below. Genomic regions 0 to 152 corresponding to 3.7 to 193.7 Mb in Chr1. Purple shades represent the interquartile ranges (IQRs; 25th to 75th percentiles) of normalized insulation scores. (**B**) Boxplots of the distributions of normalized insulation score IQR across all chromosomes by cell type. (**C**) Cross-correlation matrices of Chr1 in several major cell types. Each element in the matrix represents the Pearson correlation coefficient between the indicated locus pair in terms of their normalized proximity frequency with all other loci from Chr1 (Additional Materials and Methods in Supplementary Materials). The three rows below the matrices indicate the first three PC values (positive values: red; negative values: blue). (**D**) Scatterplots of A/B segregation scores versus megadomain strengths for indicated chromosomes across different cell types (Additional Materials and Methods in Supplementary Materials). Only chromosomes that formed near-bipartite megadomains (i.e., two major megadomains) in non-neuronal cell types are shown, and we assigned a fixed megadomain boundary across cell types using the boundary observed in endothelial cells or pericytes. Each dot represents a cell type. Spearman correlation coefficients (*r*) are indicated in each plot.

The DNA-MERFISH data further allowed us to determine the higher-order chromosome structures. To this end, we computed the median pairwise spatial distance matrices for different chromosomes in different cell types. We observed pronounced global structural differences between different cell types. In particular, when comparing non-neuronal cells and neurons, we observed that chromosomes in non-neuronal cells had a higher tendency to form large-size domains (Fig. 3A and fig. S9) that resemble the “megadomains” in the transcriptionally inactive X chromosomes (*21*, *23*, *72*–*74*). We quantified the prevalence of these megadomain-like structures using the insulation score calculation (Materials and Methods and Additional Materials and Methods in Supplementary Materials) and observed more prominent insulation-score peaks, corresponding to the boundaries of megadomain structures, in chromosomes inside non-neuronal cells as compared to neurons (Fig. 3, A and B, and fig. S9). Because non-neuronal cells generally have lower transcriptional activity than neurons (Fig. 1, E and F), we speculate that inactive chromatin may promote the formation of megadomain structures in non-neuronal cells in a manner similar to how it occurs in inactive X chromosomes (*73*, *74*).

We also observed notable differences in the degree of A/B compartment formation across different cell types, particularly between neurons and non-neuronal cells. We applied standard principal component (PC) analysis to determine A/B compartments (*17*) in all major cell types. For each cell type, we calculated the normalized proximity frequency matrix, computed the cross-correlation matrix from this normalized proximity frequency matrix, performed PC analysis, and then selected the PC that showed the highest correlation with the CpG density to determine A/B compartments in that cell type (Materials and Methods and Additional Materials and Methods in Supplementary Materials). We found that the largest PC (PC1) did not always correspond to the A/B compartments in all cell types (Fig. 3C). For example, in excitatory neurons, the correlation matrix of Chr1 exhibited a pronounced plaid pattern, indicative of A/B compartment arrangements (Fig. 3C). Among all PCs, PC1 of the correlation matrix showed the highest correlation with the CpG density and chromatin accessibility measured by snATAC-seq (*59*) (fig. S10), representing A/B compartmentalization. In contrast, in oligodendrocytes, PC1 primarily corresponded to the megadomain structures described above, whereas PC2 showed the highest correlation with the CpG density and chromatin accessibility (Fig. 3C and fig. S10). Astrocytes displayed an intermediate behavior, where megadomain structures and A/B compartments were similarly prominent and both PC1 and PC2 showed similar correlation coefficients with the CpG density and chromatin accessibility (Fig. 3C and fig. S10).

After A/B compartment assignment for different cell types, we computed an A/B compartment segregation score for each chromosome in each cell type, defined as the ratio of the median of normalized spatial distance (normalized to control for genomic distance effect; Materials and Methods and Additional Materials and Methods in Supplementary Materials) of A-B locus pairs to that of the A-A and B-B locus pairs (fig. S11, A and B). This quantification showed that neurons in general tended to show stronger A/B compartment segregation than non-neuronal cells (fig. S11C). Given that neurons had higher overall transcriptional activities than non-neuronal cells (Fig. 1, E and F), the observation that neurons showed more prominent A/B-compartment segregation suggests a possibility that a stronger degree of A/B compartmentalization may cause higher transcriptional activity or vice versa, for example, through the formation of transcriptional condensates (*6*, *7*, *9*).

To quantitatively characterize the relationship between the degree of A/B-compartment segregation and the megadomain strength across different cell types, we used the ratio of median intermegadomain spatial distances over median intramegadomain spatial distances to define the megadomain strength. For the chromosomes that formed megadomains, we observed a negative correlation between the A/B compartment segregation score and the megadomain strength across cell types (Fig. 3D), suggesting that these two types of chromosome structures may counteract each other.

### Relationship between local A/B compartmental environment and transcriptional activity

**Fig. 4.**
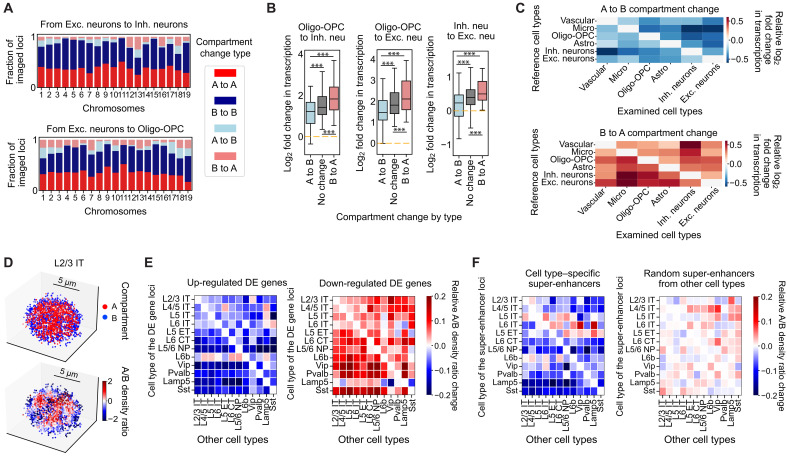
Relationship between A/B compartment organization and transcriptional activity. (**A**) Barplots of fractions of imaged loci showing A/B compartment conservations and changes from excitatory (Exc.) to inhibitory (Inh.) neurons (top), or from Exc. neurons to oligodendrocytes and OPCs (bottom). (**B**) Boxplots of transcription changes of individual loci grouped by A/B compartment conservations or changes between cell types. ***P ≤ 0.001 for one-way ANOVA with Tukey’s post hoc multiple comparison test. (**C**) Heatmaps of mean relative transcription changes for loci changed from A to B (top) or B to A (bottom) compartments between examined and reference cell types. The relative transcription changes were calculated by normalizing to transcription changes of loci with conserved compartment identities. Vascular cells include endothelial cells and pericytes. (**D**) Single-cell 3D renderings of compartment-A and compartment-B loci (top) and their local A/B density ratio (bottom) (Additional Materials and Methods in Supplementary Materials). Scale bars, 5 μm. (**E**) Local A/B density ratio changes for cell type–specific differentially expressed (DE) gene loci between reference cell types (where DE gene loci were identified; see Additional Materials and Methods in Supplementary Materials) and the other cell types. For each cell type, an imaged locus is considered a DE gene locus if there is a DE gene within 100 kb of this locus. Left: Each heatmap pixel represents the median value of fractional changes in the median A/B density ratio of all up-regulated DE gene loci for each cell type (*x* axis) over the reference cell type (*y* axis). Right: Similar to left, but for down-regulated DE gene loci. (**F**) Similar to (E), but for local A/B density ratio changes for cell type–specific super-enhancer loci (left) or a random set of other super-enhancer loci selected regardless of cell-type identity (right). Cell type–specific super-enhancers were selected by snATAC-seq data (*66*).

We observed not only different degrees of A/B compartmentalization in different cell types but also changes in compartmental identity for some genomic loci between cell types. For example, between neurons and non-neuronal cells (e.g., excitatory neurons versus oligodendrocytes), as well as between excitatory and inhibitory neurons, 10 to 30% of genomic loci showed different compartment identities (Fig. 4A). From non-neuronal cells to neurons, or from inhibitory to excitatory neurons, the transcriptional activity of genomic loci tended to increase in general, regardless of how the compartment identity of genomic loci was different between the compared cell type pairs (Fig. 4B). However, the genomic loci that changed from B to A compartment from one cell type to another in general showed a greater increase in transcriptional activity than those that did not change or change from A to B compartments (Fig. 4B). After normalization of cell type–dependent differences in the total transcriptional activity, genomic loci that changed from B to A compartment generally showed increased transcription, and genomic loci that changed from A to B showed decreased transcription (Fig. 4C).

Next, we explored the relationship between transcriptional activity of specific genes and their local A/B chromatin environment using a local A/B density ratio [Fig. 4D, defined as the ratio between local spatial densities of trans-chromosomal compartment-A and compartment-B chromatin loci for each gene of interest in each cell (*25*)]. The median of this ratio among all cells in any given cell type provides a quantitative proxy of the local A/B chromatin environment for each genomic locus in each cell type. We then investigated how this local A/B chromatin environment is related to the differential expression of genes across different cell types. To this end, we first identified the top differentially expressed (DE) genes (~200 up-regulated genes and ~200 down-regulated genes) in each neuronal cell type versus all other neuronal cell types using snRNA-seq data (*59*). Among these top DE genes, we selected those genes that were within 100 kb of our imaged genomic loci and then calculated the changes in their median local A/B density ratios between the cell types where the DE genes were identified versus other cell types. We found that genes that were up-regulated in a cell type tended to have higher local A/B density ratios in that cell type as compared to other cell types (Fig. 4E, left). Likewise, genes that were down-regulated tended to have lower local A/B density ratios in that cell type (Fig. 4E, right). We note that because of the normalization of total transcript count for each cell typically done in the DE gene analysis of sc/snRNA-seq data, up-regulated or down-regulated genes between cell types refer to those genes that are more up-regulated or down-regulated as compared to the overall behavior of all genes. Likewise, we also normalized the local A/B density ratio to remove the overall difference in the mean A/B density ratio (averaged across all genomic loci) between cell types. Hence, genomic loci exhibiting an increase or decrease in the local A/B density ratio between cell types also reflect a change of local A/B enrichment relative to the overall behavior of all genomic loci. Thus, our data suggest that up-regulated genes in a cell type tend to show higher local enrichment of compartment-A versus compartment-B chromatin in this cell type than in other cell types, in a relative sense compared to the overall behavior of all genomic loci.

We also examined the relationship between the local A/B chromatin environment and the activity of super-enhancers, which contribute to cell type–specific activation of gene expression (*64*, *65*). Similar to the trend observed for the DE genes, the cell type–specific super-enhancers also tended to have higher local A/B density ratios in their respective cell type as compared to other cell types (Fig. 4F, left). In contrast, this trend was not observed in a randomization control where the super-enhancers were randomly selected regardless of cell-type identity (Fig. 4F, right). Together, these results suggest that the local A/B-compartment chromatin environment may play a role in regulating cell type–specific gene expression, not only for genes themselves but also for gene regulatory elements. Alternatively, it is also possible that the activity of these genes and gene-regulatory elements caused a local enrichment of other active chromatin.

For some genomic loci that showed relatively strong expression in a cell type that has a low overall transcription activity, we also observed a “looping-out” phenomenon that moved the loci out of the repressive environment. For example, when comparing the median pairwise distance matrices of Chr16 between astrocytes and L4/5 IT neurons, we observed a genomic region where the physical distances between this region and the down-stream regions were larger in astrocytes as compared to those observed in L4/5 IT neurons, although Chr16 was overall more compact in astrocytes (fig. S12, A and B, indicated by black boxes). The “looping-out” configuration of this region could also be directly visualized from individual chromatin traces at the single-cell level (fig. S12, C and D). This chromatin region also showed notably higher transcriptional activity in astrocytes than in L4/5 IT neurons, although the chromosome-wide transcription activity of Chr16 was overall lower in astrocytes (fig. S12A). Mechanistically, it is possible that the increased physical separation between this region and the other repressive regions in the chromosome helped increase its transcriptional activity or vice versa.

### Cell type–dependent variations in nuclear radial positioning of active and inactive chromatin

We calculated the normalized radial positions of our imaged loci (normalized by the radius of the nucleus in the same direction) and observed distinct radial positioning for different genomic loci in different cell types (fig. S13A). In general, neurons and non-neuronal cells showed lower similarity in their nuclear radial positioning profiles of genomic loci than the similarities observed among subtypes of neurons or among non-neuronal cell types (fig. S13B).

**Fig. 5.**
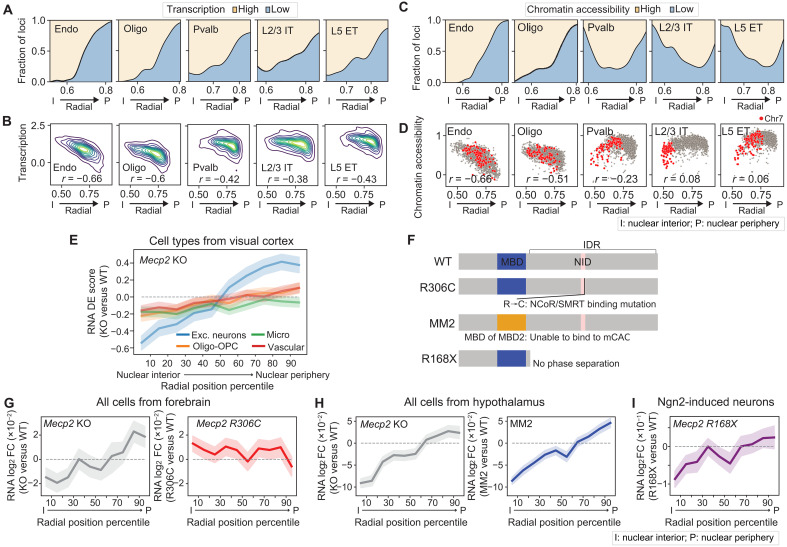
Nuclear radial positioning of active and inactive chromatin and radial position–dependent transcriptional regulation by MeCP2. (**A**) Fractions of genomic loci with high and low transcriptional activity as a function of median normalized nuclear radial position. The fractions of high- and low-expression loci [top 25th and bottom 25th percentile, respectively, calculated from snRNA-seq data (*59*)] over the total counts of high- and low-expression loci were plotted. (**B**) Kernel density estimation of distributions of cell-type median transcriptional activity versus normalized nuclear radial positions across chromatin loci. (**C**) Similar to (A) but for chromatin accessibility, calculated from snATAC-seq data (*59*). (**D**) Scatterplots of cell-type median chromatin accessibility versus normalized nuclear radial positions across chromatin loci. Gray: All chromosomes. Red: Chr7. Spearman correlation coefficients (*r*) indicated in (B) and (D). (**E**) Transcriptional changes upon *Mecp2* deletion versus cell-type median normalized nuclear radial positioning, grouped into 10 equal bins. DE scores [*t* statistics comparing *Mecp2* KO and WT cells from scRNA-seq (*83*)] are plotted with mean (line) and 95% confidence interval (shade). (**F**) Schematics of *Mecp2* mutations analyzed in (G) to (I). MBD: methyl-CpG (mCG) binding domain (blue); NID: NCoR/SMRT-interacting domain (pink); IDR: intrinsically disordered region. R306C (*80*, *85*): disrupting NCoR/SMRT interaction; MM2 (*86*): replacing the MBD of MeCP2 with the MBD of the MBD2 protein, which abolishes binding to methylated CAC (mCAC) but not mCG; R168X (*50*): truncating IDR. (**G**) Similar to (E), but for transcriptional changes [log_2_ fold change (FC) of RNA counts] upon *Mecp2* KO (left) or R306C mutation (right) measured in mouse forebrain (*85*). (**H**) Similar to (G), but for transcriptional changes upon *Mecp2* KO (left) or upon MM2 transgene introduction (right) in mouse hypothalamus (*86*). (**I**) Similar to (G), but for transcriptional changes upon R168X mutation in Ngn2-induced cultured neurons (*50*).

We observed different degrees of correlation between the transcriptional activity of genomic loci and their nuclear radial positions for different cell types. In non-neuronal cells, the transcriptional activity of genomic loci was strongly correlated with their nuclear radial positions, with loci positioned closer to the nuclear periphery exhibiting lower transcriptional activity (Fig. 5, A and B). This correlation was weaker in neurons, accompanied by relatively increased transcriptional activity for genomic loci near the nuclear periphery (Fig. 5, A and B). This difference between neurons and non-neuronal cells was even more pronounced for the correlation between chromatin accessibility and nuclear radial positioning (Fig. 5, C and D). Thus, our results showed that active and inactive chromatin tend to be better segregated along the nuclear radial axis in non-neuronal cells than in neurons.

Examination of the distribution of the nuclear radial position of genomic loci across individual cells showed that this distribution gradually shifted toward the nuclear periphery as the transcriptional activity of the genomic loci decreased (fig. S14). Chromatin loci located at the nuclear periphery tend to have smaller cell-to-cell variability in their nuclear radial positioning than those located near the nuclear center (fig. S15), suggesting the presence of a mechanism to stabilize the chromatin positioning at the nuclear periphery, possibly due to interaction with the nuclear lamina.

We also observed different degrees of correlation between transcriptional activity and nuclear radial positioning as well as between chromatin accessibility and nuclear radial positioning, for different chromosomes (fig. S16, A and B). For example, for Chr7, while the correlation between nuclear radial positioning and chromatin accessibility was negative for non-neuronal cells, it was positive for neurons (fig. S16B). Along the same line, H3K27ac and H3K9me3, histone modifications that mark active and inactive chromatin, respectively (*75*), also showed positive and negative correlations, respectively, with the nuclear radial positions in most neuronal cell types for Chr7 (fig. S16C) (*76*). Therefore, compared to non-neuronal cells, neurons have more active chromatin in the nuclear periphery and more inactive chromatin near the nuclear interior, partly due to the inverted radial organization of a subset of chromosomes.

Along with the increased transcriptional activity of genes near the nuclear periphery in neurons, we observed that the majority of the transcriptionally active genomic loci in the nuclear periphery of neurons contained actively expressed long genes (>300 kb) (fig. S17A). Many of these long genes are associated with Gene Ontology (GO) terms that are related to synapse functions (fig. S17B). In the nuclear interior, although long genes were not preferentially activated over short genes in neurons, there were still more transcriptionally active loci containing actively expressed long genes in neurons as compared to non-neuronal cells (fig. S17A).

### Radial-position-dependent transcriptional regulation by MeCP2

To further characterize the radial organization of chromatin in the cell nucleus of neurons, we compared the patterns of several active and repressive histone marks (*75*) and chromatin-binding proteins across all imaged loci (fig. S18). Among these factors that we examined, the binding of MeCP2 (*77*) consistently showed a high correlation with the nuclear radial positioning of chromatin in neurons, with a stronger tendency of MeCP2 binding to chromatin loci in the nuclear interior (fig. S18).

MeCP2 plays important roles in regulating gene transcription (*78*–*84*), and the deletion of the *Mecp2* gene has been shown to alter the expression level of thousands of genes, increasing transcription of a subset of these genes and reducing transcription of the others (*79*, *81*, *83*). To further characterize the effect of MeCP2 on transcriptional regulation, we first analyzed published scRNA-seq data from the cortex of WT (*Mecp2^+/y^*) and *Mecp2* knockout (KO, *Mecp2^−/y^*) male mice (*83*). As is routinely done in the DE gene analysis of scRNA-seq data, we determined differential expression between WT and KO samples with total transcript counts normalized per cell and thus genes identified as up-regulated or down-regulated upon *Mecp2* deletion refer to those genes that were up-regulated or down-regulated in a relative sense as compared to the average behavior of all genes. We mapped the genes in the scRNA-seq data to the nuclear radial positions of the closest genomic loci that we imaged. We focused on four major cell types from this dataset: excitatory neurons, oligodendrocytes and OPCs, microglia, and vascular cells (endothelial cells and pericytes) because the numbers of other cell types (inhibitory neurons and astrocytes) measured in this dataset are relatively low. Notably, we found that excitatory neurons showed a strong nuclear radial position dependence of transcriptional regulation by MeCP2: Transcription of genes at the nuclear interior was down-regulated, and genes at the nuclear periphery were up-regulated upon *Mecp2* deletion (Fig. 5E and fig. S19). Compared to excitatory neurons, the degree of transcriptional up- or down-regulation by MeCP2 was much weaker in non-neuronal cells (Fig. 5E and fig. S19).

To gain more insights into the potential mechanisms underlying the radial position–dependent effect of MeCP2 on transcription, we further examined the radial position–dependent effects of various *Mecp2* mutations (Fig. 5F) by analyzing bulk RNA-seq data. As described above, we mapped the genes in published bulk RNA-seq data of *Mecp2* KO and three *Mecp2* mutations or transgenes (R306C, MM2, and R168X) (*50*, *85*, *86*) to the nuclear radial positions of the closest genomic loci that we imaged. We observed a similar radial position–dependent effect of transcriptional activity change upon *Mecp2* deletion in two bulk RNA sequencing datasets of the mouse brain [Fig. 5, G (left) and H (left)]. Next, we examined the effect of the R306C mutation, which disrupts MeCP2 binding to the transcriptional co-repressor NCoR/SMRT (nuclear receptor corepressor/silencing mediator of retinoic acid and thyroid hormone receptor) complex (Fig. 5F) (*80*, *85*). In contrast to *Mecp2* deletion, the R306C mutation caused a largely radial position–independent increase in transcriptional activity (Fig. 5G, right), indicating that the radial position dependence of transcriptional regulation by MeCP2 does not require interaction with the NCoR/SMRT complex.

MeCP2 can bind to both methylated CG and methylated CA, with stronger affinity to methylated CAC (mCAC) than other forms of mCAH trinucleotides (*82*, *87*). To understand which binding mode of MeCP2 is important for its radial position–dependent transcriptional regulation effect, we analyzed bulk RNA-seq data on the knock-in mice *Mecp2-MBD2* (MM2), which specifically abolishes MeCP2 binding to mCAC but not to mCG by replacing the methylated DNA binding domain (MBD) of *Mecp2* with that of the MBD2 protein (Fig. 5F) (*86*)]. Our analysis results showed that the MM2 transgene recapitulated the radial position–dependent effect of *Mecp2* deletion on transcription (Fig. 5H, right).

We also investigated the effect of the *Mecp2* truncation R168X, which lacks the intrinsically disordered region of MeCP2 required for liquid-liquid phase separation (Fig. 5F) (*50*). Our analysis of the RNA-seq data in Ngn2-induced neurons expressing R168X (*50*) showed that this mutation also exhibited a qualitatively similar radial position dependence on transcription as seen for *Mecp2* deletion (Fig. 5I). However, without *Mecp2* deletion data of Ngn2-induced neurons in the same dataset, it is unknown whether the magnitude of the R168X effect was also quantitatively similar to that of *Mecp2* deletion.

Together, our analysis results suggest that MeCP2 regulates gene expression in a radial position–dependent manner, potentially through its binding to mCAC and in a way related to the formation of phase-separated condensates. As a cautionary note, R168X truncates a large portion of MeCP2 and hence may also impair other functions of MeCP2 unrelated to condensate formation. We also note that the ability of MeCP2 to recruit the NCoR/SMRT corepressor complex has been previously shown to contribute to MeCP2’s role in transcriptional repression (*80*, *85*), and our data support the involvement of this mechanism in transcriptional repression by MeCP2 but showed that this effect occurs in a manner that is largely independent of the nuclear radial position. It is also interesting to note that the R306C mutation confers milder symptoms in human patients as compared to the R168X mutation or other *MECP2* large DNA deletions (*88*). Hence, the radial positioning–dependent transcriptional regulation by MeCP2 may be particularly important for the clinical severity in Rett syndrome.

### Effect of MeCP2 on chromatin organization and its relationship to transcriptional regulation

**Fig. 6.**
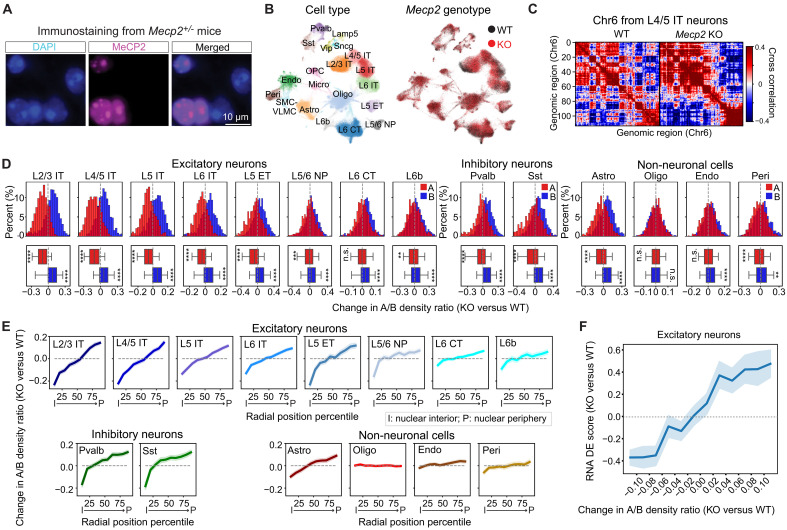
MeCP2 regulates local chromatin organization in a nuclear radial position and cell type–dependent manner. (**A**) Representative images of DAPI (blue) and MeCP2 (magenta) immunostaining of a small region in the MOp in *Mecp2*^+/−^ female mice. Scale bar, 10 μm. (**B**) UMAP of cell types (left) and genotypes (right) in the MOp of *Mecp2^+/−^* female mice. (**C**) Cross-correlation matrices of Chr6 in WT and *Mecp2* KO L4/5 IT neurons. (**D**) Histograms of local A/B density ratio changes upon *Mecp2* deletion for compartment-A (red) and compartment-B (blue) loci in various cell types. The corresponding boxplots for the distributions are shown at the bottom. Student’s *t* tests against null hypothesis of mean 0 with Bonferroni corrections were used for statistical significance evaluation. **P ≤ 0.01, ***P ≤ 0.001, ****P ≤ 0.0001; not significant (n.s.), *P* > 0.05. (**E**) Changes in A/B density ratio upon *Mecp2* deletion versus normalized radial position of the genomic loci in various cell types. Loci were grouped into 10 equal bins based on their radial positions in the WT cells. (**F**) Correlation between transcriptional changes and local A/B density ratio changes upon *Mecp2* deletion in excitatory neurons. Genes are grouped into 10 equal bins based on their local A/B density ratio changes. For each gene, the DE score for transcription change between KO and WT cells was calculated as in Fig. 5E using scRNA-seq data (*83*). For (E) and (F), the line and shaded area represent the mean and the 95% confidence interval, respectively.

Next, we investigated the role of MeCP2 on chromatin organization and how this is related to transcriptional regulation. To address these questions, we applied integrated RNA- and DNA-MERFISH to *Mecp2^+/−^* heterozygous female mice and imaged ~43,000 cells in the MOp. Because the *Mecp2* gene is on the X chromosome (*45*, *89*), and one of two X chromosome homologs in female mice is randomly inactivated during development (*90*), we anticipate that roughly half of the cells in *Mecp2^+/−^* mice would express *Mecp2* from the WT allele, and the remaining cells would not express *Mecp2* due to the KO allele. Through immunostaining of MeCP2 conducted in conjunction with MERFISH measurements on the same samples, we identified WT cells and *Mecp2* KO cells across different cell types (Fig. 6A and fig. S20A). *Mecp2* deletion did not significantly change the cell-type composition in the MOp (Fig. 6B and fig. S20B), nor did it change the size of the nucleus substantially, with only a subset of cell types exhibiting statistically significant but subtle changes in the nuclear volume (fig. S21).

Next, we characterized the chromatin organization changes upon *Mecp2* deletion in different cell types, with a focus on A/B compartmentalization. Ensemble analysis of A/B compartments by grouping all imaged cells across all cell types together showed little changes upon *Mecp2* deletion, with only a very small fraction of genomic loci showing different compartment identities between WT and *Mecp2* KO (fig. S22), consistent with previous bulk Hi-C results (*85*). However, we observed visually noticeable impairment of A/B compartment in certain cell types, such as L4/5 IT neurons, upon *Mecp2* deletion (Fig. 6C). To quantify this effect, we used cell type–specific A/B compartment assignment from the WT cells and examined the effect of *Mecp2* deletion on the local A/B-compartment environment of the imaged genomic loci using the local A/B density ratio analysis. We focused on the relatively abundant cell types (fig. S23). In neurons, compartment-A loci generally showed a decrease in their local A/B density ratios and compartment-B loci generally showed an increase in their local A/B density ratios (Fig. 6D). This suggests a stronger tendency of intermixing of compartment-A and compartment-B chromatin upon *Mecp2* deletion, consistent with the visual impression of reduced compartment strength (Fig. 6C). This effect was the strongest in upper-layer IT neurons, weaker in other neurons and astrocytes, but largely absent in oligodendrocytes and vascular cells (endothelial cells and pericytes) (Fig. 6D).

Because compartment-A and compartment-B chromatin are respectively enriched at the nuclear interior and the nuclear periphery, the above observations suggest that *Mecp2* deletion would cause a nuclear radial position–dependent change in the local A/B density ratio in affected cell types, with chromatin loci at the nuclear interior and periphery experiencing a decrease and increase in their local A/B density ratios, respectively. This was indeed observed (Fig. 6E and fig. S24). Consistent with the notion of increased intermixing of compartment-A and compartment-B chromatin upon *Mecp2* deletion, we also observed a change in the nuclear radial positions of the genomic loci, with centrally located chromatin loci generally moving toward the nuclear periphery and peripherally located chromatin loci generally moving toward the nuclear interior in a similar cell type–dependent manner as that observed for the A/B density ratio changes (fig. S25).

The above results suggest that *Mecp2* deletion causes an increased intermixing of compartment-A and compartment-B loci. Because *Mecp2* deletion could also cause some loci to change compartment identities, this could give rise to an apparent increase in the intermixing of compartment-A and compartment-B loci when we fixed the compartment assignment based on WT cells because loci that changed from A to B compartment could exhibit a decrease in the A/B density ratio, and those that changed from B to A compartment could exhibit an increase in the A/B density ratio. We thus also conducted compartment calling on WT and *Mecp2* KO cells separately. The compartment PC values of the cross-correlation matrices were highly correlated between WT and *Mecp2* KO cells (fig. S26, A and B) and the vast majority (~90 to 95%) of loci conserved their compartment identities between the two genotypes (fig. S26C). While the loci with conserved compartment-A identity were substantially closer to the nuclear center than those with conserved compartment-B identity, the ones changed from A to B had a similar radial position distribution to those changed from B to A (fig. S26D). When considering only loci with conserved compartment identities, we recapitulated similar A/B-dependent and radial position–dependent changes in the A/B density ratio upon *Mecp2* deletion (fig. S26, E and F). Together, the above results showed that the radial position–dependent change in the A/B density ratio upon *Mecp2* deletion can be mainly attributed to increased intermixing of compartment-A and compartment-B chromatin.

Notably, the cell-type and nuclear radial position dependence in the local A/B density ratio changes was similar to that observed for changes in transcriptional activity of genes upon *Mecp2* deletion. We observed a strong and positive correlation between the changes in transcriptional activity of genes and their changes in local A/B density ratios induced by *Mecp2* deletion (Fig. 6F and fig. S26G). Upon *Mecp2* deletion, the loci that showed an increase in the local A/B density ratio also showed an up-regulation in transcription; the loci that showed a decrease in the local A/B density ratio also showed a down-regulation in transcription. Moreover, among the genes that were significantly up-regulated or down-regulated upon *Mecp2* deletion, only 6% of them changed compartment identity (fig. S26H). Therefore, the divergent transcriptional changes upon *Mecp2* deletion are mostly associated with increased A/B intermixing rather than A/B identity changes. We also observed a similar effect of *Mecp2* deletion in the visual cortex (~20,000 cells imaged), albeit to a smaller extent, likely due to the younger age of the mice that we imaged for the visual cortex study (fig. S27).

Overall, our data suggest a role of MeCP2 in regulating chromatin organization in neurons—MeCP2 promotes A/B-compartment segregation of chromatin. Upon *Mecp2* deletion, the degree of A/B-compartment chromatin segregation was weakened, the nuclear interior became a less active environment, and the nuclear periphery became a less repressive environment. This provides a potential mechanism to explain the divergent, radial position–dependent effect of *Mecp2* deletion on transcriptional activity, namely, the transcription down-regulation for genomic loci located in the nuclear interior and up-regulation for genomic loci located at the nuclear periphery.

## DISCUSSION

In this work, we developed an integrated genome-scale RNA and DNA imaging method to identify cell types and determine cell type–specific 3D-genome organization in complex tissues. We applied this approach to study the mouse cortex and revealed a wide spectrum of nuclear architecture differences among different brain cell types, including differences in the cell-nucleus sizes, chromatin 1D-to-3D packaging and scaling, higher-order chromosome structures, and nuclear radial positioning of chromatin. We further observed strong correlations of these cell type–dependent variations in nuclear architecture and 3D-genome organization with transcriptional regulation, in terms of both the overall transcriptional activity of the cells and the differential expression of specific genes between cell types. Furthermore, our studies provided insights into how MeCP2, the direct causal gene of Rett Syndrome as well as some other neurological disorders (*43*–*49*), regulates the 3D-genome organization and transcription in neurons.

First, we observed that different cell types in the brain exhibited substantially different cell-nucleus sizes (Fig. 1D), which expands the previous knowledge of cell-type dependence in the cell-nucleus size at a lower cell-type resolution (*36*, *68*, *69*). Moreover, our results revealed a strong and quantitative correlation between the transcriptional activity and cell-nucleus size (Fig. 1, D to F). In particular, both the cell-nucleus size and the transcriptional activity increased from non-neuronal cells to inhibitory neurons to excitatory neurons, and finer changes were also observed among subclasses of cells within these major cell classes. The increase in cell-nucleus size could make chromatin less compact and more accessible to transcriptional machinery, thereby increasing transcription. The higher transcriptional activity could also lead to larger amounts of RNA transcripts and transcription-related proteins and hence more nonchromatin space occupied by these molecules, thereby increasing the cell-nucleus size. Together, these results suggest that the size of the cell nucleus could be a hallmark of the cell types and states. In disease contexts such as cancer, alterations in the cell-nucleus size have been frequently reported and used as diagnostic markers (*91*). It is therefore possible that these disease-related changes in cell-nucleus size and gene expression are connected in a manner that is relevant to disease phenotypes.

As the cell-nucleus size increased from non-neuronal cells to neurons, we observed that chromatin was not simply decompacted in a uniform manner. Chromatin packaging and scaling differed among different cell types and varied with transcriptional activity in different manners at different length scales. (i) At the smallest length scale probed in this work (~1 to 2 Mb), chromatin locus pairs separated by the same genomic distances tended to exhibit smaller spatial distances in neurons than in non-neuronal cells (Fig. 2, E and F). Neurons, which have higher transcriptional activity than non-neuronal cells, likely have a higher frequency of enhancer-promoter interactions or other chromatin interactions such as gene-body domains (*30*, *35*, *92*), which may contribute to their tighter chromatin packaging at this length scale. (ii) At the intermediate length scale (<10 Mb), the spatial-vs-genomic distance scaling of chromatin followed a power law with a greater power exponent in neurons than in non-neuronal cells. This scaling power is positively correlated with transcription activity (Fig. 2, A to D). The higher accessibility and looser packaging of active chromatin may contribute to this scaling behavior. It is also possible that higher transcriptional activity in neurons may help accumulate more nonchromatin factors such as RNA transcripts and transcription-related proteins, separating chromatin loci spatially at this scale. (iii) In contrast, at the large length scale (>10 Mb), the spatial-vs-genomic distance scaling of chromatin followed a power law with a smaller power exponent in neurons than in non-neuronal cells, and this scaling power is negatively correlated with transcriptional activity (Fig. 2, A to D). This may be related to the previous observations in cultured cells that active-active chromatin interactions is more prominent than inactive-inactive chromatin interactions at larger genomic separations (*25*, *93*). We speculate that this long-range active-chromatin interaction may be facilitated by transcriptional condensates. It is remarkable that chromatin packaging and organization differ between neurons and non-neuronal cells in three distinct manners at three different length scales. The correlation that we observed between chromatin scaling and transcriptional activity here in the context of different cell types may also be a general phenomenon or principle that goes beyond the context of cell-type variations. Moreover, our result may also provide a physical picture explaining different preferences for short-range versus long-range chromatin contacts observed across various biological contexts in recent sequencing studies, ranging from cell-cycle progression to cell-type development and aging (*32*–*34*, *37*, *94*).

In addition to the cell type–dependent variations in chromatin scaling, we also observed pronounced differences in large-scale chromosome structures in different cell types. In non-neuronal cells, which exhibited relatively low overall transcriptional activity, chromosomes tended to form large domain structures (Fig. 3, A and B), which resembled the megadomains previously observed in the transcriptionally inactive X chromosomes (*21*, *23*, *72*–*74*). Megadomain formation in inactive X chromosomes is thought to be mediated by prevalent repressive epigenetic signatures, which compact heterochromatin into large domains separated by insulating elements (*73*, *74*). These megadomain structures contrast the A/B compartment structure observed in the active X chromosome (*23*, *73*), but whether this contrast extends beyond the context of X chromosome inactivation was previously unknown. We observed a consistent anticorrelation between the megadomain strength and the degree of A/B-compartment segregation across different cell types with varying transcriptional activity (Fig. 3, C and D). The strength of A/B-compartment segregation increased as the transcriptional activity increased from microglia and vascular cells to astrocytes and OPCs and lastly to neurons (Fig. 1E and fig. S11C). Together, these results suggest that transcriptional regulation may be generally linked to a counterbalance between megadomain and A/B-compartment structures.

We also observed a correlation between the local A/B-compartment environment and the differential activities of specific genes and gene-regulatory elements between different cell types (Fig. 4, E and F). We observed that genes differentially expressed between different cell types tend to be up-regulated in cell types where their local A/B chromatin density ratios were higher (in a relative sense as compared to other genes) (Fig. 4E), consistent with previous observations that differential expression of genes between cell types is correlated with their local CpG density in space (*32*, *33*, *38*). We also observed higher local A/B density ratios for cell type–specific super-enhancer in their designated cell types (Fig. 4F). The local enrichment for active chromatin could result in a higher local concentration of transcription machinery and activators, thereby enhancing the activity of these genes and enhancer elements. Alternatively, but not mutually exclusively, it is also possible that active promoters and enhancers tend to colocalize with each other, for example, through the formation of transcriptional condensates (*6*, *7*, *9*), thereby causing a higher local concentration of active chromatin.

Another difference in 3D-genome organization that we observed between different cell types was the nuclear radial positioning of active and inactive chromatin. While non-neuronal cells exhibited a strong correlation between nuclear radial positioning of chromatin loci and their accessibility and transcriptional activity, with active and inactive chromatin, respectively, residing in the nuclear interior and periphery, neurons exhibited a more intermixed organization of active and inactive chromatin along the radial axis of the nucleus (Fig. 5, A to D). The widespread up-regulation of gene expression in neurons may contribute to the relative enrichment of active chromatin in the nuclear periphery. Most of the active genomic loci near the nuclear periphery of neurons contain long genes related to neuronal functions (fig. S17), which are likely more capable of concentrating transcriptional machinery (*95*), potentially mitigating the repressive mechanisms in the nuclear periphery. In line with our observations, it has been shown recently that lamina-associated domains are less repressive in neurons than in cultured embryonic stem cells (*96*). In the meantime, up-regulation of transcription near the nuclear periphery may also weaken the tethering of some inactive chromatin to the nuclear envelope (*97*, *98*), causing its relocation to the nuclear interior. Both mechanisms may contribute to the observed increase in active-inactive chromatin intermixing along the radial axis in the nucleus of neurons.

Last, we observed that MeCP2 regulated both chromatin organization and transcriptional activity in a nuclear radial position and cell type–dependent manner (Figs. 5 and 6). Notably, *Mecp2* deletion caused a relative up-regulation of genes residing at the nuclear periphery and down-regulation of genes at the nuclear interior, and these effects were strongest in neurons and nearly absent in oligodendrocytes and vascular cells (Fig. 5E). In parallel, we observed that MeCP2 promoted local A/B-compartment chromatin segregation and upon *Mecp2* deletion, the nuclear interior showed a decrease in the local A/B chromatin density ratio, whereas the nuclear periphery showed an increase in the local A/B chromatin density ratio due to increased intermixing of compartment-A and compartment-B chromatin (Fig. 6). This role of MeCP2 in chromatin organization provides a potential mechanism that could naturally explain the radial position dependence in the transcription regulation by MeCP2.

The mechanism by which MeCP2 promotes A/B-compartment chromatin segregation remains an open question, but several properties of MeCP2 provide possible mechanisms. MeCP2 has high affinity to methylated DNA (*41*, *82*, *87*), a hallmark of inactive chromatin, and methylated DNA-bound MeCP2 could cluster DNA and form phase-separated condensates in vitro (*50*, *51*, *99*). Meanwhile, MeCP2 is also found to be enriched at active TSSs that have a low DNA methylation level (*42*), and MeCP2 can stably associate with nucleosomes harboring unmethylated DNA in vitro (*100*) and promote clustering of these nucleosomes (*101*). In addition, MeCP2 can recruit RNA polymerase II (*42*, *102*), which is also known to facilitate transcription condensate formation (*103*–*105*). It is thus possible that MeCP2 could facilitate separate clustering or phase separation of inactive and active chromatin. It has been shown in vitro that the heterochromatin condensates containing MeCP2 exclude transcription factors such as Brd4 (*50*), although it remains unclear whether a similar effect exists in vivo (*106*). Consistent with this notion, we observed that the radial position–dependent effect on transcription by *Mecp2* deletion is also recapitulated by the R168X mutant of MeCP2 (Fig. 5I), which removes the intrinsically disordered domain in MeCP2 and abolishes its ability to form condensates (*50*). In addition to this potentially direct involvement of MeCP2 in active-inactive chromatin segregation, it is also possible that *Mecp2* deletion could indirectly influence chromatin organization through affecting other regulatory factors of chromatin organization. Because neurons exhibited a more intermixed organization of active and inactive chromatin along the radial axis (Fig. 5, A to D), the expression of MeCP2 may be particularly important for preventing unwanted cross-talk between active and inactive chromatin and thereby ensuring proper expression of genes in neurons.

MeCP2 could also regulate transcription through other mechanisms. It has been shown previously that MeCP2 could repress transcription through recruiting the NCoR/SMRT corepressor complex (*80*). MeCP2 could also interact with transcriptional activators and RNA polymerase II and up-regulate transcription through this effect (*42*, *102*). These mechanisms could function together with the MeCP2’s effect in promoting active-inactive chromatin segregation, either independently or in an interdependent manner, to give the overall transcriptional regulation effect by MeCP2. We also note that our results established a correlation, but not causality, between the changes in chromatin organization and transcription upon *Mecp2* deletion. Hence, it is also possible that the transcriptional changes induced by *Mecp2* deletion caused the observed chromatin organization changes, or both mutually affect each other.

In summary, our integrated RNA- and DNA-MERFISH studies provide rich insights into cell type–specific 3D-genome organization and its connection with transcription regulation, both in normal brain function and in dysfunction caused by *Mecp2* mutations. The ability of this technology to determine cell type–specific 3D-genome organization in native tissues can be broadly applied to advancing our understanding of gene-expression regulation in health and in disease. We anticipate that future studies combining 3D-genome and transcriptome imaging with epigenome imaging (*107*) and protein imaging, along with cross-modality data integration through machining learning (*108*), could further enhance our ability to understand the inter-relationship between epigenetic properties of chromatin, gene-regulatory factors, chromatin structures, and transcription regulation. Moreover, combination of this approach with live-cell chromatin imaging (*109*) and with spatial manipulations of genomic loci by CRISPR-based methods (*110*) could further advance our ability to study the causal relationship between 3D-genome organization and transcription regulation with high spatiotemporal resolution.

## MATERIALS AND METHODS

### Animals

Three adult C57BL/6 male WT mice (Charles River Laboratories, catalog no. 027; RRID:IMSR_CRL:027) aged 57 to 63 days (three biological replicates) were used for investigating the cell type–dependent chromatin organizations in the mouse primary motor cortex (MOp). Two technical replicates were conducted for one of the three mice and one technical replicate was conducted for each of the remaining two mice. In each technical replicate, two brain slices were imaged.

*Mecp2* mutant mice (the Jackson Laboratory, catalog no. 003890; RRID:IMSR_JAX:003890) (*111*), including two adult *Mecp2* heterozygous mutant female (*Mecp2^+/−^*) mice aged 11 weeks and three adult Mecp2 heterozygous mutant female (*Mecp2^+/−^*) mice aged 27 weeks, were used for RNA-MERFISH combined with MeCP2 immunostaining. Among these mice, brain slices from two 11-week-old and two 27-week-old mice were further used for DNA-MERFISH imaging to investigate the effect of Mecp2 on chromatin organizations, constituting a total of two biological replicates for each age. As we showed in fig. S24, because of the limited number of biological replicates per age condition, it is difficult to conclude with respect to age-dependent effects. Because age dependence is not the focus of our manuscript, we grouped data from both ages in the analysis shown in our paper. Each biological replicate contained two brain slices from a distinct biological replicate.

Two adult Mecp2 heterozygous mutant female (*Mecp2^+/−^*) mice aged 8 weeks (two biological replicates) were used for integrated RNA- and DNA-MERFISH imaging of the visual cortex, together with MeCP2 immunostaining. Each biological replicate contained one technical replicate with one brain slice.

Animals were maintained on a 12-hour light/12-hour dark cycle (2:00 p.m. to 2:00 a.m. dark period), at a temperature of 22 ± 1°C, a humidity of 30 to 70%, with ad libitum access to food and water. Animal care and experiments were carried out in accordance with National Institutes of Health guidelines and were approved by the Harvard University Institutional Animal Care and Use Committee (animal protocol number: 10-16-3).

### Tissue preparation for integrated RNA-MERFISH and DNA-MERFISH

Mice were euthanized with CO_2_, and their brain was quickly harvested and frozen immediately in optimal cutting temperature compound (Tissue-Tek O.C.T.; VWR, 25608-930) in dry ice and stored at −80°C until sectioning. Frozen brains were sectioned at −18°C on a cryostat (Leica CM3050s). Slices were removed, and discarded until the MOp region was reached. Specifically, a continuous set of 10-μm-thick serial coronal sections of the brain were cut from anterior to posterior to include the brain regions of interest such as the MOp and its vicinity and visual cortex and its vicinity, according to Allen Institute reference map v3 (https://atlas.brain-map.org/atlas?atlas=602630314) (*112*), as previously described (*62*). Typically, every two slices that are ~100-μm apart along the anterior-to-posterior axis were collected onto one #1.5 round coverslip (Bioptechs, 0420-0323-2). Note that for all prepared WT tissues, only coverslips that were successfully imaged for both RNA-MERFISH and DNA-MERFISH were processed for downstream image analysis. In total, for WT tissues, four coverslips (containing eight brain slices total, covering the MOp and its vicinity, from three mice) were successfully imaged and analyzed. For *Mecp2^+/−^* mutant MOp tissues, 10 coverslips (containing 20 brain slices total, from five mice) were imaged with RNA-MERFISH and MeCP2 immunostaining, and 4 of these 10 coverslips (containing eight brain slices in total from four mice) were further completed for DNA-MERFISH imaging and analysis. For *Mecp2*^+/−^ mutant visual cortex tissues, one coverslip (containing two brain slices, each from one distinct biological replicate) were imaged with RNA-MERFISH, MeCP2 immunostaining, and DNA-MERFISH. In this study, we did not gel-embed and clear the brain tissues, and hence, the coverslips were not silanized for gel sticking (*62*, *63*), but we washed and cleaned the coverslips with 70% ethanol before collecting tissue slices.

After tissue slices were collected onto coverslips, tissue slices were fixed by treating with 4% paraformaldehyde (PFA) (Electron Microscopy Sciences, #15714) in 1 × PBS (Corning, 21–031-CV) for 10–12 minutes at room temperature, washed three times with 1 × PBS and then stored in 70% v/v ethanol in water at 4°C for at least 18 hours to permeabilize cell membranes. The tissue slices were either subsequently hybridized with MERFISH probes and imaged or were stored in 70% v/v ethanol at 4°C for no longer than 2 months before they were hybridized with MERFISH probes and imaged.

### Gene selection for RNA-MERFISH

We have previously identified cell types and mapped their spatial organization in the mouse MOp by imaging a panel of 258 genes, among which 242 genes were imaged using MERFISH, and the remaining 16 genes were imaged with eight sequential rounds of two-color FISH (*62*). In this work, to distinguish transcriptionally distinct cell types in the mouse MOp, we used the same panel of the 242 genes for the RNA-MERFISH run, as in our previous study (*62*). Even without the 16 remaining genes previously imaged by sequential rounds of FISH, we were able to identify all subclasses of cells, as identified in our previous study (*62*).

### Codebook and encoding probe design for RNA-MERFISH

Binary barcodes for the 242 genes were designed as previously described (*62*), which were drawn from a 22-bit, Hamming-Distance-4, Hamming-Weight-4 encoding scheme. The encoding probes for RNA-MERFISH were designed and obtained as described previously (*62*). In brief, each encoding probe contains a targeting sequence, which is complementary to a target region on a target RNA, and multiple readout sequences. We designed 22 readout sequences in total, each corresponding to one of the 22 bits. The collection of encoding probes targeting each gene contains a total of 4 readout sequences, corresponding to the 4 bits that read “1” in the barcode for that gene. Each encoding probe contains two of the four readout sequences encoding that gene. Unlike our previous study, where we detected these readout sequences directly with dye-labeled readout probes complementary to the readout sequences (*62*), in this study, we detected these readout sequences by adaptor probes followed by readout probes, as described in the “RNA-MERFISH imaging” section in Additional Materials and Methods in the Supplementary Materials.

### Genomic locus selection for DNA-MERFISH

We designed three different encoding probe libraries for DNA-MERFISH (and also sequential DNA-FISH) as described in the main text, targeting three groups of genomic loci, respectively. (i) The first encoding probe library targets 988 genomic loci that are approximately evenly distributed across the mouse genome with ~2.5 Mb of spacing (except the Y chromosome). (ii) The second library targets 28 genomic loci that are centered around TSSs for representative marker genes for different MOp cell types. These 28 marker genes are *Slc30a3*, *Slc17a7*, *Slc32a1*, *Gad1*, *Otof*, *Rspo1*, *Pvalb*, *Sst*, *Vip*, *Sncg*, *Lamp5*, *Lratd2*, *Tshz2*, *Syt6*, *Nxph4*, *Cux2*, *Rorb*, *Sulf2*, *Ptpru*, *Car3*, *Aqp4*, *Flt1*, *Igf2*, *Pdgfra*, *Sox10*, *Ctss*, *Vtn*, and *Bgn*. These 28 marker genes were previously shown as marker genes for distinct MOp cell types (*62*). (iii) The third library targets 965 genomic loci that are candidate cell type–specific super-enhancer loci for different MOp cell types. These candidate cell-type-specific super-enhancer loci were selected based on published snATAC-seq data of the mouse MOp (*66*). Specifically, for each MOp cell type identified previously (*59*, *66*), cell type–specific pseudo-bulk ATAC peaks were called using MACS2 (*113*), peaks that are within 12.5 kb of each other were stitched together and considered a super-enhancer locus, and peaks within 2.5 kb of a TSS were excluded. Then, cell type–specific candidate super-enhancers were called from these stitched peaks using the Rank Ordering of Super-Enhancers method (*64*, *114*) with a fitted cutoff score for each cell type. Because candidate super-enhancer peaks in different cell types may correspond to genomic regions that overlap with each other, we iteratively merged all cell type–specific super-enhancer peaks if the peaks identified in different cell types are within a 100-kb interval. For each merged candidate super-enhancer peak, we calculated the average of the overlapping ratio. This overlapping ratio is defined as the size of each individual peak divided by the size of the merged peak. We selected the merged candidate super-enhancer peaks whose average overlapping ratio is ≥0.8, which are either single peaks that are specific to a single cell type or peaks that are, on average, 80% shared among multiple cell types. Among these selected candidate super-enhancer peaks, those larger than 15 kb were kept.

For each genomic locus described above, we designed encoding probes against a 20-kb segment near or within the locus (Genome assembly GRCm38: mm10). Segments whose sequence allows for designing of ~50 encoding probes were kept as the final target genomic loci in our DNA-MERFISH measurements. Together, a total of 1981 genomic loci that were imaged in this study.

### Codebook design for DNA-MERFISH

Binary barcodes for (i) genomic-locus panel 1, the 988 genomic loci are approximately evenly distributed across the mouse genome, and (ii) genomic-locus panel 2, the 965 genomic loci that are cell type–specific candidate super-enhancers were designed separately but in the same fashion as described below. For genomic-locus panel 1, we first generated all possible 99-bit binary barcodes with a Hamming weight of 3 (i.e., each barcode containing three 1 bits and 96 “0” bits) based on the covering design (www.dmgordon.org/cover/). Then, we randomly selected 988 barcodes from this list iteratively for each chromosome to maximize the balance of on-bits over all bits and over all used barcodes within the certain chromosome (so that for each bit, we kept the barcoded loci reading 1 from the same chromosome as far from each other as possible) and maintained a Hamming distance of 4. In other words, this resulted in an approximately equal number of loci imaged per bit for each chromosome and balanced over all bits. Because of the polymeric nature of DNA, locus pairs on a given chromosome tend to localize physically together if their genomic distance is short. Therefore, we allowed loci within the same chromosome to exchange barcodes so as to optimize (i.e., maximize) the minimal genomic distance between loci with barcodes reading 1 at the same code position. When comparing code assignments with identical minimal genomic distances between loci with barcodes reading 1 at the same code position, we selected the code assignment that minimized the coefficient of variation of genomic distances between loci with barcodes reading 1 at the same code position (so that genomic distances between these loci have both larger means and smaller SDs).

Binary barcodes for the genomic-locus panel 2 (the 965 cell type–specific candidate super-enhancer loci) were chosen iteratively and similarly as above, except that they were from a list generated using all possible 95-bit binary barcodes.

For the genomic loci containing the TSS of the 28 marker genes (genomic-locus panel 3), we used two different imaging strategies. In the first strategy, the 28 loci were probed independently with sequential rounds of multicolor DNA-FISH, and this strategy was used for the WT mouse brains and the 11-week-old *Mecp*2^+/−^ female mouse brains. In the second strategy, to reduce the total imaging time, we imaged genomic-locus panel 3 together with genomic-locus panel 2 using a single MERFISH run. Specifically, 28 barcodes were selected from the 95-bit binary barcodes that were not used for encoding genomic-locus panel 2; we selected these barcodes randomly and iteratively to maximize Hamming distances of neighboring genomic loci, following the same design principle as described above for genomic-locus panels 1 and 2. This second strategy was used for imaging the 27-week-old *Mecp*2^+/−^ female mouse brains and the visual cortex of the *Mecp2^+/−^* female mouse brains. These two strategies for imaging the 28 TSS loci approaches generated nearly identical results.

### Encoding probe design for DNA-MERFISH

Encoding probes for DNA-MERFISH (and also sequential DNA-FISH) were synthesized from a pool of oligonucleotides purchased from Twist Biosciences whose sequences are listed in table S1. Each oligo in this pool consisted of the following subsequences (from 5′ to 3′):

1) A 20-nucleotide (nt) or 19-nt forward priming region for polymerase chain reaction (PCR) amplification and reverse transcription (RT).

2) A 20-nt readout sequence.

3) A 42-nt target sequence, designed to bind uniquely to a single targeted genomic locus without containing repetitive sequences.

4) Additional two 20-nt readout sequences.

5) A 20- or 19-nt reverse priming sequence for PCR amplification.

The forward and reverse priming sequences were chosen from a previously generated list of random 20-nt sequences optimized for PCR, as described previously (*25*).

We designed 222 readout sequences (99 for MERFISH imaging of genomic-locus panel 1, 95 for MERFISH imaging of genomic-locus panel 2, and 28 for sequential multicolor FISH imaging of genomic locus panel 3) that have been validated to have a good observed signal-to-noise ratio in total. In the case of DNA-MERFISH imaging, we assigned three different readout sequences (as subsequences #2 and #4 described above) for each genomic locus, corresponding to the 3 bits that read 1 in the barcode for that locus. In case of sequential multicolor FISH, we assigned one readout sequence for each color channel in each round, targeting one genomic locus (so that subsequences #2 and #4 described above have the same sequence). These readout sequences were chosen from a list of 30-nt sequences with minimal homology to the mouse (and human) genome, as described previously (*25*, *115*). We detected these readout sequences by adaptor probes followed by readout probes, as described in the “DNA-MERFISH imaging” section in Additional Materials and Methods in the Supplementary Materials.

The 42-nt target sequence was chosen similarly to a procedure described previously (*25*). Briefly, we repeated the following procedure for each genomic region of interest. First, we created a list of all 42-nt sequences complementary to the genomic region of interest (starting at each possible base in the targeted region). Then, sequences were filtered to ensure a GC content of 40 to 60% and a melting temperature of 57° to 67°C. These sequences were further filtered using BLAST (*116*) by limiting the allowed degree of homology to the mouse genome, the mouse transcriptome, and a database containing repetitive sequences using the same procedure as previously (*25*). Last, target sequences were selected from the remaining sequences after the final filtering step such that no genomic overlap exists between any pair of target sequences.

### Encoding probe synthesis

Encoding probes were amplified from the template library described above (see the “Codebook and encoding probe design for RNA-MERFISH” section and the “Encoding probe design for DNA-MERFISH” section above). This was done using a previously described amplification protocol (*25*, *54*) with additional modifications and involved the following steps. (i) The initial oligo pool was amplified using limited-cycle PCR for approximately 12 cycles. PCR primer sequences are listed in table S3. The reverse primer used in this step also introduced a T7 promoter sequence via primer extension. (ii) The resulting PCR product was purified via column purification (DNA Clean & Concentrator Kit, Zymo Research, D4033). (iii) The purified PCR product underwent further amplification and conversion to RNA by a high-yield in vitro T7-mediated transcription reaction (HiScribe T7 polymerase kit, NEB, E2050). (iv) The resulting RNA product was purified via column purification (Monarch RNA Cleanup Kit, NEB, T2050). (v) The purified RNA product was converted back to single-stranded DNA (ssDNA) by a RT reaction (Maxima H Minus Reverse Transcriptase, Thermo Fisher Scientific, EP0753) using the forward primer. For the DNA-MERFISH encoding probe library sets, a single deoxyuracil residue (dU) was optionally introduced to replace a thymine (T) in between 6 and 14 base where applicable in the forward primer for subsequent USER enzyme cleavage (NEB, M5505) that will be described below. (vi) The ssDNA product was subjected to alkaline hydrolysis to remove residual RNA and was subsequently column purified by ssDNA purification procedures (DNA Clean & Concentrator Kit with Oligo Binding Buffer, Zymo Research, D4032 and D4060). If dU was introduced in the PCR forward primer, then the ssDNA product was additionally cleaved by USER enzyme (NEB, M5505) before alkaline hydrolysis. (vii) The purified ssDNA product was dried in vacuum and resuspended in water to achieve the desired concentration of primary probe. All primers were purchased from Integrated DNA Technologies (IDT).

### Readout and adaptor probe preparation

All readout and adaptor probes were ordered from IDT (see tables S2 and S3) and were diluted directly from stock.

### Overview of experimental system of integrated RNA-MERFISH and DNA-MERFISH

Integrated RNA-MERFISH and DNA-MERFISH experiments were conducted using a home-built imaging platform, including steps of “RNA-MERFISH imaging, “sample positioning alignment preparation after RNA-MERFISH”, “sample positioning alignment for DNA-MERFISH”, and “DNA-MERFISH imaging”, described below in the “Overview of experimental protocol of integrated RNA-MERFISH and DNA-MERFISH” section.

The physical setup of the home-built imaging platform consists of several components. A custom-built fluorescence microscope was used to acquire images, and a custom-built fluidics system was used to automatically perform buffer exchanges on the microscope stage. Custom software was used to synchronize and control the various microscope and fluidic components, and to automate many experimental steps, as described previously (*25*). A detailed description of these components can be found in the “Experimental system of integrated RNA-MERFISH and DNA-MERFISH” section in the Supplementary Materials.

### Overview of experimental protocol of integrated RNA-MERFISH and DNA-MERFISH

Experimental protocol of integrated RNA-MERFISH and DNA-MERFISH experiments include following steps:

1) Encoding-probe hybridization for RNA-MERFISH.

2) RNA-MERFISH imaging.

3) MeCP2 immunofluorescence (only for *Mecp2 +/−* mice).

4) Sample positioning alignment preparation after RNA-MERFISH.

5) Sample wash after RNA-MERFISH.

6) Encoding-probe hybridization for DNA-MERFISH.

7) Sample positioning alignment for DNA-MERFISH.

8) DNA-MERFISH imaging.

9) Sequential DNA-FISH imaging.

A detailed description of these steps can be found in the “Experimental protocol integrated RNA-MERFISH and DNA-MERFISH” section in the Supplementary Materials.

### Overview of image processing pipeline for integrated RNA-MERFISH and DNA-MERFISH

The image processing pipeline for processing and decoding MERFISH images was implemented in Python (see Data and materials availability). The overall pipeline consists of the following steps and their detailed description can be found in the “Image processing pipeline for integrated RNA-MERFISH and DNA-MERFISH section” section in the Supplementary Materials:

1) Image corrections including bleed-through correction, chromatic-aberration correction, illumination-intensity uniformity correction.

2) Identify and segment all imaged cell nuclei from RNA-MERFISH imaging.

3) For RNA-MERFISH, follow standard pixel-based decoding to identify RNA molecules as described previously (*117*).

4) Translate the cell-nucleus segmentation from RNA-MERFISH images to DNA-MERFISH images.

5) Fit all detected DNA-MERFISH signals in imaging channels and determine corrected 3D coordinates based on drift correction.

6) Decode and assign identities to genomic loci and RNA molecules using custom algorithms and software.

### Overview of data analysis of integrated RNA-MERFISH and DNA-MERFISH

After MERFISH image processing steps described above, transcriptionally distinct cell types from the MOp were determined from RNA-MERFISH data using cell clustering analysis. To obtain genome-wide information of transcription and chromatin accessibility of these cell types, publicly available sequencing datasets such as RNA-seq and ATAC-seq of the matched cell types were used. The transcriptional activity of each imaged chromosome locus was estimated on the basis of RNA counts of genes that are close to the locus by genomic distance.

3D spatial coordinates of imaged chromosome loci from DNA-MERFISH were used to calculate cis-chromosomal pairwise spatial distance matrices for each chromosome from each cell type. In addition, cell type–specific 3D-genome structures across different physical scales, such as cell-nucleus volume, chromosome territory size, megadomains, A/B compartments, and radial positioning, were determined.

A detailed description of data analysis can be found in the “Data analysis for integrated RNA-MERFISH and DNA-MERFISH” section in the Supplementary Materials.

### Data and statistic details

The sample size (number of mouse brains and number of cells) was determined on the basis of our previous experiences of MERFISH studies of mouse brain tissues. The study of WT mouse brain tissues did not require the allocation of samples into different experimental groups. In the study of *Mecp2*^+/−^ mice, WT and *Mecp2* KO cells were grouped on the basis of the immunofluorescence signal using anti-MeCP2 antibody, although automated image analysis, and these cells were imaged in the same MERFISH batches. Hence, randomization and blinding were not needed. We did not exclude any data from consideration. All MERFISH images that were successfully decoded were included in the primary analysis.

During the primary analysis, all imaged cells were decoded. We filtered out a small fraction of cells with low quality in RNA-MERFISH measurement, whose cell type identity was thus not determined for subsequent analysis, as described in the “Cell clustering analysis of RNA-MERFISH data” section in Additional Materials and Methods in the Supplementary Materials. In addition, a fraction of cells did not have the whole nucleus included in a 10-μm-thick tissue slice, resulting in a partial sectioning of the cell nucleus. Thus, for analysis of cell-nucleus size, we only considered cells containing at least 1250 loci to estimate this metric more accurately, as described in the “Estimate of nuclear volumes of cells from DNA-MERFISH” section in Additional Materials and Methods in the Supplementary Materials. However, in the *Mecp2^+/−^* datasets, given the lower cell number, a threshold of 600 decoded loci was used for nuclear volume estimation; although the absolute cell-nucleus volume may be less accurately determined by choosing a smaller cutoff in the number of decoded loci, the relative differences in cell-nucleus volumes between *Mecp2* WT and KO cells may be more accurately assessed with more cells kept in the analysis. In any case, we did not make any significant conclusion on whether the cell-nucleus volume changed upon *Mecp2*. For analysis of median radial positions and A/B density ratios, we only considered cells containing at least 600 decoded loci, as described in the “Local A/B density ratio” section and the “Radial positioning analysis for imaged genomic loci from DNA-MERFISH data” section in Additional Materials and Methods in the Supplementary Materials.

Statistical analysis was performed using Python’s SciPy package. One-way analysis of variance (ANOVA) with Tukey’s post hoc multiple comparison was performed between three or more conditions (e.g., cell types), as in Figs. 1D and 4B, and fig. S7A. Student’s *t* tests against null hypothesis of mean 0 with Bonferroni corrections were used for analyses in Fig. 6D and figs. S26E and fig. S27C. Mann-Whitney *U* test, corrected by the Benjamini-Hochberg method, was used for analyses in fig. S21. For boxplots in this study, the center line, box, and whisker represent the median, 25th to 75th percentile, and 5th to 95th percentile, unless otherwise specified in the figure legend.

## Supplementary Materials

### The PDF file includes:

Additional Materials and Methods Figs. S1 to S27 Legends for tables S1 to S3 References
