# Supplementary material for: Cell type–specific 3D-genome organization and transcription regulation in the brain
Source: Sci Adv. 2025 Feb 26;11(9):eadv2067. doi: 10.1126/sciadv.adv2067 (PMC11864200; doi:10.1126/sciadv.adv2067)
Supplement: Supplementary file 1 — Additional Materials and Methods Figs. S1 to S27 Legends for tables S1 to S3 References [file sciadv.adv2067_sm.pdf]

Science Advances
AAAS

Supplementary Materials for

Cell type–specific 3D-genome organization and transcription regulation in the brain

Shiwei Liu *et al.*

Corresponding author: Xiaowei Zhuang, zhuang@chemistry.harvard.edu

Sci. Adv. **11**, eadv2067 (2025)
DOI: 10.1126/sciadv.adv2067

The PDF file includes:

Additional Materials and Methods
Figs. S1 to S27
Legends for tables S1 to S3
References

Other Supplementary Material for this manuscript includes the following:

Tables S1 to S3

Additional Materials and Methods

Experimental system of integrated RNA-MERFISH and DNA-MERFISH

Microscope setup for Image acquisition

Image acquisition was performed using a custom-built microscope system, as previously described (*25*) but with some modifications. The system was built around a Nikon Ti-U microscope body with a Nikon CFI Plan Apo Lambda 60x oil immersion objective with 1.4 NA. The system also included a Nikon 10x objective for quick identification of the sample position under the microscope. Illumination of samples was through a Lumencor CELESTA light engine (a fiber-coupled solid-state laser-based illumination system) with the following wavelengths: 405 nm, 477 nm, 546 nm, 638 nm, and 749 nm. This system was used with a penta-bandpass dichroic (IDEX, FF421/491/567/659/776-Di01-25x36) and a penta-bandpass filter (IDEX, FF01-441/511/593/684/817-25). A scientific CMOS camera (Hamamatsu FLASH4.0 or Hamamatsu C13440 with factory calibration for single-molecule imaging) was used for image acquisition. Each camera field of view (FOV) consisted of 2048 x 2048 pixels, with a camera pixel corresponding to 108 nm in the X and Y dimensions in the imaging plane for the 60x oil immersion objective with 1.4 NA. Sample position in three dimensions was controlled using a XYZ stage (Ludl Electronic Products). A custom-built auto-focus system (*25*) was used to maintain a constant focal plane over prolonged periods of time. This was achieved by comparing the relative position of two IR laser (Thorlabs, LP980-SF15) beams reflected from the glass-fluid interface and imaged on a separate CMOS camera (Thorlabs, uc480).

These different components were controlled using a National Instruments Data Acquisition card (NI PCIe-6321, X Series DAQ) and custom software (see the “Software for controlling experimental components” section below).

Fluidics system configuration

The fluidics system consisted of several main components: a pump, a set of valves connected in series, a flow chamber in which the sample was mounted, and tubing and connectors. We used a peristaltic pump (Gilson, MINIPLUS 3) to generate flow in the system. The pump was connected to an array of 12-way valves (IDEX, EZ1213-820-4), connected in series. In this study, we used three 12-way valves connected in the following manner. Each valve’s last connection was used as the input of the next valve in the series (except for the last one), while the rest were connected to a tube containing the buffer for a single round of hybridization. A fixed subset of the valves were used for imaging, bleaching and wash buffers. This valve system was used to flow the various buffers into the FCS2 flow chamber (Bioptechs, 060319-2), in which the sample was placed together with a 0.5-mm-thick flow gasket (Bioptechs, DIE# F18524). The chamber output was connected to a waste collection vessel, forming an open flow system. Components were connected using elastic plastic tubing, and connections were additionally sealed using a pressure adessive (Blu-tack). Put together, these components allow control of both the rate of fluid flow and of the type of fluid flowing at any given time.

The system was controlled using custom software (see the “Software for controlling experimental components” section below). Overall, this system allowed for 24 rounds of hybridization (depending on the number of valves and the number of spots reserved for special

buffers). We constructed a bypassing flow circuit by a tubing connecting two 3-way adaptor sets (Bioptechs, 162003-1) that were added to each side of the FCS2 flow chamber (Bioptechs, 060319-2). Therefore, in experiments where the number of hybridization rounds exceeded the capacity of the flow system, we replaced the buffers via the following procedures: (1) We turned the manual valves on the 3-way adaptor sets to short-connect, bypassing the sample-containing chamber. (2) All valves were washed using 30% v/v formamide (ThermoFisher, AM9342) in water and then with double-distilled water. (3) The new set of buffers was introduced, and the chamber was reconnected to the flow system to allow for the next round of hybridization.

Software for controlling experimental components

All system components were controlled using custom-built software as described previously (*25*). This software package is composed of several main modules that work in concert:

"Hal" is the software package used to control and synchronize all illumination and microscope components. We note that in some cases it is necessary to write drivers for components which are not included in this package. Hal is also used to define imaging parameters, such as illumination light intensity, sequence of stage and illumination operations during imaging (e.g., during a z-scan), exposure time etc.

"Steve" is a module used to take mosaic images (i.e., a composite image that is made up of many individual fields of view (FOV)) of the sample and to select FOV for imaging in experiments.

"Kilroy" is the software used to control the fluidics components, and to define pre-programmed sequences of operations to be performed as sets (e.g., the set of operations that happens when a new round of hybridization is performed).

"Dave" can issue commands to both Hal and Kilroy and is used to automate the performance of data collection by defining in advance a complete set of fluidics system and microscope operations, the order and time-lag in which they are to be performed.

The general flow of an experiment is that, before the experiment starts, Hal and Kilroy are loaded with the parameters and specifications to be used. After the sample is loaded and the chamber is filled with the imaging buffer, a mosaic image of the DAPI channel is taken using Steve, and FOVs of interest are selected. A file is then generated to specify the sequence of operations throughout the entire experiment and is loaded to Dave, together with the coordinates of the selected regions of interest. The rest of the experiment is run automatically, without manual intervention. If the number of rounds in the experiment exceeds the capacity of the flow system, the automatic sequence specifies actions up to the capacity of the system. The buffers are then replaced, a new Dave file is created, and this is repeated until all rounds of imaging are completed.

Experimental protocol of integrated RNA-MERFISH and DNA-MERFISH

Encoding-probe hybridization for RNA-MERFISH

After tissue preparation (as described in the "Tissue preparation for integrated RNA-MERFISH and DNA-MERFISH" section above), the prepared tissue slices were hybridized with the RNA-MERFISH encoding probe set as previously described (*62*), except for that tissue slices were not

gel-embedded and cleared. Briefly, the samples were removed from the 70% v/v ethanol and washed with 2×saline sodium citrate (SSC) (ThermoFisher, AM9763) three times. Then, tissue slices were illuminated by multi-band light emitting diode arrays for three hours to reduce the autofluorescence background (*56*), which is critical in consideration of the omission of the tissue clearing step. Next, we equilibrated the samples with the wash buffer, containing 2×SSC and 30% v/v formamide) (ThermoFisher, AM9342), for 30 min at room temperature. The wash buffer was then aspirated from a coverslip, and the coverslip was inverted onto a 50-μl droplet of the encoding-probe mixture on a parafilm-coated Petri dish. The encoding-probe mixture comprised approximately 1 nM of each encoding probe for RNA-MERFISH, and 1 μM of a polyA-anchor probe (IDT) in 2×SSC with 30% v/v formamide, 0.1% wt/v yeast tRNA (ThermoFisher, 15401011) and 10% v/v dextran sulfate (Sigma, D8906). We then incubated the sample at 37 °C for 36–48 h. The polyA-anchor probe contained a mixture of DNA and LNA nucleotides (synthesized from IDT) (/5Acryd/TTGAGTGGATGGAGTGTAATT+TT+TT+TT+TT+TT+TT+TT+TT+TT+T, where T+ is locked nucleic acid, and /5Acryd/ is 5' acrydite modification), which can hybridize to the polyA sequence on the polyadenylated mRNAs, as previously described (*62*). The polyA-anchor probe was mainly used for MERFISH detection of the cellular total RNAs. After hybridization, the samples were washed with the wash buffer, containing 2×SSC and 30% v/v formamide, for 30 min at 47 °C for a total of two times to remove excess encoding probes and polyA-anchor probes. The coverslips were then washed three times with 2×SSC and were post-fixed with 4% PFA in 2×SSC for 10 minutes at room temperature. The coverslips were then washed three times with 2×SSC and were incubated with yellow-green fiducial beads (FluoSpheres™ Carboxylate-Modified Microspheres, ThermoFisher, F8803) in 2×SSC for 10 minutes at room temperature. Lastly, the coverslips were washed once with 2×SSC and then stored at 4 °C in 2×SSC supplemented with 1:100 murine RNase inhibitor (NEB, M0314S) for no more than two days, before subsequent RNA-MERFISH imaging.

RNA-MERFISH imaging

After encoding-probe hybridization of samples, we assembled the sample into the FCS2 flow chamber (Bioptechs, 060319-2) and performed RNA-MERFISH imaging of the target genes using the home-built imaging platform, as described in the "Overview of experimental system of integrated RNA-MERFISH and DNA-MERFISH" section (*25*). All fluid exchanges involved in this part of the protocol were performed using a custom-built fluidics system, as described in the "Fluidics system configuration" section. For each coverslip, typically 100-300 FOVs from two coronal brain slices were selected for MERFISH imaging. Nucleus staining by DAPI was used to help select FOVs of interest.

During the MERFISH imaging course, we performed multiple rounds of hybridization and imaging, each round reading out two bits in the barcode. For each round, the samples were imaged as follows unless otherwise described. (1) The sample was hybridized with a set of oligonucleotide probes termed "adaptor probes" that converts bit-specific readout sequences on the encoding probes to common readout sequences. For example, for a MERFISH experiment with 22-bit Hamming-weight 4 barcodes, we designed 22 readout sequences, each of which corresponds to one of 22 bits. The collection of encoding probes targeting each gene would contain a total of 4 readout sequences, corresponding to the 4 bits that read "1" in the barcode for that gene. We then designed 22 adaptor probes, each comprising two parts: one part having a

sequence that is complementary to one of the bit-specific readout sequences and the other part having two copies of a common readout sequence. We designed two common readout sequences in total, each corresponding to one imaging color channel. During each round, we added two different adaptor probes, corresponding to two bits, converting the two corresponding readout sequences on the encoding probes to the two common readout sequences. The adaptors probes were added at 100 nM in the adaptor hybridization buffer containing 2×SSC, 30% v/v formamide (ThermoFisher, AM9342), for 10 minutes at room temperature. (2) The sample was washed with the wash buffer containing 2×SSC and 30% v/v formamide (ThermoFisher, AM9342), for 3 minutes at room temperature. (3) The sample was then hybridized with a set of fluorescently labeled oligonucleotide probes termed "common readout probes" (synthesized by IDT), each common readout probe with a sequence that is complementary one of the common readout sequences on the adaptor probes. The fluorescence dyes were linked to the common readout probes via a disulfide bond. The common readout probes were added at 20 nM in the readout hybridization buffer containing 2×SSC, 30% v/v formamide (ThermoFisher, AM9342) and 0.005% v/v Triton-X (Sigma, T8787), for 15 minutes at room temperature. (4) The sample was washed again with the wash buffer containing 2×SSC and 30% v/v formamide (ThermoFisher, AM9342), for 3 minutes at room temperature. (5) The sample was then incubated and kept in imaging buffer comprising 5 mM 3,4- dihydroxybenzoic acid (Sigma, P5630), >50 μM trolox quinone (generated by UV radiation of a trolox solution; Sigma, 238813), 1:500 recombinant protocatechuate 3,4-dioxygenase (rPCO; OYC Americas, 46852904), 1:500 Murine RNase inhibitor (NEB, M0314L), 10 mM Tris-HCl (ThermoFisher, 15568025), and 5 mM NaOH (to adjust pH to 8.0; Sigma, S2770) in 2×SSC. (6) Finally, the samples were imaged using a 60x oil immersion objective, and signals for the two common readout probes and fiducial beads were collected in the 750-nm, 650-nm, and 488-nm channels, respectively at a rate of ~5 Hz. All channels were imaged for 13 consecutive 1-μm-thick z-stacks for each FOV of interest as described above.

All rounds of imaging were carried out as described above except for the first and the last round. For the first round, before the imaging buffer step (5), the sample was additionally washed in 2×SSC once and then incubated in 2×SSC containing 1 μg/mL DAPI (ThermoFisher, D1306) for 10 minutes to stain nuclei. Signal of DAPI was additionally collected in 405-nm channel, and either 405-nm channel or all channels in this round were imaged for 50 consecutive 0.25-μm-thick z-stacks for each FOV of interest. For the last round, adaptor probes targeting the polyA-anchor probes were added at 10 nM to stain the cellular total RNAs. Signal of polyA-anchor probes was collected in 750-nm or 650-nm channel, and it was imaged for either 13 or 50 consecutive 1-μm-thick z-stacks for each FOV of interest.

Between each imaging round, the signal from the previous round was extinguished. This was achieved by incubating the sample in the cleavage buffer comprising 2×SSC, 30% formamide and 50 mM Tris (2-carboxyethyl) phosphine (TCEP; Sigma, 646547) for 10 minute at room temperature, which cleaved the disulfide bond connecting fluorophores to readout probes. The cleavage buffer also contained 167 nM unlabeled common readout probes, which blocked unoccupied common readout sequences on the adaptor probes from interfering with the next round of hybridization. After incubation with the cleavage buffer, the sample was washed with the hybridization wash buffer containing 2×SSC and 30% v/v formamide (ThermoFisher, AM9342), for 3 minutes at room temperature, before the next round of hybridization of adaptor probes. The same cleavage buffer incubation and wash were also employed after the last round

of RNA-MERFISH imaging to remove any remaining readout signals from RNA-MERFISH.

Immunofluorescence imaging for MeCP2

For imaging tissue sections from *Mecp2* +/- mutant female mice, the samples were additionally stained and imaged for antibodies against MeCP2 after RNA-MERFISH. The following procedures for MeCP2 antibody staining and imaging were performed using the home-built platform as described above for RNA-MERFISH. Specifically, (1) the sample was washed twice with 1×PBS (Corning, 21-031-CV) after RNA-MERFISH. (2) The sample was then incubated in the blocking buffer containing 1×PBS, 5% m/v bovine serum albumin (BSA; Jackson ImmunoResearch, 001-000-162), and 0.5% v/v Triton-X (Sigma, T8787), for 30 minutes at room temperature. (3) The sample was incubated with primary antibody against MeCP2 (rabbit anti-MeCP2, Cell Signaling, #3456; RRID:AB_2143849), 1:300 diluted in the blocking buffer described above, for 3-4 hours at room temperature. (4) The sample was then washed with 1×PBS three times, for 5 minutes each. (5) The sample was incubated with a fluorescently tagged secondary antibody (goat anti-rabbit conjugated with Alexa-647, Invitrogen, #A-21245; RRID:AB_2535813), 1:300 diluted in the blocking buffer described above, for 1 hour at room temperature. (6) The sample was then washed again with 1×PBS three times, for 5 minutes each. (7) The sample was incubated and kept in the imaging buffer as described above in the "RNA-MERFISH imaging" section above. (8) Finally, the samples were imaged using a 60x oil immersion objective, and signals for the secondary antibody and fiducial beads were collected in the 647-nm and 488-nm channels, respectively at a rate of ~5 Hz. All channels were imaged for 13 consecutive 1-µm-thick z-stacks for each FOV of interest as described above.

Sample positioning alignment preparation after RNA-MERFISH

In order to align the sample on the home-built imaging platform for both RNA-MERFISH and DNA-MERFISH, we took DAPI images of 10 FOVs under the 10x objective, either before or after RNA-MERFISH imaging. These 10 FOVs were selected from two coronal slices for each imaged coverslip in an approximately evenly distributed manner, and they were used as landmarks for aligning the sample orientation and positioning between RNA-MERFISH and DNA-MERFISH, which will be described below in the "Sample positioning alignment for DNA-MERFISH" section below.

Sample wash after RNA-MERFISH

After RNA-MERFISH (and immunofluorescence staining for *Mecp2* +/- mutant tissues when appropriate) and sample positioning alignment preparation, the sample was removed from the microscope. Next, the sample was washed with a harsh wash buffer containing 2×SSC and 60% v/v formamide (ThermoFisher, AM9342), for 30 minutes at 46 °C to remove bound probes from RNA-MERFISH. The sample was then washed with the hybridization wash buffer containing 2×SSC and 30% v/v formamide (ThermoFisher, AM9342) briefly. The sample was then washed three times with 2×SSC and was post-fixed with 4% PFA in 2×SSC for 10 minutes at room temperature. The sample was then washed three times with 2×SSC and then stored in 70% ethanol at 4 °C for at least 18 hours before subsequent DNA-MERFISH.

Encoding-probe hybridization for DNA-MERFISH

After RNA-MERFISH and sample wash, we typically stored the sample in 70% ethanol before DNA-MERFISH imaging. For DNA-MERFISH, the sample was removed from the 70% ethanol and washed with 2×SSC three times. Then, the sample was illuminated by the multi-band light emitting diode arrays for three hours to reduce the autofluorescence background, as what was done before RNA-MERFISH imaging (*56*). Next, the sample was then post-fixed with 4% PFA in 2×SSC for 10 minutes at room temperature. This post-fixation is important to preserve the nucleus structure during the following steps including heat denaturation.

After post-fixation, the sample washed with 1×PBS (Corning, 21-031-CV) three times and was incubated in a 1×PBS solution containing 0.1% of sodium borohydride (Sigma, 452882) to reduce autofluorescence background. Then, the sample was treated with 0.5% v/v Triton-X (Sigma, T8787) in 1×PBS for 10 minutes at room temperature, followed by three times of 1×PBS wash. Samples were treated with 0.1 M hydrochloric acid (HCl; Sigma, H9892) for 5 minutes at room temperature to increase the target DNA accessibility, presumably by depurinating DNA and denaturing histones, followed by washes in 1×PBS 2-3 times. Samples were then treated with a solution of 0.1 mg/mL RNase A (ThermoFisher, EN0531) dissolved in 1×PBS for 30-45 minutes at 37 °C, to remove potential sources of off-target binding to RNA. Following this treatment, cells were incubated in pre-hybridization buffer, consisting of 2×SSC (ThermoFisher, AM9763) and 50% formamide (ThermoFisher, AM9342) for approximately 30 minutes. Next, the cell coverslip was inverted and placed on a drop of 50 µL of hybridization buffer (2×SSC, 50% v/v formamide, ThermoFisher, AM9342, 10% dextran sulfate, Sigma, D8906) containing a mixture of encoding probes at ~1 nM of each encoding probe for the DNA-MERFISH run, with 5 µg mouse Cot-1 DNA (ThermoFisher, 18440016) in a 60-mm petri dish. The dish was partially submerged in a water bath at ~86 °C for 3 minutes and incubated at 47 °C in a humidified chamber for 36-48 h. After incubation with encoding probes, the sample was washed in 2×SSC and 30% v/v formamide (ThermoFisher, AM9342), for 30 minutes at 47 °C for a total of two times to remove excess encoding probes. The coverslips were then washed three times with 2×SSC and were post-fixed with 4% PFA in 2×SSC for 10 minutes at room temperature. The coverslips were then washed three times with 2×SSC and were incubated with yellow-green fiducial beads (FluoSpheres™ Carboxylate-Modified Microspheres, ThermoFisher, F8803) in 2×SSC for 10 minutes at room temperature. Lastly, the coverslips were washed once with 2×SSC and then stored at 4 °C in 2×SSC for no more than two days, before subsequent DNA-MERFISH imaging.

Sample positioning alignment for DNA-MERFISH

After the encoding-probe hybridization step, we assembled the sample into the FCS2 flow chamber (Bioptechs, 060319-2) in a similar manner as for RNA-MERFISH so that the relative orientations of the sample to the microscope stage coordinate system were roughly the same for both RNA-MERFISH and DNA-MERFISH by visual comparison. Next, we incubated the sample with 2xSSC containing 1 µg/mL DAPI (ThermoFisher, D1306) for 10 minutes to stain the cell nuclei and we used the 10x objective to locate the 10 landmarks that we selected in the "Sample positioning alignment preparation after RNA-MERFISH" section. Based on these landmarks and also the general morphological features of brain slices, we further manually rotated the flow chamber to match the relative angle of the sample to the microscope stage

coordinate system by visual comparison. Then, we calculated the rigid XY rotation and translation matrix from the original centroid coordinates of these 10 selected FOVs (recorded during the step described in the "Sample positioning alignment preparation after RNA-MERFISH" section) to their new centroid coordinates (recorded during this step) by singular-value decomposition (SVD), acknowledging the fact that the tissue underwent affine transformation between RNA-MERFISH and DNA-MERFISH imaging. We repeated the above flow chamber rotation step until the calculated rotation angle was as close as possible to 0 degree. We kept the last calculated rigid XY rotation and translation matrix and used it to locate the same FOVs where we acquired images for RNA-MERFISH. Lastly, we took DAPI images of 4-5 FOVs among these FOVs using the 60x objective. We calculated the average XY shift between the DAPI images from RNA-MERFISH and DAPI images for these 4-5 FOVs and offset the reference point (0, 0) in our microscope setting by the calculated average XY shift.

DNA-MERFISH imaging

After locating the FOVs of interest where we acquired images for RNA-MERFISH, we performed DNA-MERFISH imaging for all targeted genomic loci in a similar manner as described in the "RNA-MERFISH imaging" section and also described previously (*25*). All fluid exchanges involved in this part of the protocol were similarly performed using a custom-built fluidics system, as described in detail in the "Fluidics system configuration" section.

As in RNA-MERFISH imaging, we perform multiple rounds of hybridization and imaging for DNA-MERFISH, each round reading out three bits in the barcode. For each round, the samples were imaged in the same manner as described in the "RNA-MERFISH imaging" section except for the following differences. (1) During the adaptor-probe hybridization step, the adaptor hybridization buffer contained 35% v/v formamide (ThermoFisher, AM9342) for DNA-MERFISH instead of 30% v/v formamide for RNA-MERFISH. When the sample was incubating with adaptor probes, we also performed a photobleaching step by illuminating each FOV with the maximum available light power of the 560-nm, 647-nm, and 750-nm wavelengths together for 3-5 seconds. As a result, the incubation time duration varied from 15 to 30 minutes depending on the number of FOVs to be photobleached. (2) During the imaging step, the imaging buffer did not need to include Murine RNase inhibitor. (3) Instead of imaging two common readout probes in two color channels per round as in RNA-MERFISH, we imaged three common readout probes in three color channels per round in DNA-MERFISH. The sample was imaged using the 60x oil immersion objective, and signals for the 3 common readout probes and fiducial beads were collected in the 750-nm, 650-nm, 561-nm, and 488-nm channels, respectively, at a rate of ~10 Hz. (Note that for the first round, where DAPI was additionally stained, the signal of DAPI was collected in the 405-nm channel, as what was done for RNA-MERFISH imaging.) (4) All channels, including 405-nm if used, were imaged for 50 consecutive 0.25-µm-thick z-stacks for each FOV of interest.

In addition to these differences above regarding each sequential round, there were also additional modifications for DNA-MERFISH imaging due to the relatively large number of imaging rounds compared to RNA-MERFISH. Because our fluidic system allowed loading of a maximum of 24 different hybridization solutions, we washed our fluidic system and replaced hybridization solutions when the number of hybridization rounds exceeded this capacity of the fluidic system. The fluidic system wash and buffer replacement procedures were described in the "Fluidics

system configuration" section. For long-duration sample imaging, we also performed a gentle post-fixation step with 2% PFA in 2×SSC for 5 minutes periodically between hybridization and imaging rounds (every ~3-4 days) to maintain the structural integrity of our sample (*118*).

For each DNA-MERFISH experiment, we typically first imaged 988 genomic loci that are uniformed spaced along the genomic coordinate (genomic-locus panel 1) over ~35 rounds of imaging, and then imaged 965 genomic loci of the super-enhancers (genomic-locus panel 2) plus, in some cases, the TSSs of the 28 marker genes (genomic-locus panel 3) over ~35 rounds of imaging.

Sequential DNA-FISH imaging

The TSSs of the 28 marker genes (genomic-locus panel 3) were either imaged by DNA-MERFISH together with the 965 genomic loci of cell-type-specific super enhancers (genomic-locus panel 2) as described above or were imaged separately by sequential round of three-color FISH. In the latter case, the encoding probes for genomic-locus panel 3 were co-hybridized with the DNA-MERFISH probes for genomic-locus panels 1 and 2, also at ~1 nM of each encoding probe. Sequential rounds of adaptor probe and common readout probes were then hybridized and imaged, in the same manner as described in the "DNA-MERFISH imaging" section, except that three genes, instead of three bits of the barcodes, were imaged in each round.

Image processing pipeline for integrated RNA-MERFISH and DNA-MERFISH

Image corrections for color-channel bleed-through, chromatic aberration, and non-uniform illumination intensities

Our MERFISH microscope setup used a penta-band dichroic mirror and emission filter set, and we observed bleed-through between signals collected from different wavelength channels (i.e., for different fluorophores). To correct for this bleed-through, we devised a bleed-through correction approach by staining a set of samples with one single type of fluorophores whose emission corresponded to one of our microscope channels, and subsequently measuring the signal bleed-through in other channels. Given this bleed-through is approximately a fixed linear profile, we linearly decomposed the signal intensities from different channels at each pixel to correct for bleed-through between channels.

Because DNA-MERFISH aims to determine the precise XYZ coordinates (~ 50-100 nm precision) of the genomic loci of interest, we also performed correction of chromatic aberration among different imaging channels. Bleed-through and chromatic aberration corrections for multi-color DNA-MERFISH imaging were performed by labeling the same set of genomic loci in each different imaging channel independently and by next comparing the location of signals of the same loci in the different color channels, respectively, as described previously (*25*). This shift in X and Y axes between each pair of channels is linearly correlated with X and Y positions in the FOV, and is a constant in Z axis (*119*). Therefore, we performed a linear regression of this shift on X, Y and Z axes for each pair of channels to obtain a shift-correction function. Next, we either translated signals of all pixels from an acquired image to generate a corrected image using this shift-correction function, or we kept this shift-correction function and applied it to the 3D fitted coordinates (generated by the "Spot fitting for DNA-MERFISH and DNA-FISH imaging" section below).

To better compare the signal intensities of foci across each whole FOV and facilitate better segmentation of cells on the edge of FOV, we also applied an illumination-intensity uniformity correction for each FOV. To do this, we generated the averaged laser intensity profile for each channel, and we next divided the acquired images by these fixed averaged laser intensity profiles to correct for the illumination differences within each FOV.

Cell and cell-nucleus segmentation for RNA-MERFISH

For the MOp datasets from wild-type mice, images for DAPI representing the cell nuclei and PolyT anchoring probe representing the cytoplasm from RNA-MERFISH were used to identify individual nuclei and estimate nucleus boundaries. Specifically, images were first downsampled on the x, y planes from 2048x2048 pixels to 1024x1024 pixels. 13 z-stacks with 1 µm-step were used for 3D segmentation as follows. We employed a deep-learning-based cell segmentation algorithm Cellpose 2.0 with a pre-trained model "TissueNet2" (*120*), which effectively identified the cell nuclei in 3D. We used anisotropy value of 1000/216 and diameter of 30 in Cellpose 2.0 model evaluation. We then used the segmentation masks determined using Cellpose 2.0 as the seed and performed watershed over the polyT image to determine cytoplasmic segmentation.

For the datasets from *Mecp2* +/- mutant mice, images for DAPI from RNA-MERFISH were used for segmentation. Images were first downsampled on the x, y planes from 2048x2048 pixels to 1024x1024 pixels. 50 z-stacks with 250nm-step were used for 3D segmentation as follows. We employed a deep-learning-based cell segmentation algorithm Cellpose 2.0 with a pre-trained model "nuclei" (*120*). We used anisotropy value of 250/216 and diameter of 30 in Cellpose 2.0 model evaluation.

Decoding algorithm for RNA-MERFISH

RNA-MERFISH datasets were decoded by MERlin as previously described (*117*). Briefly, for each z plane, we aligned the images of different MERFISH imaging bits and generated overall normalization factors to obtain a normalized signal intensity vector for each pixel across all MERFISH bits. Based on this normalized intensity vector, we assigned the RNA identity for each pixel based on the MERFISH codebook we designed. Then, adjacent pixels that were assigned with the same barcodes were aggregated into putative RNA molecules, and then the list of putative RNA molecules was filtered to enrich for correctly identified transcripts as described previously (*62*), for a gross barcode misidentification rate at 5% using MERlin (*62*). All decoded RNA molecules were then assigned to each cell/nuclei segmentation based on their position relative to the segmentation mask (see the "Cell and cell-nucleus segmentation for RNA-MERFISH" section above), giving a cell-by-gene count matrix for the 242 genes measured by MERFISH. This cell-by-gene count matrix was next used for relevant analyses in the sections below.

Cell-nucleus segmentation transformation from RNA-MERFISH to DNA-MERFISH images

To obtain the correspondence of cell-nucleus segmentation between RNA-MERFISH and DNA-MERFISH images, we used the rigid XY rotation and translation matrix calculated in the "Sample positioning alignment for DNA-MERFISH" section to transform the cell-nucleus segmentations calculated from DNA-MERFISH images to align to those calculated from RNA-

MERFISH images. We also used this rigid XY rotation and translation matrix to transform the cell-nucleus images from DNA-MERFISH and aligned their z-position using the fiducial beads. Then, we calculated the shift between the transformed DNA-MERFISH cell-nucleus image and RNA-MERFISH cell-nucleus image. Specifically, we split each 3D stack of 2D images into 8 equal 3D volumes and calculated the 2D image cross-correlation for each 3D volume. We removed the outlier correlation value and obtained the median correlation as the final correlation. By minimizing the sum of square difference between the transformed cell-nucleus images from DNA-MERFISH and the experimentally measured cell-nucleus images from RNA-MERFISH, we obtained the corresponding cell-nucleus segmentation for DNA-MERFISH and use it for downstream analyses.

Spot fitting for DNA-MERFISH and DNA-FISH imaging

The following analysis pipeline was applied to each imaged FOV in order to obtain the 3D positions of all genomic loci of interest. For each acquired DNA image, diffraction-limited spots within each identified nucleus were fitted to a 3D Gaussian function to identify their center of mass and brightness above local background (*25*). To make analysis more manageable, we fixed the number of fitted spots per image, which would be retained for decoding, to 20,000 or fewer in the DNA-MERFISH images (>3-fold greater than the number of distinct loci expected without noise). For sequential DNA-FISH images of the 28 marker gene TSS loci, we fixed the number of fitted spots per chromosome per image to 3 or fewer.

The fitted spots were then used for identifying genomic loci and for determining their positions, as described in the “Decoding of fitted DNA spots” section below.

Drift correction

Fluidic exchange can cause sample position drift during MERFISH hybridization and imaging. To correct for this drift between hybridization rounds, we aligned images of fiducial beads acquired from different hybridization rounds by calculating cross correlations (using Skimage package function, “skimage.registration.phase_cross_correlation”). In DNA-MERFISH, the bead images were subsampled (usually as ¼ of x and y dimension of 3D image) and the 3D drifts were calculated iteratively until drifts calculated from 3 subsampled image pairs were within 1 pixel difference. The mean drift of these 3 selected drift values was then used for the drift correction of the 3D coordinates of each DNA spot between hybridization rounds.

Decoding of fitted DNA spots

After DNA spot fitting and drift corrections, fitted DNA spots were decoded according to their barcode assignment, as designed in “Codebook design for DNA-MERFISH” section above. Specifically, a list was generated for the drift- and aberration-corrected locations of all identified DNA spots in each bit-image (corresponding to a specific color channel in a specific round of imaging). For each detected spot DNA in every bit-image, we found all spots from other bit-images that were within a set cutoff distance (300 nm in x, y, and z) from the location of the given spot in the given bit image to from spot pairs across bits. To increase the recovery efficiency of all detected spots, we retained all spot pairs for further analysis, regardless of whether such spot pairs had other spot(s) from other bit-images that were within the set cutoff

distance. Moreover, each spot pair was given a score for validity based on their 3D distances, intensity levels, and intensity variations. A spot pair, if valid within our codebook design, will have a higher score for validity if they have closer 3D distances between them, higher intensities, and low intensity variations between them. We then iterated through all spot pairs in a descending order of the validity scores. For each spot pair, we iteratively searched all possible spot trios within the cut-off distance (300 nm) containing the examined pair. Like for spot pairs, we then calculated the validity score for each spot trio that corresponds to a genomic locus in our codebook. We selected the spot trio with the highest score and excluded this trio from further analysis. After the above steps, for the remaining spot pairs without an identified trio, we next assigned barcode identities to all such spot pairs (by allowing for 1-bit error correction), generating a table of all candidate DNA spots according to our barcode design scheme. All candidate DNA spots were then assigned to each segmented cell nucleus based on their 3D positions.

After barcode identity and cell-nucleus assignment, we used the previously published spatial genome aligner algorithm, "Jie" (*67*), to pick the spots for different chromosome homologs without assuming the number of chromosome homologs for each nucleus. This is because some cell nuclei may be incompletely included in the 10-μm-thick coronal sections and contain an uncertain number of homologs. Briefly, the "Jie" algorithm utilizes the Gaussian chain polymer model to estimate the probability of observing a physical distance between a locus pair given their genomic distance, and the DNA polymer fiber connecting decoded spots with the maximal probability is picked. We introduced several modifications to the "Jie" algorithm to best fit our library design and DNA imaging experiment. (1) We adjusted the threshold score calculation as follows. We considered a case where the physical distance between the pair of any two adjacent imaged loci was set as the σ value in the Gaussian chain polymer model. Then, we calculated the probability of observing such a polymer fiber and picked only DNA fibers with higher probability. (2) Moreover, we adjusted the penalty scores in Jie and included a larger penalty for potential fiber path that is more than 1.5 μm because the original penalty scores sometimes assigned DNA spots from two distant fibers to one chromosome homolog. (3) We also explored several sets of parameters in Jie to pick more candidate spots while keeping the recovered chromosome fibers as continuous as possible. These parameters led to 0 – 2 recovered chromosome homologs for each chromosome per nucleus for most of the nuclei, as expected. (4) After the spot picking by Jie, we added an additional spot swapping algorithm to swap spots between the detected chromosome homologs within each nucleus as follows. We calculated the physical distance of each spot to their neighboring regions' center of mass from each chromosome homolog and then progressively swapped all spots between the chromosome homolog groups to minimize their summed physical distance to their neighboring regions' center of mass. The selected spots were then used to determine the 3D positions of the targeted genomic loci, which were used to trace the chromatin structure.

Data analysis for integrated RNA-MERFISH and DNA-MERFISH

Cell clustering analysis of RNA-MERFISH data

After obtaining the cell-by-gene RNA count matrix from the "Decoding algorithm for RNA-MERFISH" section above, we preprocessed the matrix and performed cell clustering analysis to resolve distinct MOp cell types using the Scanpy pipeline (*121*), as described previously (*62*).

Preprocessing of the count matrix includes the following steps briefly described below. (1) Because the cell and cell-nucleus segmentation approach that we used can generate a small fraction of spurious artifacts, we removed the segmented cells that had a volume that was either less than 100 μm^3 or larger than 5000 μm^3. We also removed the segmented cells where less than 10 genes or 20 RNA counts were detected, which can arise either from segmentation artifacts or from poor imaging quality for a small number of FOVs due to the buffer flow issue during the RNA-MERFISH run. (2) We additionally removed potential doublets of cells using Scrublet (*122*): candidate cells with a doublet score higher than 0.3 were removed, which accounted for approximately 0.3% of the total cell number after step 1. (3) Next, we normalized the total RNA counts for each cell to the median total RNA counts of all cells. (4) After count normalization, the cell-by-gene count matrix was further log1p-transformed and converted to z-scores.

After preprocessing the cell-by-gene matrix above, we performed dimensionality reduction of the matrix using principal component analysis (PCA) and used the first 30 principal components. We then performed graph-based community detection in the 30 principal components space, with nearest neighborhood size parameter k = 10, as previously described (*62*). We ran the Leiden clustering method to cluster the cells with the resolution parameter r = 0.5, which identified 23 clusters that corresponded well to the MOp cell types at the subclass level obtained in our previous study (*62*). We next manually curated these identified 23 clusters to match the consensus MOp subclass labels by merging a few small clusters to their adjacent large clusters and by splitting two clusters into six smaller clusters using a higher Leiden resolution parameter r = 0.1. The basis for merging and splitting was obtained from both inspecting the marker gene expression in these identified clusters, and from performing one round of cell type correspondence analysis (see the “Correspondence between clusters identified by different MERFISH protocols” section below). For the visual cortex, cells were clustered in a similar way as in motor cortex and cell type correspondence analysis was conducted by using MapMyCells (RRID:SCR_024672) with the algorithm “Hierarchical Mapping” and with the reference(*123*). These curated clusters were then assigned into cell types at subclass level as described in the main text.

For presentation, Uniform Manifold Approximation and Projection (UMAP) was used to embed the cells in two dimensions using the same principal components that were used for clustering.

Correspondence between clusters identified by different MERFISH protocols

Correspondence between cell clusters identified by RNA-MERFISH in this study without tissue clearing (referred to as “uncleared RNA-MERFISH” below) and by RNA-MERFISH using tissue clearing in our previous study (*62*) (referred to “cleared RNA-MERFISH”) was assessed by running a neural-net classifier, as previously described (*62*). Briefly, the pre-normalized count matrices from the uncleared RNA-MERFISH in this study and the cleared RNA-MERFISH from the prior study were both log1p-transformed and z-scored. Then, the shared 242 genes measured in both studies were used to train a multi-layer perceptron (MLP) model, which was next used to predict cleared RNA-MERFISH cluster labels for each cell in the uncleared RNA-MERFISH dataset. As such, each cell in our uncleared RNA-MERFISH datasets had both a predicted cleared RNA-MERFISH cluster label and a cluster label determined from our *de novo* clustering above (see the “Cell clustering analysis of RNA-MERFISH data” section above). Cells were grouped based on their *de novo* cluster identity, and then the fraction of cells from a given *de*

novo cluster identity that were predicted to have each cleared RNA-MERFISH cluster label was then determined to generate a confusion matrix, as shown in fig. S2D. Likewise, the same classifier approach was used for assessing our initial cell clustering result before any manual curation, as described in the "Cell clustering analysis of RNA-MERFISH data" section.

Bulk RNA sequencing data analysis of wild-type mouse brains

The bulk RNA sequencing data was obtained from Ref. (*124*). For each gene from the shared 238 genes between the bulk RNA sequencing and the uncleared RNA-MERFISH data, the FPKM per gene from the bulk RNA sequencing was calculated. 231 out of the 238 genes whose FPKM is larger than zero in the bulk RNA sequencing were kept and log10-transformed. The log10-transformed FPKMs of these shared genes were compared to their average log10-transformed RNA count per cell from the uncleared RNA-MERFISH in this study, as shown in fig. S2B.

3D spatial distances between locus pairs from DNA-MERFISH data

For each decoded genomic locus that had been picked into a chromatin fiber, its 3D coordinate was calculated as the average of the fitted 3D Gaussian centers of the spot trio (or spot pair) decoded for that locus (see the "Decoding of fitted DNA spots" section above). The 3D spatial distance between any pair of genomic loci in each single cell was simply calculated as their Euclidean distance. The pairwise spatial distance matrix (hereafter referred to as pairwise distance matrix) can therefore be generated for all pairs of genomic loci of interest in each single cell, either at chromosome level (for all locus pairs from the given chromosome) or at the genome level (for all locus pairs across the mouse genome, including Chr1-19 and ChrX). To obtain the cell-type medians of the pairwise distance matrices, we aggregated pairwise distance matrices from single cells that belong to the same cell type. We then calculated the medians for each element in the pairwise distance matrices at chromosome level, as shown in Fig. 3A; fig. S3, A and C, fig. S9, and fig. S12B.

snm3C-seq data analysis of wild-type mouse brains

Cell-type-specific snm3C-seq data from the mouse neocortex was obtained from Ref. (*35*). The cells from the mouse neocortex subregion, as noted in the associated metadata from the paper, were used. To compare DNA-MERFISH data and snm3C data (as shown in fig. S3, A and B), we created bins for the snm3C-seq data centered around the genomic regions that we imaged with DNA-MERFISH and procured the corresponding pairwise contact matrix for single cells by summing the pairwise contact counts per bin from the snm3C-seq data. The bin size used was 500kb upstream to the start plus 500kb downstream to the end of each targeted imaged locus (which is about 15kb in length). We used a 500kb bin size in this case because the detected snm3C contacts are relatively sparse for long-range interactions. To obtain average pairwise contact matrices for selected MOp cell types, we aggregated pairwise contact matrices from single cells that belong to the cell type of interest and calculated the cell-type means for each element of the pairwise contact matrices, as shown in fig. S3A. The corresponding pairwise distances derived from DNA-MERFISH, and the number of contacts derived from snm3C were next compared by the Pearson correlation analysis and were plotted on the log10 scale for each chromosome for the cell types selected, as shown in fig. S3B.

Estimation of nuclear volumes of cells from DNA-MERFISH data

The volume of the cell nucleus was estimated by generating the minimal 3D convex hull surface (using Python's SciPy package) from the 3D locations of all decoded chromosome loci in a given cell. Because a fraction of cells did not have the whole nucleus included in a 10-µm-thick tissue slice, we only included cells that had a number of decoded chromatin loci that was larger than a set cutoff number of 1250. We picked this number by varying the cutoff number from 400 to 2500 (which were ~10% to 60% of the recovery efficiency of all 1981 targeted genomic loci assuming two copies per loci) and by inspecting the changes in the median of nucleus size for all L2/3 IT neurons. After fitting a polynomial function, we found that the median of the cell-nucleus size reached a turning point when increasing the cutoff number to 1250. A higher cutoff number than 1250 dramatically decreased the number of qualified cells and the remaining qualified cells started to contain more merged cell-nucleus objects ("doublets" as assessed by inspection of the ploidy number of chromosome homologs). Therefore, we used 1250 as the cutoff number when calculating the cell-type medians of the nuclear volume per cell for each MOp cell type, as shown in Fig. 1, D, F to H; fig. S6 and fig. S7B, unless otherwise mentioned.

Estimation of total RNA count of cells from RNA-MERFISH data

For each single cell, the unnormalized total RNA counts from 242 genes targeted by RNA-MERFISH were measured. To correlate the nuclear volume versus total RNA count at single-cell level, only cells that passed the above criteria for nuclear volume were analyzed, as shown in fig. S6.

snRNA-seq data analysis of wild-type mouse brains

The snRNA-seq data of MOp cell types from wild-type mouse brains was obtained from Ref. (*59*). The snRNA-seq 10x v3 data from this paper was used for our analyses because it was reported to have a higher number of genes recovered than other snRNA-seq methods in this paper (*59*). The cell-by-gene RNA count matrix from the snRNA-seq v3 data was preprocessed and analyzed for cell clustering similarly as described in the "Cell clustering analysis of RNA-MERFISH data" section above. The identified cell clusters showed a high correspondence to their original annotation in the paper (*59*). Thus, we directly used the original cell cluster annotation for procuring cell-type-specific RNA expression profiles because (1) this was derived from the consensus clustering results for all RNA-seq datasets in the prior paper (*59*) and (2) this was cross-validated from the cleared RNA-MERFISH from the prior study (*62*).

To calculate cell-type-specific RNA expressions for mouse MOp cell types, we used two different cell-by-gene RNA count matrices after basic filtering: which are (1) normalized, log-transformed, and z-scored RNA expression matrices, and (2) unnormalized RNA expression matrices. The normalized expression matrices were primarily used for standardized cell clustering as described above, and for DE gene analyses used in Fig. 4, E and F. Because we found that many aspects of chromatin organization changes were related to total transcription activity differences among cell types, the unnormalized expression matrices were used for most analyses in this study unless otherwise stated.

snATAC-seq data analysis of wild-type mouse brains

The snATAC-seq data of MOp cell types from wild-type mouse brains were obtained from Ref. (*59*). The original consensus cell cluster annotation derived from this paper was also used for procuring cell-type-specific ATAC expression profiles. The unnormalized cell-by-region ATAC count matrices were used for most analyses in this study unless otherwise stated.

Estimation of total transcriptional activity and chromatin accessibility per cell from wild-type mouse sequencing data

To estimate total transcriptional activity and chromatin accessibility per cell for MOp cell types, we used the unnormalized expression matrices from the "snRNA-seq data analysis of wild-type mouse brains" section and "snATAC-seq data analysis of wild-type mouse brains" section above, respectively. The number of unique molecular identifiers (UMIs) per cell captured from the corresponding sequencing analyses, which are typically also the total counts per cell of the unnormalized expression matrices, were used as total transcriptional activity and chromatin accessibility per cell. The cell-type medians of total transcription activity and chromatin accessibility per cell were then calculated for each cell type and shown in Fig. 1, E to G. To estimate the total number of accessible regions pe cell, the number of regions that had an ATAC read for each single cell were calculated and the corresponding cell-type medians were derived and shown in Fig. 1H.

Estimation of total transcriptional activity per cell in human brains

To estimate total transcriptional activity per cell for human brain cell types, we used two prior datasets as follows. The first dataset was the snRNA-seq from the human M1 brain region, which was obtained from Ref. (*70*). The original cell cluster annotation derived from this paper was used and adjusted to match the corresponding cell subclasses between human and mouse. The number of UMIs per cell captured were used to estimate the cell-type medians of total transcription activity per cell as described above for mouse brains, which are shown in fig. S5A.

The second dataset was the RNA-MERFISH data of 4000 genes in the human MTG brain region, which was obtained from Ref. (*56*). The original cell cluster annotation derived from this prior paper was used and adjusted to match the corresponding cell subclasses between human and mouse. The total RNA counts per cell detected from RNA-MERFISH were used to estimate the cell-type medians of total transcription activity per cell, as shown in fig. S5B.

Estimation of chromosomal territory size from DNA-MERFISH data

Chromosomal territory sizes in fig. S7 were estimated using the radius of gyration function of all decoded chromosome loci from any given chromosome. For each MOp cell type, the cell-type medians of chromosomal territory size were then calculated by aggregating all measurements of single chromosomes. Specifically, median chromosomal territory size for each chromosome type (e.g., Chr1-19 and ChrX) were calculated separately by aggregating chromosomes across individual cells in the given cell type that belonged to that chromosome type.

Chromosome territory segregation score from DNA-MERFISH data

Chromosomal territory segregation score in fig. S7C was used to quantify the degree of spatial separation between different chromosomal territories. To calculate this quantity, we adopted a previously developed method (*25*), which computes the spatial separation of chromosomal domains. Briefly, we treated two different chromosomes as two domains. Between two domains, we used median pairwise distances to first calculate an intra-domain distance distribution by considering all spatial distances between each pair of chromatin loci within the first domain and all distances between each pair of chromatin loci within the second domain. We then similarly calculated an inter-domain distance distribution by considering all distances between pairs of chromatin loci that reside in different domains. We then defined the segregation score as the median of all inter-domain distances divided by the median of intra-domain distances. Two highly intermixed domains (which are chromosome territories in this case) would have segregation score close to 1, while domains that are just contacting will have a segregation score of substantially larger than 2.

Estimation of chromosomal transcription per cell from wild-type mouse sequencing data

To estimate of chromosomal transcription (total transcript counts per chromosome) per cell, we used the unnormalized expression matrices as described in the "Estimation of total transcription activity and chromatin accessibility per cell from wild-type mouse sequencing data" section above, except for that genes were grouped by their genomic location on chromosomes. The corresponding total counts (UMIs) from genes for each chromosome per cell were then used to calculate the cell type medians of chromosomal transcription per cell, as shown in fig. S7, D and E; Fig. 2C.

Proximity frequency for genomic-locus pairs from DNA-MERFISH data

To calculate the proximity frequency for genomic-locus pairs, we first counted the number of locus pairs whose measured spatial distance in 3D was smaller than a set cutoff distance. The proximity frequency for a genomic-locus pair was determined by dividing the number of such qualified locus pairs with spatial distance smaller than the cutoff value by the total number of detected locus pairs for the same pair of target genomic loci. We then grouped the target genomic-locus pairs by their genomic distances, and determined distributions of the proximity frequency overall genomic-locus pairs included in each genomic-distance range for each cell type, as shown in Fig. 2F. We selected 750 nm as the set cutoff distance to match the cutoff distance used for other analyses (see the "A/B compartment analysis from DNA-MERFISH" section below). In addition, we also tested 500 nm as the cutoff distance, as in Ref (*25*), and we observed a similar trend as that shown in Fig. 2F.

Estimation of transcriptional activity and chromatin accessibility for each imaged DNA locus from sequencing data

To approximate the cell-type medians of transcriptional activity and chromatin accessibility for each imaged DNA locus, we used the unnormalized expression matrices from the "snRNA-seq data analysis of wild-type mouse brains" section and "snATAC-seq data analysis of wild-type mouse brains" section above, respectively. Specifically, we created bins centered around each

imaged locus and summed the number of all RNA or ATAC counts for each bin in single cells. Next, for each cell type, we aggregated single-cell measurements to calculate the cell-type median value of each locus. We used 4Mb as the bin size because we found that it generated the highest correlation with chromosome compartment principal component (PC) values derived from our DNA-MERFISH (e.g., fig. S10, see the "A/B compartment analysis from DNA-MERFISH" section below for details). We then analyzed the cell type medians of transcriptional activity or chromatin accessibility for each imaged locus of interest with other chromatin features that were measured by DNA-MERFISH, whose results were used in Fig. 2D; Fig. 4, B and C; Fig. 5, A to D; fig. S10; fig. S12; fig. S16; fig. S17; fig. S18 for example.

After obtaining transcriptional activity and chromatin accessibility for each imaged locus, we additionally grouped each locus into different categories (e.g., "high", and "low") based on the percentile thresholds (<25th, and >75th percentiles respectively) of the corresponding transcription activity or chromatin accessibility distribution of all imaged loci, as described and shown in the Fig. 5, A and C; fig. S17.

Scaling of spatial distances versus genomic distances from DNA-MERFISH data

For each chromosome and cell type, we fitted the cell-type medians of all pairwise spatial distances within the chromosome and the corresponding pairwise genomic distances by a 2nd order polynomial function. The fitted curves were plotted on the log-log scale, as shown in Fig. 2A and fig. S8. We further separated these spatial-distance-versus-genomic-distance curves into short-range and long-range regimes, using 10Mb as a genomic distance cutoff. For each regime, spatial distances and the corresponding genomic distances were fitted by a power-law function to derive the scaling power exponent, as shown in Fig. 2A and fig. S8. The scaling power for each chromosome and cell type at short-range and long-range scale were used for plotting the heatmap as shown in Fig. 2B and for correlating to chromosomal transcription (see the "Estimation of chromosomal transcription per cell from wild-type mouse sequencing data" section above) as shown in Fig. 2C.

In addition, we used locus transcriptional activity (see the "Estimation of transcriptional activity and chromatin accessibility for each imaged DNA locus from sequencing data" section above) to group genomic loci from all chromosomes across all cell types into 10 bins. Next, we selected locus pairs from the same bin and obtained the corresponding 10 groups of locus pairs. We determined scaling power for locus-pairs within each of the 10 groups, for short-range and long-range respectively, using 10Mb as a genomic distance cutoff, as shown in Fig. 2D.

Normalized insulation scores of megadomain-like structures from DNA-MERFISH data

Insulation score has been previously defined for megadomains in inactive X chromosomes in ensemble Hi-C (*73, 125*). We therefore used a similar method for megadomain analysis for our DNA-MERFISH data. Briefly, for each chromosome locus in a given chromosome, we selected upstream and downstream loci with a fixed window size (8 loci and ~10Mb). We treated these two chromatin regions, up- and down-stream of the selected locus, as two "domains". We then computed the insulation score between these two regions as described in the "Chromosomal territory segregation score from DNA-MERFISH" section above. We then defined the normalized insulation score as the difference between the median of inter-region distances and the median of intra-region distances normalized by the sum of these two median values.

Therefore, a normalized insulation score will always be between 0 and 1, with the value 1 indicating strong insulation between the two regions. With this definition of normalized insulation score, we applied a sliding window along the chromosome to calculate the normalized insulation scores for each genomic loci in a chromosome. The local maxima then represent the boundaries between potential megadomain-like structures in the given chromosome, as shown in Fig. 3A and fig. S9. Additionally, the interquartile range (IQR) of the normalized insulation scores for each chromosome was determined and the distribution of the IQR values across different chromosomes was used to estimate the prevalence of megadomain-like structures for each cell type, as shown in Fig. 3B.

Megadomain strength estimation for chromosomes exhibiting near-bipartite megadomains

Chromosomes in non-neuronal cells often exhibit two major megadomains. We estimated megadomain strengths for chromosomes that exhibited such bipartite megadomains in non-neuronal cells, such as endothelial cells or pericytes (see Fig. 3A and fig. S9 for example). For these chromosomes, we first computed the normalized pairwise distance matrices to remove the effect from the difference in chromosomal territorial size across different cell types. To do this, we first calculated the cell-type-specific median pairwise distance matrices as in the "3D spatial distances between locus pairs from DNA-MERFISH data" section. Then, we calculated the median value of all elements from each cell-type-specific median pairwise distance matrix for each MOp cell type and normalized the matrix by this median value such that this median value for every cell type was equal to that of the reference cell type (set as the endothelial cells). This procedure was to remove the effect from the difference in chromosome territorial size between cell types. Next, we quantified the strength of megadomain structure as the ratio of inter-domain distances over intra-domain distances using a fixed megadomain boundary (determined by the boundary in either endothelial cells or pericytes whichever was most evident using the pairwise spatial distance matrices and the insulation score profiles).

$$megadomain\ insulation\ strength = \frac{median\ of\ inter\ domain\ distances}{median\ of\ intra\ domain\ distances}$$

Using this approach, we determined the megadomain strength in each cell type, regardless of whether the structure was evident in this cell type, and these megadomain strength values were used in Fig. 3D.

A/B compartment analysis from DNA-MERFISH data

We performed A/B compartment analysis at major cell class level, including excitatory neuronal class, inhibitory neuronal class, oligodendrocyte-OPC class, vascular cell class (endothelial cells and pericytes), astrocyte class, and microglia class. The A/B compartment analysis includes the following steps.

First, we calculated the pairwise proximity frequency matrices for each major cell type. To do this, we counted the total number of measured distances between any given pair of target genomic loci from all single cells belonging to the given cell type. The proximity frequency for any given genomic-locus pair was then determined as described in the "Proximity frequency for genomic-locus pairs from DNA-MERFISH data" section, using 750 nm as the cutoff distance.

We selected 750 nm as the cutoff distance because (1) the Pearson correlation coefficient between the calculated proximity frequency matrices and snm3C-derived contact matrices (*35*) remained high for cutoff distances between 200 nm to 800 nm, and (2) the cutoff distance of 750 nm, close to the high-end limit of the 200-800 nm range, retained a higher baseline level of pairwise proximity frequency and produced a less noisy Pearson cross-correlation matrices for compartment analysis than those obtained using lower cut-off distances.

After we obtained the pairwise proximity frequency matrices for each major cell type as described above, we normalized these matrices by dividing the observed proximity frequency matrices over the expected proximity frequency matrices. The expected proximity frequency matrices were derived by calculating the expected frequency of observing a locus pair within 750nm distance as a function of their genomic distance with a DNA polymer model (*67*). This normalization is meant to remove the effect of genomic distances between target genomic loci such that the chromatin locus pairs that are closer to or farther from each other than expected from the polymer model can be identified.

Next, the Pearson cross-correlation matrices were calculated from the normalized proximity frequency matrices above, as previously described (*25*), as shown in Fig. 3C. The Pearson cross-correlation matrices were then subject to Principal Component Analysis (PCA), and from different principal component (PC) values were obtained for each imaged chromosome locus. To select the PC that corresponds to A/B compartments for each cell type, we calculated the Spearman correlation coefficients between the PC values and the corresponding locus CpG density for the first three PCs. The signs of PC values for each chromosome were then assigned based on their signs of correlation with the corresponding chromatin accessibility. We picked the PC whose values had the highest correlation with CpG density as the PC that corresponds to A/B compartments. Imaged loci whose selected PC values that were greater than 0 were assigned as compartment-A loci, whereas loci whose selected PC values that were equal to or less than 0 were assigned as compartment-B loci.

After we determined the A/B compartments for each genomic locus in major cell types, we assigned the A/B compartments for different cell types at subclass level, by directly transferring the A/B compartment identities to the subclass from the major cell type where the subclass belongs to (e.g., from excitatory neurons to L2/3 IT). We determined A/B compartment boundaries at the major class level because some subclasses have too few cells for accurate compartment boundary determination. Hence, in this work, we only considered compartment identity switching for genomic loci between major cell classes, and ignored such switching between subclasses within the same class, if such switching exists.

A/B segregation using pairwise spatial distances from DNA-MERFISH data

For each chromosome and cell type, we separated the locus pairs within the chromosome into A-A, B-B, and A-B based on the A/B compartment identity of the involved genomic locus. The A/B compartment identities were determined as described in the "A/B compartment analysis from DNA-MERFISH data" section above. The A/B segregation score was defined as follows using the spatial distances of A-A (and/or B-B) vs. A-B locus pairs from the cis-chromosomal pairwise distance matrices.

For each cell type, we normalized its cis-chromosomal pairwise distance matrices to remove the

effect from the difference in chromosomal territorial size between cell types as described the "Megadomain insulation strength of chromosomes exhibiting near-bipartite megadomains" section above. Because we observed bigger differences in A-A and B-B vs. A-B spatial distances at the short genomic distance range up to ~10-20Mb (fig. S11, A and B), We only considered locus pairs whose genomic distances were <10Mb for this A/B segregation score calculation. To control for the effect of genomic distances on spatial distances, we fitted the spatial-distance-vs-genomic-distance scattered plot for locus pairs in each cell type by a 2nd order polynomial function. This generated the expected spatial distance for any given genomic distance. Next, we calculated the normalized spatial distances by dividing the observed spatial distances by the expected spatial distances. The A/B segregation score was then defined as follows using the normalized spatial distances of A-A, B-B and A-B locus pairs:

$$A/B\ segregation = \frac{median\ of\ normalized\ spatial\ distances\ of\ A-B\ locus\ pairs}{median\ of\ normalized\ \ spatial\ distances\ of\ A-A\ and\ B-B\ locus\ pairs}$$

Differentially expressed (DE) gene locus selection for local A/B environment analysis

To select DE gene loci for the indicated cell type as shown in Fig. 4E, we used the normalized expression matrices from the "snRNA-seq data analysis of wild-type mouse brains" section above to find DE genes. For each given cell type (termed "reference cell type"), we performed "rank_genes_groups" analysis using the Scanpy package (*121*) to select the top 200 upregulated and top 200 downregulated DE genes relative to the rest of the cell using the Wilcoxon test. Next, we picked DE genes whose TSS are within 100kb of any of our imaged loci, and such loci that had at least one nearby DE gene were identified as DE gene loci.

Super-enhancer locus selection for local A/B environment analysis

The selection of super-enhancer loci for indicated cell types, as shown in Fig. 4F, was based on super-enhancer locus panel design as described in the "Genomic locus selection for DNA-MERFISH" section above. For each given cell type (termed "reference cell type"), we selected the candidate super-enhancer loci that were specific to the given reference cell type as the super-enhancer loci for this analysis. In the meantime, we randomly selected 100 loci from our super-enhancer panel that were specific to the other cell types but not to the reference cell type as the randomized super-enhancer control loci.

Local A/B density ratio analysis

To calculate the local density ratio of compartment-A and compartment-B loci for each imaged locus as in Fig. 4, D to F, we first calculated the local density score for each locus in single cells as follows. The local trans-A density score for any given locus was defined as the sum of the Gaussian probability density function values from all compartment-A loci from other chromosomes, where σ was the standard deviation for the Gaussian probability density function and was set to be 0.5 µm, x_r, y_r, z_r were the 3D coordinates (µm) of the given locus, and x_i, y_i, z_i were the 3D coordinates (µm) of any other A locus.

$$Density\ score(A) = \sum_{i=1}^{N} exp\left(-0.5\ \times (\frac{(x_i - x_r)^2}{\sigma^2} + \frac{(y_i - y_r)^2}{\sigma^2} + \frac{(z_i - z_r)^2}{\sigma^2}\right)$$

The local trans-B density scores were defined similarly.

Local A/B density ratio for any given locus was defined as the ratio between local trans-A density score and local trans-B density score and we then log2-transformed this ratio. After we obtained these local A/B density ratios in single cells, we then aggregated ratios from single cells that had >=600 decoded loci (to filter out single cell measurements that were less accurate) for each cell type. The cell-type median values for each locus were then calculated and used for the following analyses. In this study, we defined local A/B density based on trans-A and trans-B density scores (i.e., contributions from other chromosomes) because they are not confounded by their genomic distance to the given loci unlike the loci from the same chromosome.

To study the change in A/B density ratio between cell types as shown in Fig. 4, E and F, we additionally performed linear regression between the density ratios from all targeted genomic loci for any given cell type and those for a common reference cell type (L2/3 IT in this study). Based on the linear regression results, for any given cell type, we then normalized its A/B density ratios by this linear regression results to correct for the intrinsic differences in A/B density ratios between cell types. Another reason for this normalization is because the DE genes and super-enhancers were also derived from normalized snRNA-seq and snATAC-seq count matrices, where cell-type-intrinsic differences in total transcription and chromatin accessibility had been normalized.

Finally, for each given cell-type-specific DE gene or super-enhancer locus, we subtracted its local A/B density ratios in other cell types from that in the cell type where the given locus was identified (see the "DE gene locus selection for local A/B environment analysis" section and "Super-enhancer locus selection for local A/B environment analysis" section above for details on how these loci were identified). We then divided the local A/B density ratio difference (calculated from the above-mentioned subtraction) by the local A/B density ratio in the cell type where the given locus was identified. This generated the percentage changes in the local A/B density ratio. Next, for each reference cell type, the median of all A/B compartment density ratio percentage changes from all of its cell-type-specific DE gene (or super-enhancer) loci were shown in Fig. 4, E and F.

Radial positioning analysis for imaged genomic loci from DNA-MERFISH data

To calculate the nuclear radial positions for individual imaged genomic loci in single cells, we first generated the minimal 3D convex hull surface surrounding all decoded chromosome loci in a given cell. Then for each DNA locus, we calculated its spatial distance to the centroid of the 3D convex hull and normalized this distance to a corresponding radius of the 3D convex hull to obtain the normalized radial position of the locus. Specifically, the line connecting the centroid and the locus of interest was drawn and its intersection with the convex hull surface was determined. The normalized nuclear radial position was calculated by normalizing the locus' distance to the nuclear centroid over the distance between the nuclear centroid and the intersection point on the convex hull.

In addition, the cell-type median of its normalized nuclear radial positions was calculated from aggregated single-cell measurements, which were shown or used in Fig. 5, A to D; figs. S13 to S17. Only cells that had >=600 decoded loci were aggregated (to filter out single cell measurements that were less accurate) for each cell type. For each locus in each cell type, the standard deviation (SD) and 25th-75th percentiles of the single-cell measurement were calculated, as shown in fig. S15.

After obtaining the cell-type median of nuclear radial position for each target genomic locus, we examined the relationship between transcription activity, chromatin accessibility, and local A/B density ratio with the nuclear radial position of the genomic loci as shown in Fig. 5, A to D; fig. S16. We additionally grouped each locus into 5 different bins (every 20th percentile of the distribution of radial position across all imaged loci) for each cell type. The top and bottom 20th percentile were defined as the loci near the nuclear periphery and loci near the nuclear interior/center, respectively, for each cell type. Features such as the status of long gene expression (see the "Long gene expression analysis" section below for details) were assessed for loci near the nuclear periphery or near the nuclear interior/center as shown in fig. S17.

Long gene expression analysis

To examine whether a given locus contained a long gene (>300kb) that was highly expressed, as in fig. S17, we defined an active transcription cutoff value as the 90th percentile of the distribution of mean transcription level per cell across all genes from snRNA-seq data (*59*). Genes whose mean transcription level per cell is above this cutoff value were considered as highly active.

To characterize the associated function of long genes that are highly expressed in fig. S17, we further identify highly active long genes that were also located near the nuclear periphery in neurons. Next, we performed Gene Ontology analysis (g:GOSt analysis) using the online g:Profiler tool (https://biit.cs.ut.ee/gprofiler/gost) of these genes for each sub-ontologies (*126*). Results of g:GOSt analysis were downloaded from the online g:Profiler tool and re-plotted in fig. S17.

Estimation of histone mark levels for each imaged DNA locus from sequencing data

To approximate cell-type-specific histone mark levels for each imaged DNA locus as used in figs. S16C and S18, we similarly created bins centered around each imaged loci and summed the reads of the corresponding histone mark for each bin as described in the "Estimation of transcriptional activity and chromatin accessibility for each imaged DNA locus from sequencing data" section above. The cell-type-resolved single cell histone profiles (H3K9me3, H3K27ac, H3K4me3) were obtained from the Paired-Tag data from Ref. (*76*).

Analysis of transcriptional regulation by MeCP2 from single-cell RNA sequencing data

For analysis of the relationship between radial positioning and transcriptional regulation by MeCP2 for cell types in the cortex, single-cell RNA sequencing results from the visual cortices of WT *Mecp2 +/y* and mutant *Mecp2 -/y* mice were downloaded from GEO (GSE113673) (*83*). We used similar normalization, dimensionality reduction, and clustering analysis as described in the "Cell clustering analysis of RNA-MERFISH data" section. We used marker genes *Camk2a*,

Olig1, *Cx3cr1* and *Cldn5* to identify clusters corresponding to excitatory neurons, oligodendrocytes and OPCs, microglia, and vascular cells, respectively.

For DE gene analysis, raw count matrix was used, followed by per-cell RNA count normalization and log transformation. Differential expression (DE) scores calculated from "scanpy.tl.rank_genes_groups" were used for analysis in Fig. 5E, Fig. 6F, fig. S19, fig. S26G and fig. S27E to circumvent the effect from lowly expressed genes. Specifically, the DE score of a gene is the t-statistics score when comparing the expression-level distributions of WT and KO cells using Welch's t test.

The nuclear radial positioning for each gene was estimated by using the cell-type median of the normalized nuclear radial position of its closest imaged locus, in a cell-type-specific manner (obtained from the WT dataset, see the "Radial position analysis for imaged genomic loci from DNA-MERFISH data" section above). However, if the genomic distance between the closest imaged locus and the gene is greater than 3Mb, we excluded the gene from further analysis. For the analysis of the relationship between nuclear radial positioning and transcriptional regulation by MeCP2, we divided the genes into ten equal bins based on their normalized nuclear radial position. The average and 95% confidence interval of transcription level changes from DE gene analysis as a function of the normalized nuclear radial position were then plotted for each bin in Fig. 5E, Fig. 6F, fig. S19, fig. S26G and fig. S27E.

<u>Analysis of transcriptional regulation by MeCP2 from bulk RNA sequencing data</u>

Published bulk RNA sequencing data from *Mecp2* deletion or mutations were downloaded from GEO (GSE128186 (*85*), GSE152800 (*86*) and GSE139033 (*50*)). For all three datasets, genes that have at least one count per million reads across all samples in a given comparison were used for further DE gene analysis. For the dataset from Ref. (*86*), the WT and MM2 mutations from two age groups were combined for analysis. Pydeseq2 (*127*) with default settings was used to perform DE gene analysis. For datasets from Ref. (*85*) and Ref. (*86*), because they contain bulk RNA sequencing from forebrain and hypothalamus tissues, respectively, the nuclear radial position of each gene was estimated by the median of normalized nuclear radial positions of the gene's closest imaged locus (with a 3Mb threshold) for all cell types (Fig. 5, G and H) (obtained from the WT dataset, see the "Radial positioning for imaged genomic loci from DNA-MERFISH" section above). For the dataset from GSE139033 (*50*), because Ngn2-induced neurons are excitatory neurons (*128*), the nuclear radial position of each gene was estimated by the median of normalized nuclear radial positions of the gene's closest imaged locus (with a 3Mb threshold) for excitatory neurons only (Fig. 5I). Genes were binned into ten equal bins based on their normalized nuclear radial positions similar to the "Analysis of transcriptional regulation by MeCP2 from single-cell RNA sequencing data" section above.

<u>Identification of WT and *Mecp2* KO cells from *Mecp2* +/- female mice</u>

The mean intensity of MeCP2 immunofluorescence signals within each segmented cell was first calculated and $\log_{10}$ transformed. Then, the mean MeCP2 intensity of a given cell was normalized by the mean intensity across all cells belonging to the same cell type and imaged during the same experiment. Such a normalized mean MeCP2 intensity helped to adjust the differential MeCP2 levels among different cell types (*129*) and also adjust the batch effects. Local relative MeCP2 signal was calculated as the $\log_2$ ratio of the mean MeCP2 intensity within

each segmented cell divided by the mean MeCP2 intensity of the background surrounding the cells. The background region of interest was defined as a ring area with 10-pixel thickness around the segmented cell. Cells with normalized MeCP2 mean intensity less than 0.92 and local MeCP2 signal less than 0.2 were defined as KO cells and cells with normalized MeCP2 mean intensity greater than 0.98 and local MeCP2 signal greater than 0.5 were defined as WT cells (see fig. S20A). The rest of the cells (~20%) remained un-determined and were not used in further analyses.

Analysis of MeCP2's effect on A/B compartment

Due to the lower cell number across cell types from the *Mecp2+/-* mice as compared to those from wildtype mice, we found that A/B identification using "A/B compartment analysis from DNA-MERFISH data" generated noisier cross-correlation matrices in this dataset especially for cell types with low cell numbers. To utilize more information obtained through DNA-MERFISH measurements, we utilized the median of trans-chromosomal distances between all trans-chromosomal loci pairs. The underlying assumption is that loci belonging to the same compartment should have similar profiles of trans-chromosomal distances. First, we generated a trans-distance matrix. Each pixel in the matrix corresponds to the median distances of trans-chromosomal loci pairs, including loci pairs from homologous chromosomes. ChrX was excluded from this analysis because of the large difference between Xa and Xi chromosomes. Second, to normalize any effect from radial positions, we divided our trans-distance matrix into submatrices, each representing trans-distances between any two chromosome pairs (including homologous chromosomes) and normalize each submatrix by the row mean. Then, for each loci pair, we calculated the similarity score based on the Euclidean distance between their normalized trans-median distances to all other loci to generate a similarity matrix for trans distances. The similarity score was defined as the negative of the Euclidean distance and then mapped to the range between 0 and 1. Loci pairs with smaller Euclidean distances between their normalized trans-median distances are similar in terms of their spatial relationship to all other loci and thus have a high similarity score. On the genome-scale, this similarity matrix showing the similarity scores of trans-chromosomal distances also displayed a plaid pattern like the one observed in cis-chromosome cross correlation matrices. We conducted PCA on the similarity matrix and the values from PC1 turned out to always have the highest correlation with CpG density than other PCs and we used PC1 to determine A/B compartment identities. The A/B identities were identified using a threshold of 0. Thus, loci with positive PC1 values assigned to compartment A and loci with negative PC1 values were assigned to compartment B loci. These compartment analyses were used to generate Fig. 6D-F, figs. S24, S26 and S27. Compartment analysis using cis-chromosome matrices as described above generated noisier but similar results.

Analysis of MeCP2's effect on chromatin organization

The nuclear volume of cells in *Mecp*2+/- mice was estimated as described in the "Estimation of the nuclear volume from DNA-MERFISH" section above (fig. S21). For unnormalized distance to the nucleus center, we calculated the distance of each genomic locus to the nuclear center by generating the minimal 3D convex hull surface of all decoded loci in each cell and calculating the Euclidean distance between the locus of interest and the centroid of the 3D convex hull (fig. S25A). The normalized nuclear radial position was calculated in the same manner as described in

the “Radial position analysis for imaged genomic loci from DNA-MERFISH data” section above (Figs. 5 and 6; figs. S19, S24, S25, S26, and S27). After A/B compartment identity calculation (see the “Analysis of MeCP2’s effect on A/B compartment” section above), local A/B density ratio changes upon *Mecp2* deletion were calculated as described in the “Local A/B density ratio analysis” section above (Fig. 6, D to F; figs. S24, S26, and S27). In the *Mecp2 +/-* visual cortex dataset, due to the lower cell numbers measured for the visual cortex, the A/B identities of loci were transferred from the *Mecp2 +/-* primary motor cortex datasets. For analysis of the radial-position dependence of the effect of *Mecp2* deletion, the genes or loci were grouped into 10 equal bins based on its cell-type-specific normalized radial positions calculated from wild-type cells (Figs. 5 and 6; figs. S19, S24, S25, S26 and S27).

Supplementary Figures

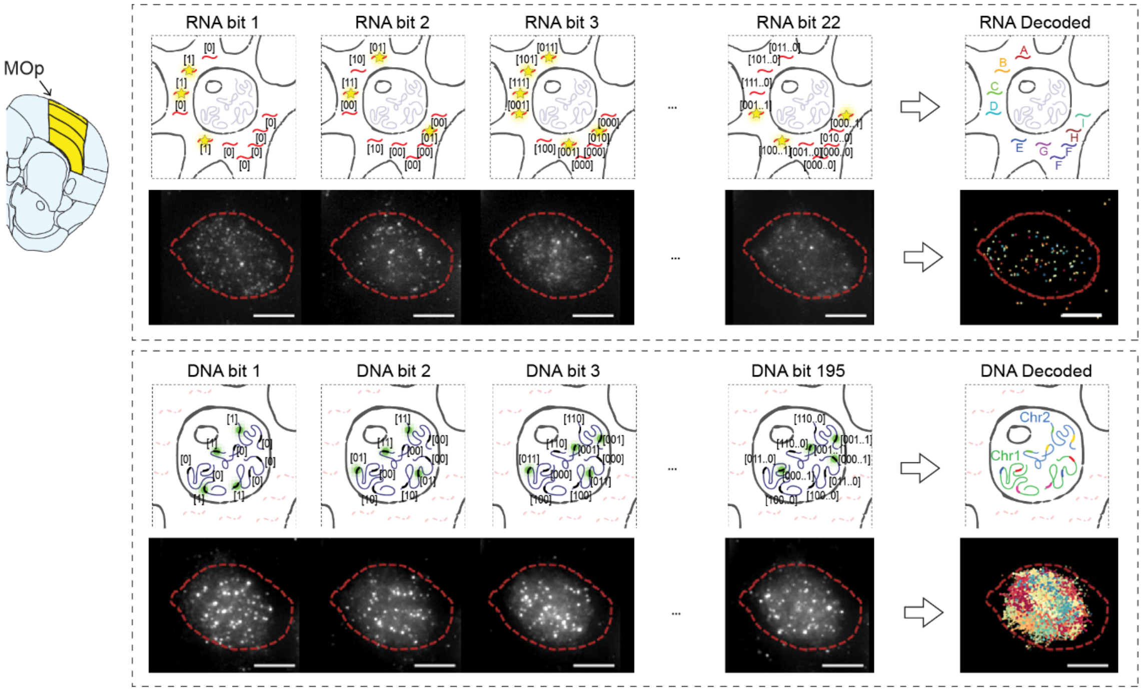

Fig. S1. Experimental scheme of integrated RNA- and DNA-MERFISH. Schematics of integrated RNA-MERFISH and DNA-MERFISH. Left: Schematic annotation of the mouse MOp according to the Allen Common Coordinate Framework version 3 (http://atlas.brain-map.org/) (***112***). Right, Top: Schematic of RNA-MERFISH (top) and per-bit RNA-MERFISH images of an example cell (bottom). Right, Bottom: Schematic of DNA-MERFISH (top) and per-pit DNA-MERFISH images of the same cell (bottom). Red dashed lines in the images indicate the area where RNA and DNA foci were visualized and decoded.

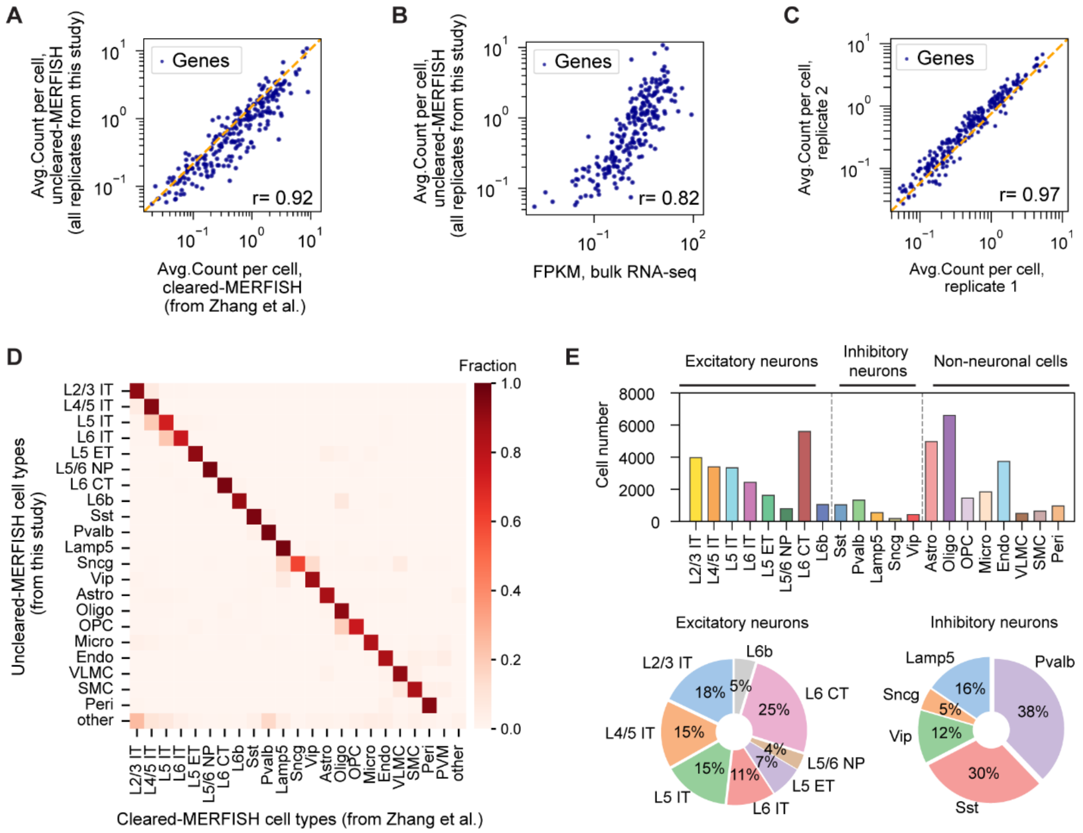

Fig. S2. Performance of the tissue-uncleared RNA-MERFISH protocol in resolving molecularly defined cell types in the mouse MOp. (A) Scatterplot of the average copy number per cell of individual genes determined by RNA-MERFISH from our modified protocol without tissue clearing versus copy number per cell of individual genes determined by RNA-MERFISH with tissue clearing (*62*). (B) Scatterplot of the average copy number per cell of individual genes determined by RNA-MERFISH from our modified protocol versus expression level of the same genes determined by bulk RNA-seq (*124*). (C) Scatterplot of the average copy number per cell of individual genes determined by RNA-MERFISH from our modified protocol between two biological replicates (13383 cells and 14733 cells respectively) as an example, out of all four technical replicates and three biological replicates as described in Fig. 1A. In (A to C), the Pearson correlation coefficients (r) are indicated in the plot. Yellow dashed lines indicate y=x. (D) Confusion matrix for correspondence between cell types determined by RNA-MERFISH in this study and cell types determined by a multi-layer perceptron (MLP) classifier trained by data from the previous RNA-MERFISH study with tissue clearing (*62*). Each element in the matrix represents the fraction of cells from the given cell type (x-axis) determined by the MLP classifier (Materials and Methods) that was assigned to the individual cell type determined by *de novo* clustering in this study (y-axis). (E) Cell-type composition of the MOp. Top: Bar plots show the cell numbers for each MERFISH-identified cell type, combined from all biological replicates in this study. Bottom: Pie charts show the fractions of excitatory neurons (left) and inhibitory neurons (right) belonging to the indicated neuronal cell types, combined from all biological replicates. Our current analysis includes a larger area of the white matter and hence has a slightly different cell-type composition compared to our previous MERFISH analysis of MOp, which is focused on the grey matter (*62*).

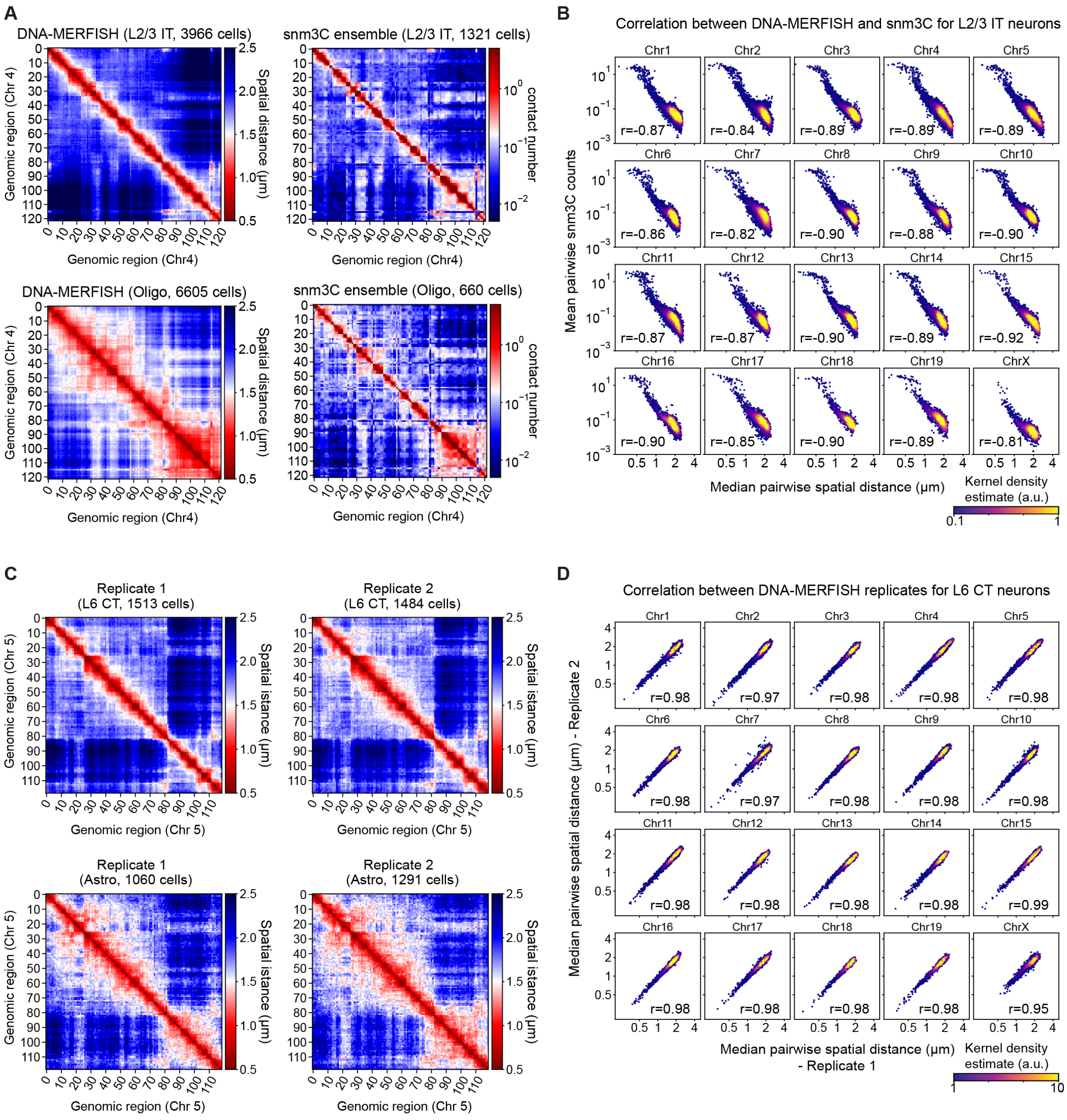

Fig. S3. Comparison of 3D-genome organizations determined by DNA-MERFISH and snm3C and between DNA-MERFISH experimental replicates. (A) 3D chromatin organization of an example chromosome (Chr4, Genomic regions 0-121 corresponding to 3.0Mb – 156.3Mb) revealed by DNA-MERFISH and snm3C-seq (*35*), respectively. Left matrices: Median cis-chromosomal pairwise distance matrices of Chr4 for L2/3 IT neurons (left, top) and oligodendrocytes (left, bottom), derived from DNA-MERFISH data. Right matrices: Ensemble cis-chromosomal pairwise contact matrices of Chr4 for L2/3 IT neurons (right, top) and oligodendrocytes (right, bottom), derived from snm3C-seq. (B) Scatterplots of the median cis-chromosomal pairwise distances derived from DNA-MERFISH data versus the mean contact counts from snm3C-seq data (*35*) for all imaged locus pairs in the indicated chromosomes in L2/3 IT neurons. The kernel density in the scatterplots estimates the two-dimensional distribution. (C) Similar to (A), but for 3D chromatin organization of an example chromosome

(Chr5, Genomic regions 0-118 corresponding to 3.7Mb – 151.2Mb) revealed by DNA-MERFISH from two biological replicates. (D) Similar to (B), but for scatterplots of the median cis-chromosomal pairwise distances derived from DNA-MERFISH data from two biological replicates for L6 CT neurons, as in (C). In (B) and (D), the Pearson correlation coefficients (r) are indicated.

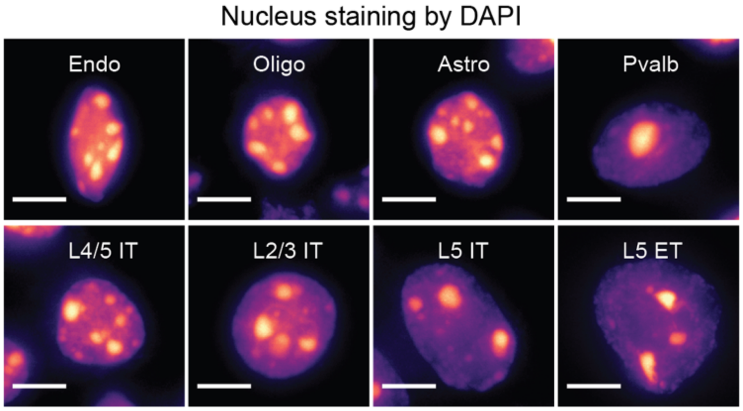

Fig. S4. Cell-type-dependent variations in the cell-nucleus sizes visualized by DAPI staining in mouse brains. Representative maximum-projection images of DAPI staining are shown for several cell types. Scale bar: 5µm.

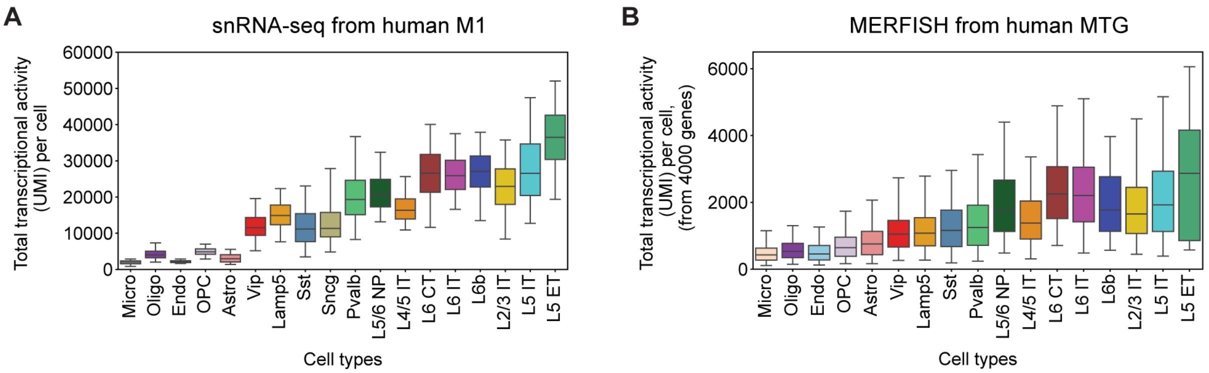

Fig. S5. Cell-type-dependent variations in transcriptional activity in the human brain. (A) Boxplot for the distribution of total transcriptional activity across individual cells in each cell type in the human primary motor (M1) cortex. Total transcriptional activity per cell was calculated using the sum of UMIs from snRNA-seq data of the human M1 (*70*). (B) Boxplot for the distribution of total transcriptional activity across individual cells in each cell type in the human middle superior temporal gyrus (MTG). Total transcriptional activity per cell was estimated using the total RNA count per cell from 4000 genes measured by RNA-MERFISH (*56*). Cell types were ordered as in Fig. 1, D and E. The center line, box, and whisker represent the median, the 25th-75th percentile, and the 5th-95th percentile, respectively.

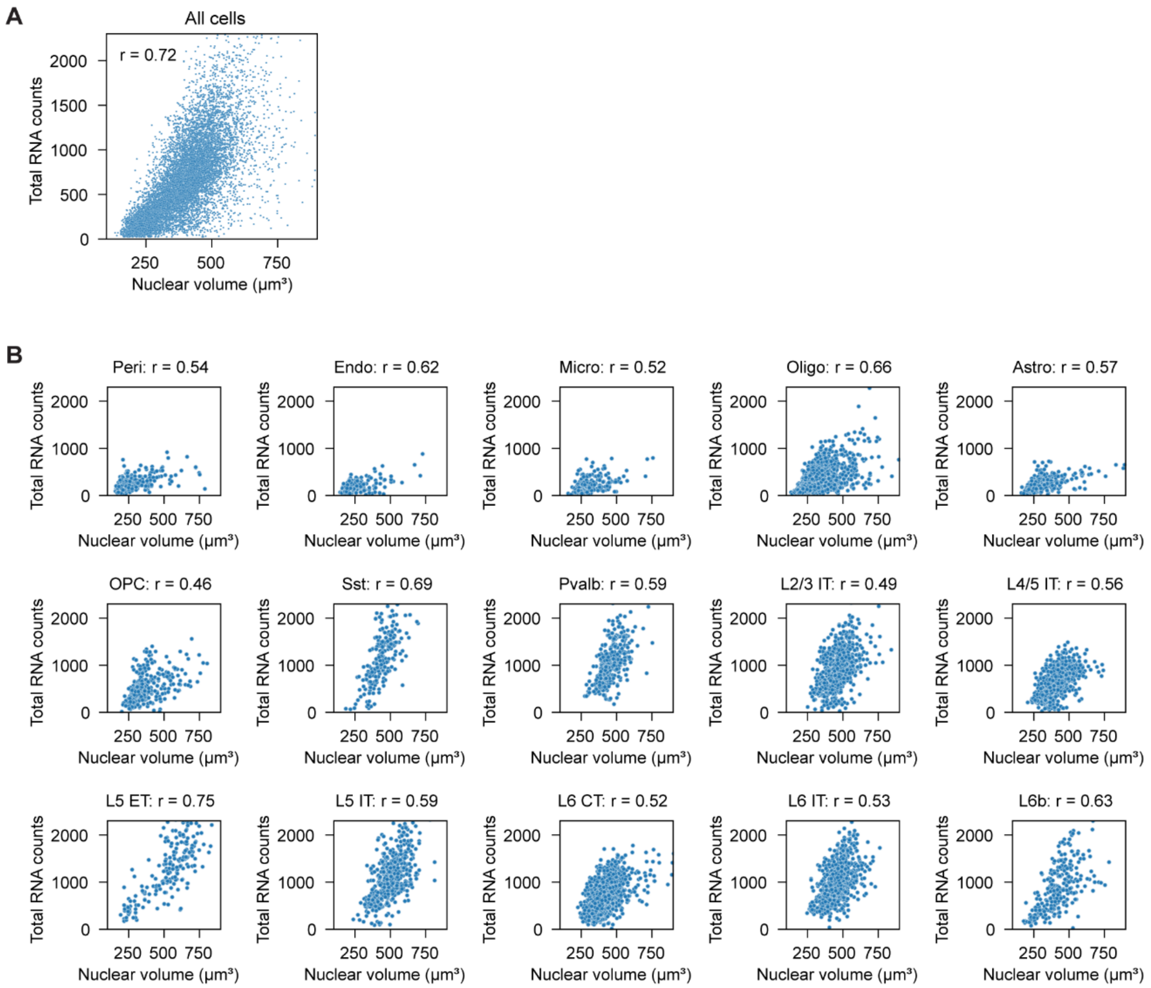

Fig. S6. Correlation between cell-nucleus size and transcriptional activity of individual cells measured by MERFISH. (A) Scatterplots of the cell-nucleus volume and total RNA counts from RNA-MERFISH of 242 genes across all single cells of all cell types. **(B)** Similar to (A), but for each indicated cell type. Each dot represents a single cell. The Pearson correlation coefficients (r) are indicated. We note that since we only measured 242 genes in RNA-MERFISH, the total transcriptional activity of single cell is only a crude estimation in the relative sense between cells, which might also contribute to the lower Pearson correlation coefficients observed for single cells here as compared to that observed for cell-type medians shown in Fig. 1F.

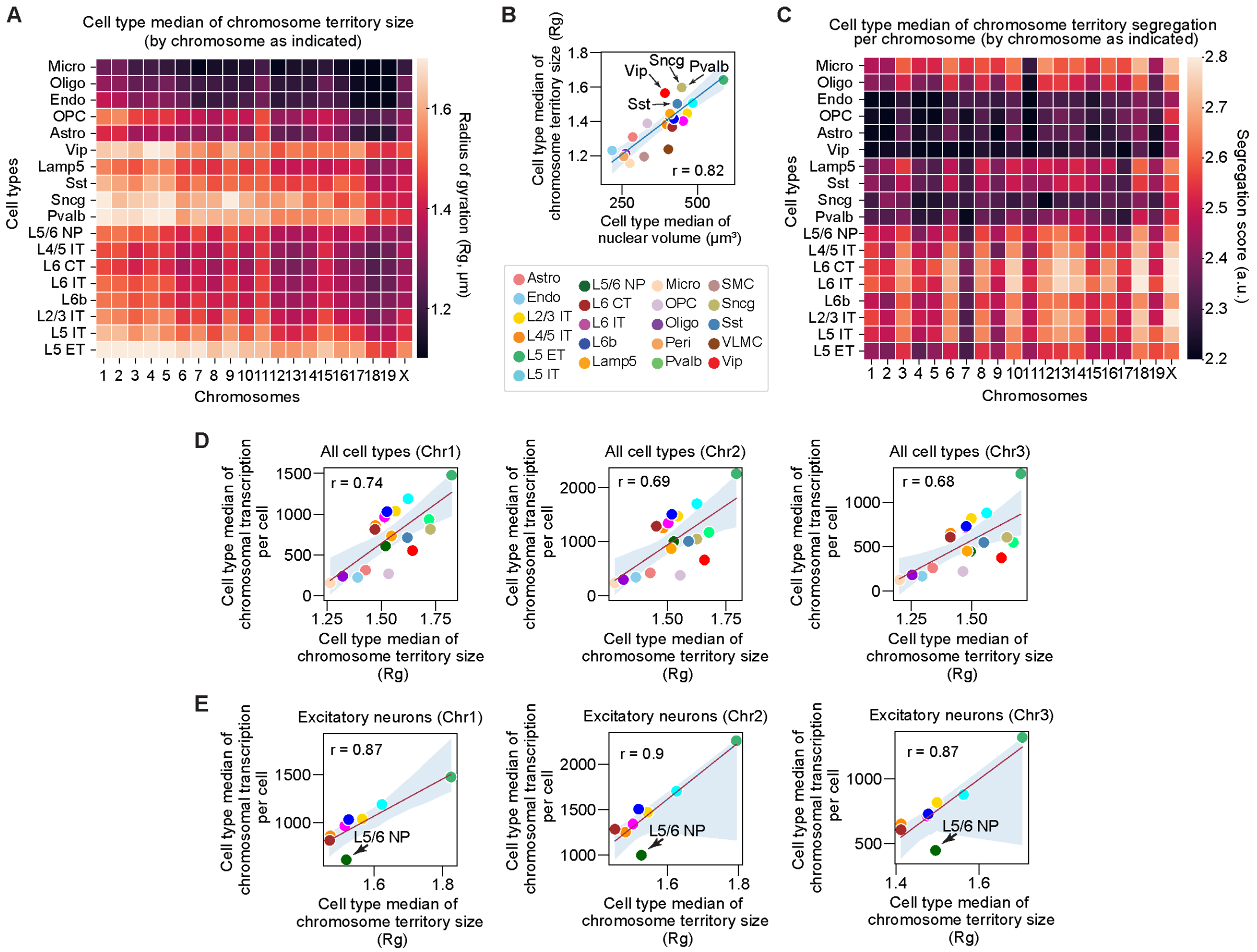

Fig. S7. Cell-type-dependent variations in chromosome-territory sizes and transcriptional activity. (A) Heatmap of the median chromosome-territory sizes across individual cells in each cell type. Each pixel in the heatmap represents the median radius of gyrations (Rg) for the indicated chromosome (columns) within the indicated cell type (rows). P<0.0001 for one-way ANOVA comparison of chromosomal territorial sizes across different cell types for Chr1-19, and ChrX. (B) Scatterplot of the cell-type medians of chromosome-territory sizes versus nuclear volume. The chromosome-territory size per cell is calculated by averaging over all chromosomes (Chr1-19 and ChrX). Arrowheads indicate several inhibitory neuronal types that have larger chromosome-territory sizes on average compared to excitatory neuronal types with similar nuclear volumes. (C) Heatmap of the mean segregation scores of chromosome territories across individual cells in all cell types. Each pixel in the heatmap represents the segregation score between the corresponding chromosome (columns) and all other chromosomes averaged over all cells in the indicated cell type (rows). Chromosome-territory segregation score was calculated based on the intermixing level between loci from the given chromosome and loci from other chromosomes (within the same cell) in 3D space (Materials and Methods). (D) Scatterplot of cell-type medians of total transcriptional activity for Chr1 (left), Chr2 (middle), and Chr3 (right) versus the cell-type medians of the radius of gyration of the same chromosome. Total transcriptional activity of the indicated chromosome for each cell was estimated using the sum of UMIs of genes in this chromosome from snRNA-seq data (*59*). (E) Similar to (D), but for excitatory neurons only. Arrowheads indicate the L5/6 NP neuron that deviates from the

correlation, which may be due to either the fewer cells of L5/6 NP neuron in this analysis or a real cell-type difference. For scatterplots in (B), (D), and (E), the line and the shade represent the fitted linear regression line and the 95% confidence interval, respectively. The Pearson correlation coefficients (r) are indicated.

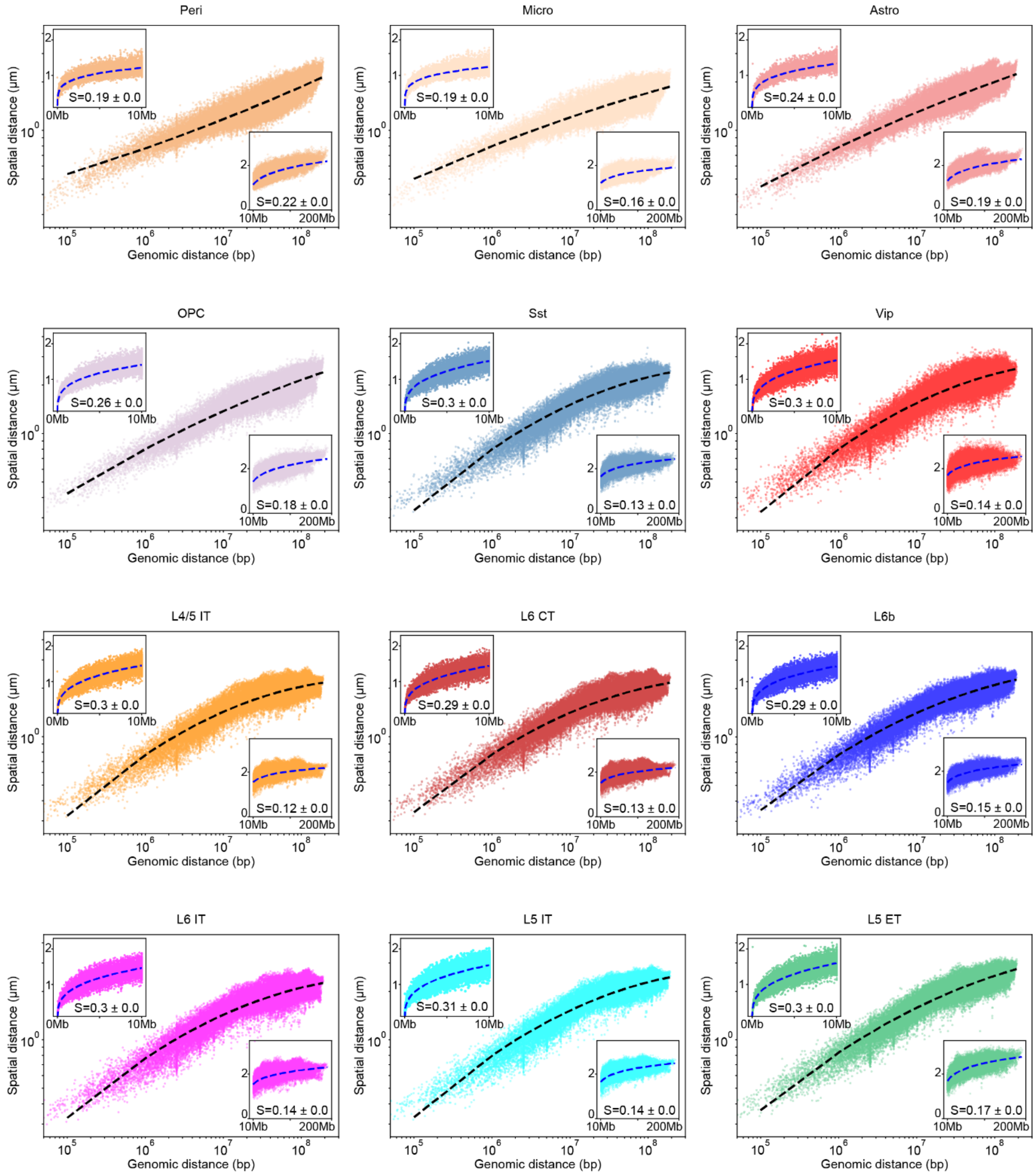

Fig. S8. Scaling between spatial distance and genomic distance of genomic locus pairs for different cell types. Scatterplots of spatial distances versus genomic distances for cis-chromosomal locus pairs from all chromosomes for indicated cell types. Dashed black lines in the main panels indicate the 2nd order polynomial function fit. In each scatterplot, the top left Inset shows spatial distances versus genomic distance scaling on linear scale at short genomic-distance range (<10Mb), shown together with a power-law fit (dashed blue lines). Bottom right inset: similar to top left inset, but for scaling at long genomic-distance range (>10Mb). Scaling power exponents (S) derived from the fits are indicated.

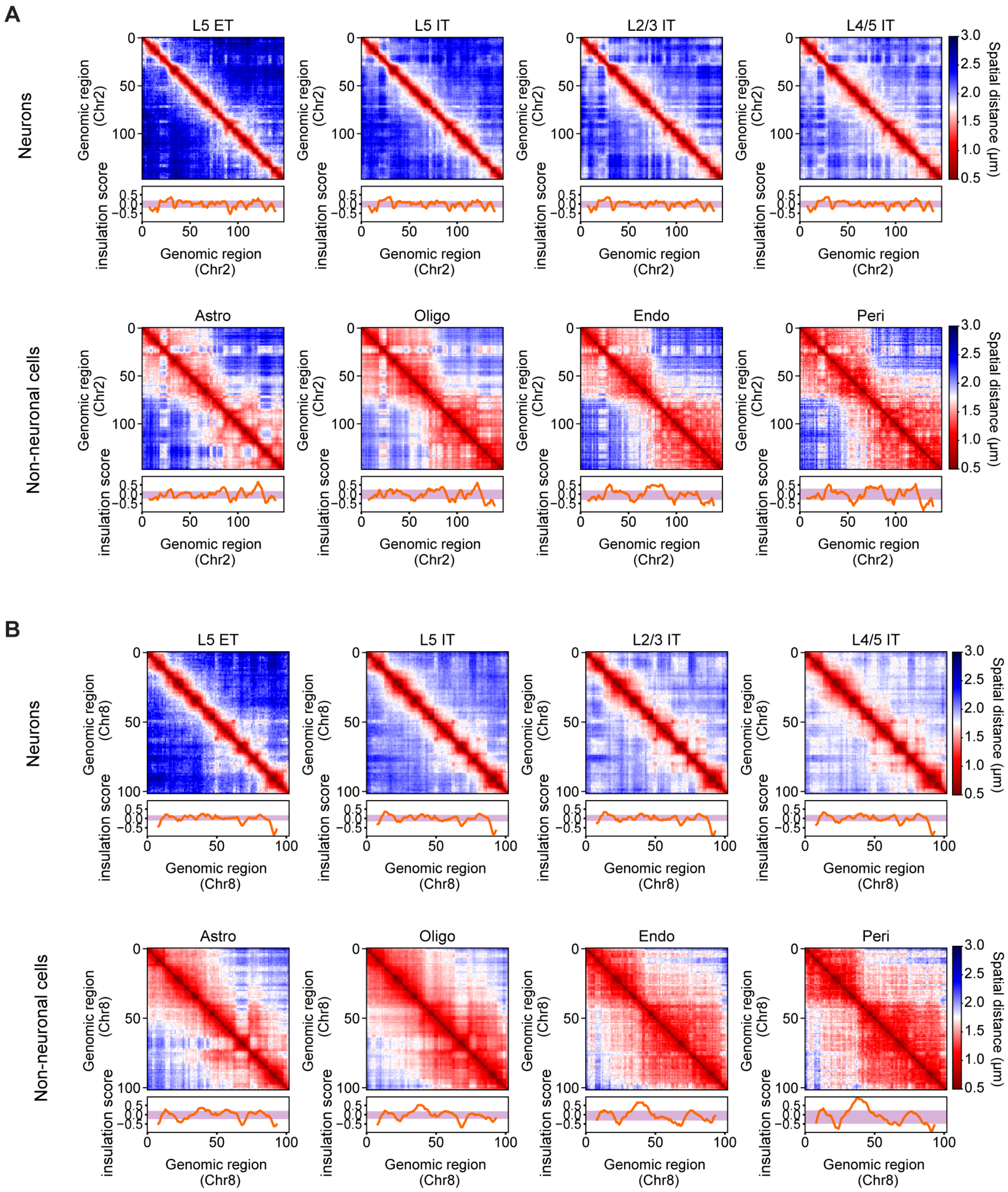

Fig. S9. Different prominence of megadomain-like structures among different cell types. (A) Similar to Fig. 3A, but for median cis-chromosomal pairwise distance matrices of Chr2 in indicated example cell types, with normalized insulation scores along the genomic coordinate shown below. Genomic regions 0-147 corresponding to 3.2Mb – 181.2Mb in Chr2. Top row is for neurons and bottom row is for non-neuronal cells. Purple shades represent the interquartile ranges (IQRs, 25th-75th percentiles) of insulation scores. (B) Similar to (A), but for Chr8. Genomic regions 0-101 corresponding to 3.7Mb – 128.7Mb in Chr8.

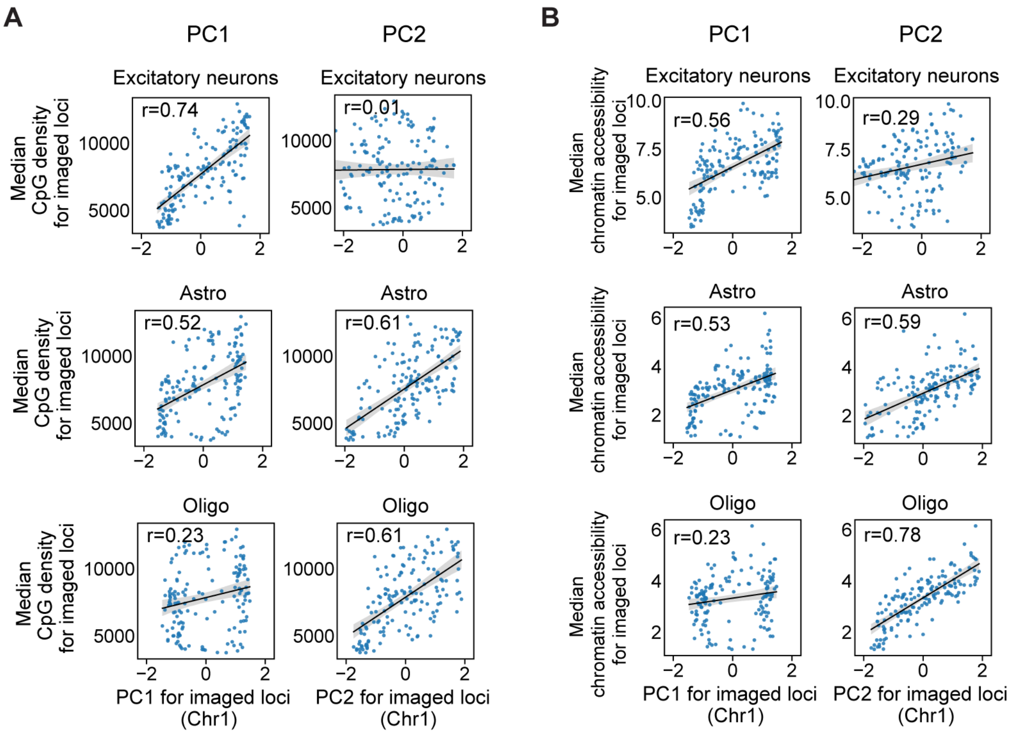

Fig. S10. Scatterplots for the PC values of the cross-correlation matrix versus the CpG density or the cell-type median of chromatin accessibility of Chr1 for several major cell types. (A) for CpG density (B) for chromatin accessibility. For each locus, the cell-type median of chromatin accessibility was calculated using snATAC-seq data (*59*) (Materials and Methods) and the PC1 or PC2 values were calculated from the cross-correlation matrices as shown on Fig. 3C. The Spearman correlation coefficients (r) are indicated in each plot.

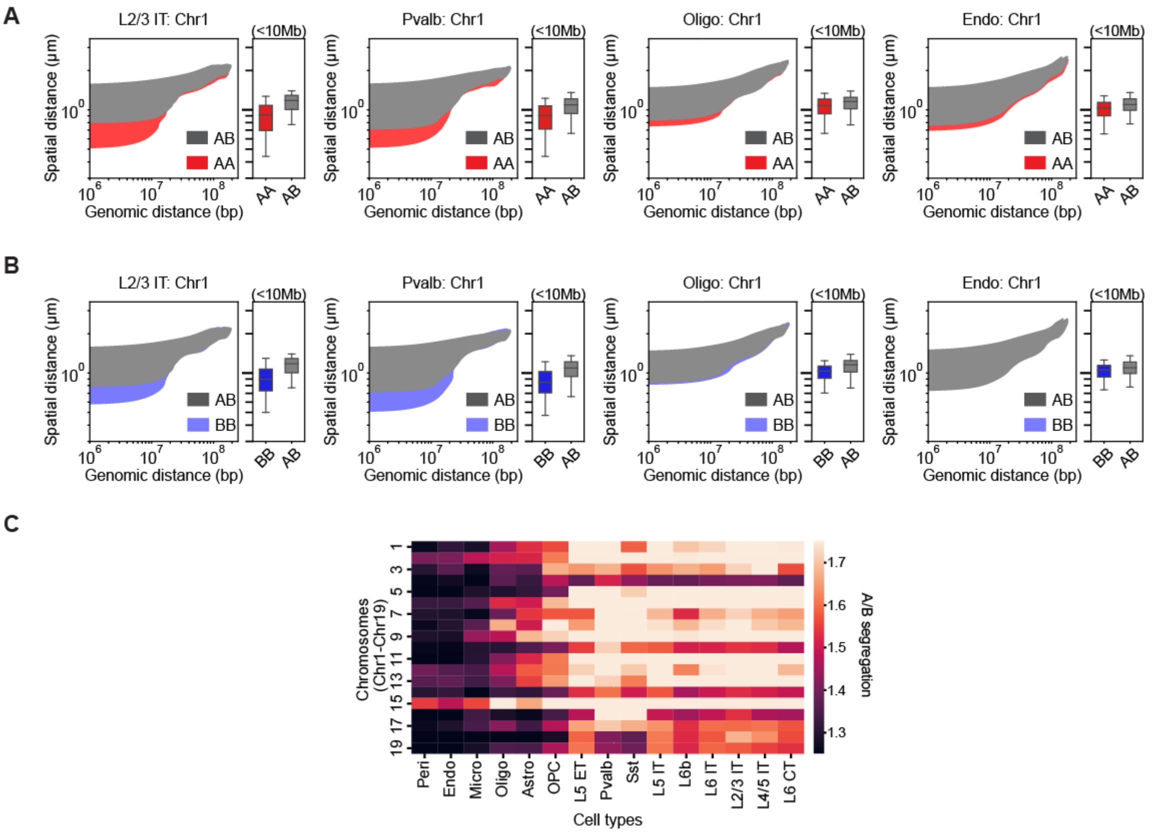

Fig. S11. Quantification of A/B compartment segregation score shows that neurons tend to exhibit stronger A/B segregation than non-neuronal cells. (A) 2D distributions (by kernel density estimation, showing 5th percentile in kernel density) of spatial distances and genomic distances of A-A (red) and A-B (grey) locus pairs in Chr1, with boxplots on the right showing the distributions of spatial distances of A-A (red) and A-B (grey) locus pairs with genomic distances <10Mb. In the boxplots, the center line, box, and whisker represent the median, 25th-75th percentile, and 5th-95th percentile, respectively. (B) Similar to (A), but 2D distributions of spatial distances and genomic distances of B-B (blue) and A-B (grey) locus pairs in Chr1. (C) Heatmap showing the quantification of A/B segregation scores for different chromosomes across different cell types. A/B segregation scores were defined as the ratio of the median of normalized spatial distances of A-B locus pairs (<10Mb) to the median of normalized spatial distances of A-A and B-B locus pairs (<10Mb) (Materials and Methods).

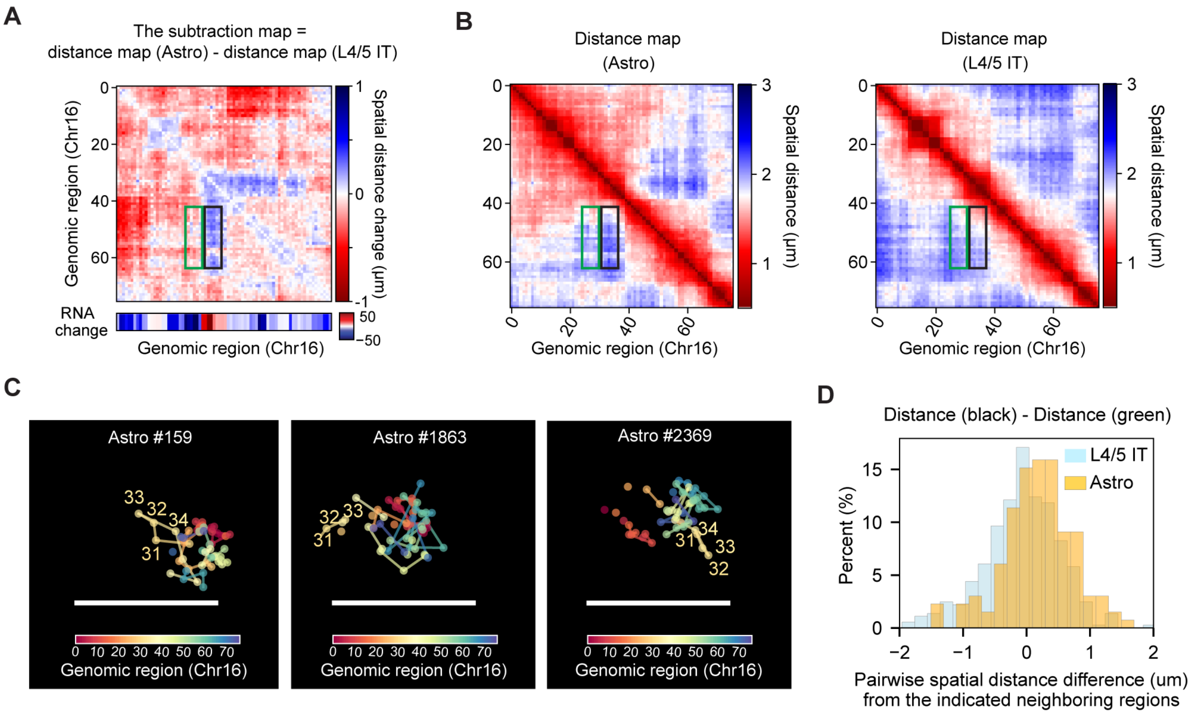

Fig. S12. "Looping-out" structure of highly transcribed genomic regions in chromosomes in cell types with low overall transcriptional activity. (A) Matrix representing the change in median cis-chromosomal pairwise distance from L4/5 IT to astrocytes for Chr16. Heatmap below the matrix shows changes in transcript counts of the corresponding genomic loci from L4/5 IT to astrocytes (derived from snRNA-seq data (*59*)). Genomic regions 0-75 corresponding to 3.7Mb – 97.7Mb in Chr16. The Black box marks locus pairs between loci 30-37 and loci 43-63. Green box marks locus pairs between loci 22-29 and loci 43-63. (B) Corresponding median pairwise distance matrices shown for astrocytes and L4/5 IT separately. (C) Examples of single chromatin traces showing the "looping out" of loci 31-34 (within the black boxed region) in astrocytes. Scale bars: 5µm. (D) Histograms of differences between the average distance between loci 31-34 and loci 43-63 within the black boxed region and the average distance between loci 26-29 with loci 43-63 within the green boxed region in astrocytes (orange) and L4/5 IT neurons (light blue).

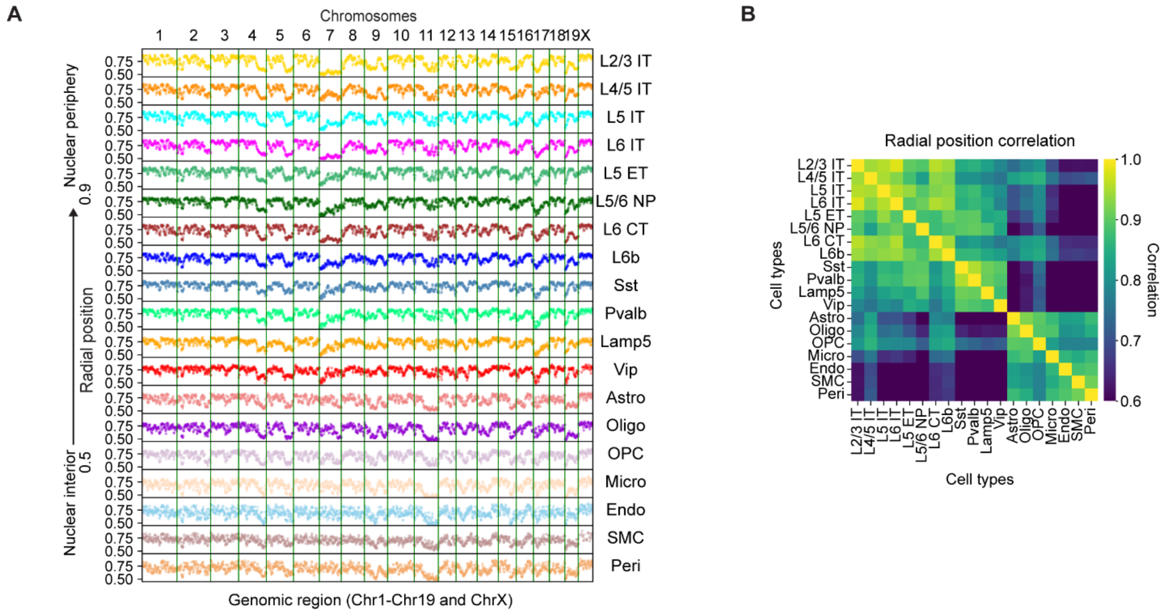

Fig. S13. Nuclear radial positions of genomic loci across different cell types. (A) Cell-type median nuclear radial positions of all imaged loci (across Chr1-19 and ChrX, each column representing one chromosome) for individual cell types (rows). For each locus, nuclear radial position was calculated as the normalized distance of the locus to the centroid of the nucleus, with the maximum locus-to-centroid distance along the same direction normalized to 1. (B) Correlation matrix for nuclear radial positions of genomic loci between cell-type pairs. Each pixel in the heatmap represents the Pearson correlation coefficient of the normalized radial positions of all imaged chromosome loci between an indicated cell-type pair.

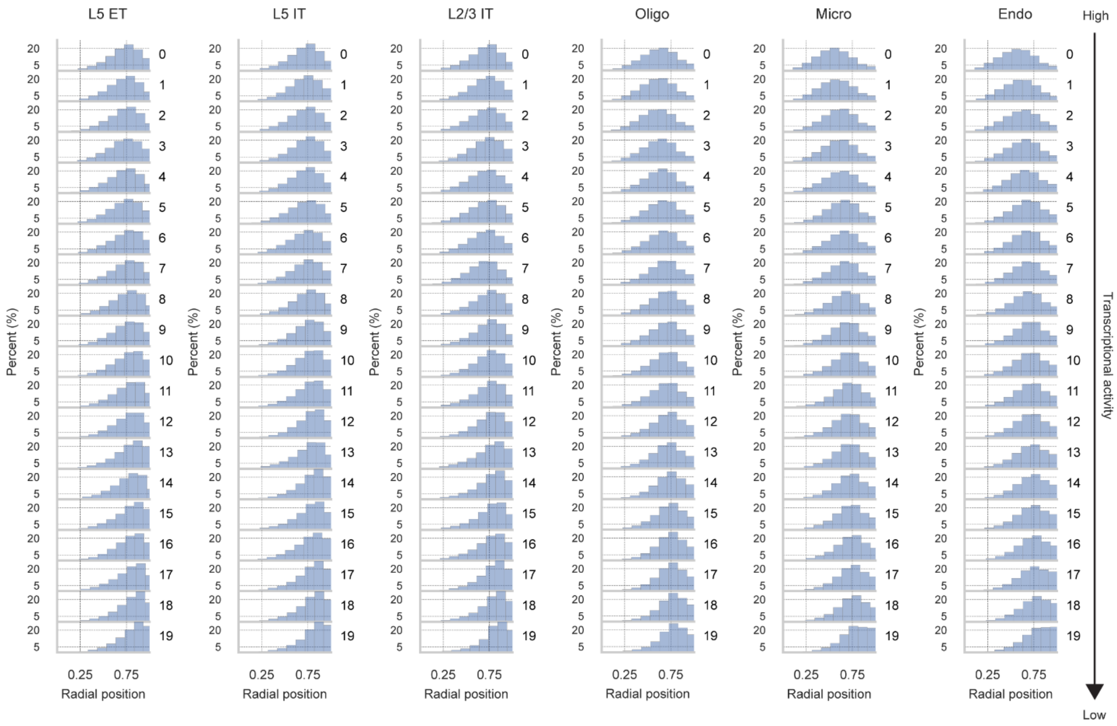

Fig. S14. Histogram of the distribution of nuclear radial positioning of different groups of imaged loci with different transcriptional activity in indicated cell types. Twenty different groups of imaged loci, from top to bottom, are ranked based on their median transcriptional activity for each cell type.

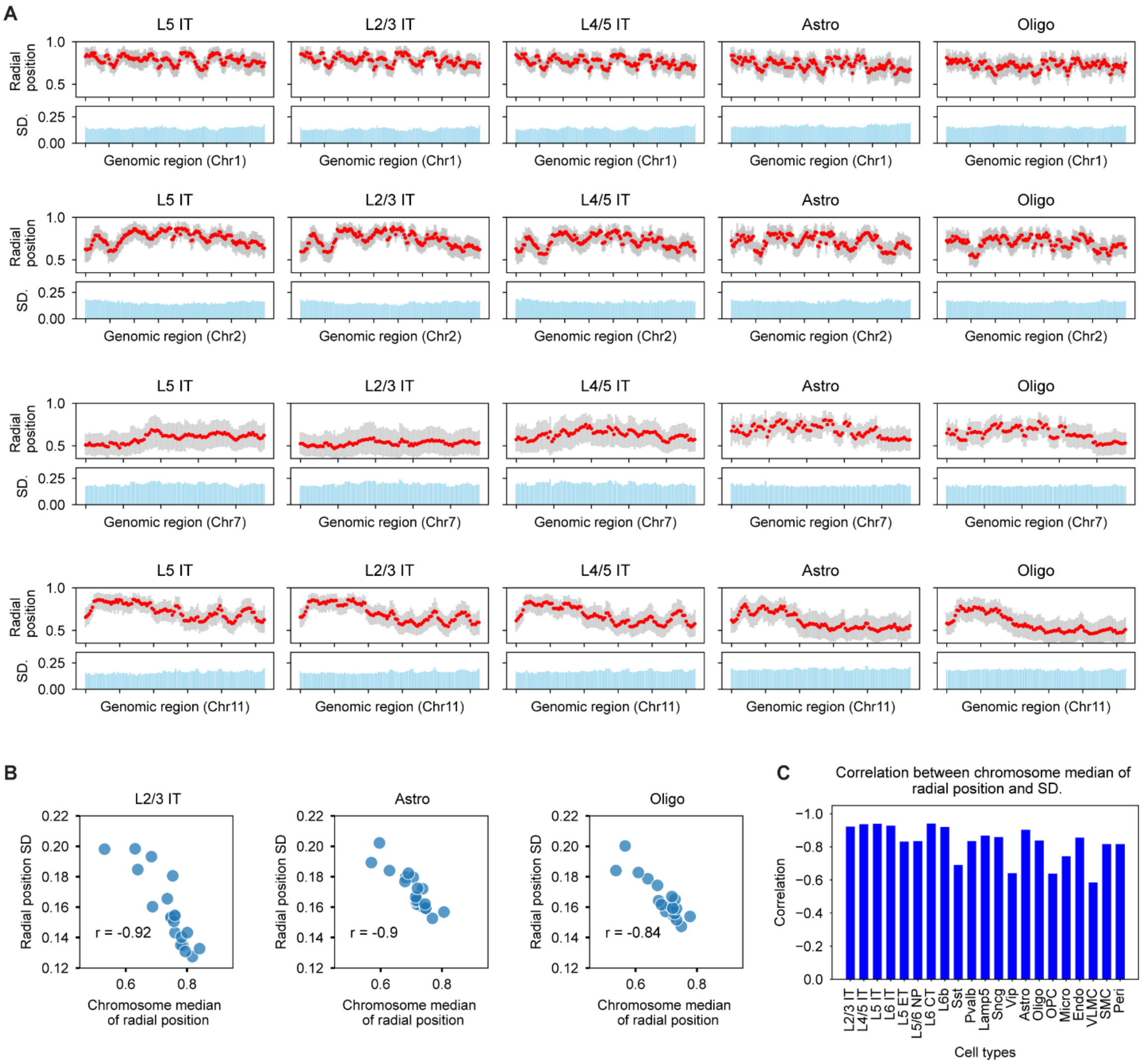

Fig. S15. Relationship of nuclear radial positions and cell-to-cell variations of radial positions. (A) Single-cell measurement of radial position of imaged loci from several chromosome examples (Chr1, 2, 7, and 11) for indicated cell types. Top: Radial position distributions across individual cells. Red dots are cell-type medians and gray lines are cell-to-cell variability measured by 25-75% confidence intervals; Bottom: standard deviation (SD) values of the radial position distributions. (B) Scatterplots of radial-position medians and radial-position standard deviations across chromosomes (Chr1-19, and ChrX) for indicated cell types. For each chromosome, both radial position median and radial position SD are the median values of the corresponding metrics for all loci in the chromosome. Each symbol corresponds to one of the 20 chromosomes (Chr1-19, and ChrX). The Spearman correlation coefficients (r) are indicated in each plot. (C) Bar plots showing the correlation values derived from (B) for all cell types.

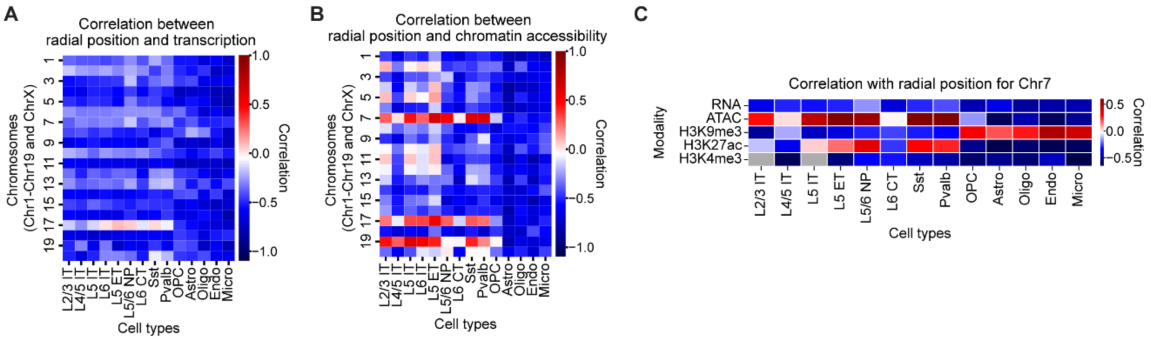

Fig. S16. Relationship between nuclear radial positioning and various chromatin properties across different cell types for different chromosomes. (A) The Spearman correlation between cell-type medians of normalized radial positions and transcriptional activity (calculated from snRNA-seq data (*59*)) of all loci from the indicated chromosome (y-axis) in the indicated cell type (x-axis). (B) The spearman correlation between cell-type medians of normalized radial positions and chromatin accessibility (calculated from snATAC-seq data (*59*)) of all loci from the indicated chromosome (y-axis) in the indicated cell type (x-axis). (C) The spearman correlation between cell-type medians of normalized nuclear radial positions and cell-type medians of various quantities (transcription level (RNA), chromatin accessibility (ATAC), H3K9me3 level, H3K27ac level, and H3K4me3 level) for genomic loci imaged on Chr7. Each pixel in the heatmap represents the Spearman correlation coefficient between the radial positioning and the above indicated quantity (rows) of imaged loci on Chr7 in the indicated cell type (columns). Transcription and chromatin accessibility were calculated from snRNA-seq data and snATAC-seq data (*59*). H3K9me3, H3K27ac, and H3K4me3 levels were calculated from Paired-Tag data **(*76*)**.

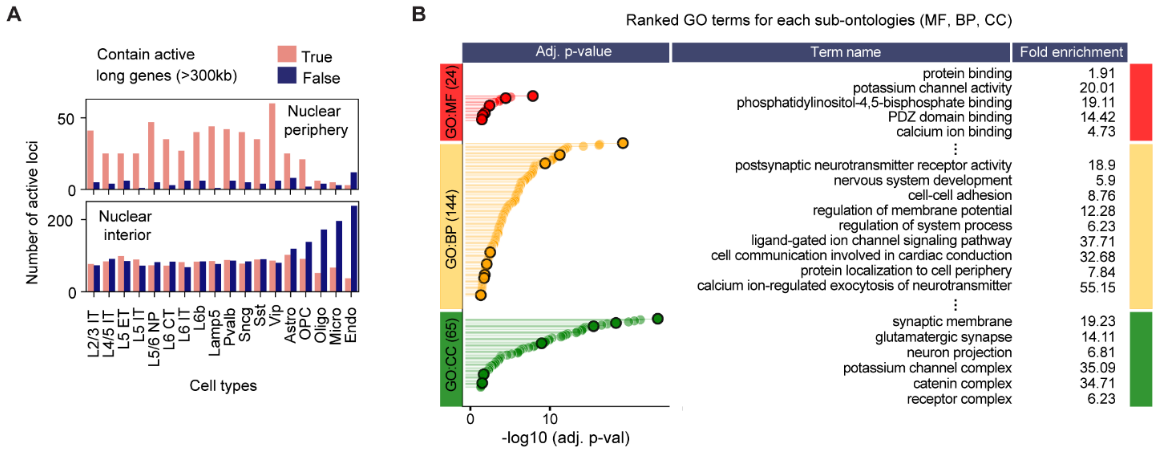

Fig. S17. Active expression of long genes in the nuclear periphery in neurons. (A) Preferential activation of long genes near the nuclear periphery in neurons. Top: Number of transcriptionally active loci near the nuclear periphery, grouped by whether they harbor actively expressed long genes in each cell type (Materials and Methods). Transcriptionally active loci are defined as loci in the top 25th percentile of cell-type median transcriptional level across all imaged loci, calculated from snRNA-seq data (*59*). Nuclear periphery loci are defined as loci in the top 20th percentile of cell-type median normalized nuclear radial position across all imaged loci. Long genes are defined as genes with >300 kb length. Bottom: similar to the top panel but for loci near the nuclear interior (defined as loci in the bottom 20th percentile of cell-type median normalized nuclear radial position across all imaged loci). (B) Gene Ontology (GO) analysis of actively expressed long genes in the nuclear periphery in neurons (Materials and Methods). Ranked significant GO terms ($p < 0.05$) identified from g:GOSt analysis (*126*) for each GO sub-ontologies are plotted. The total numbers of significant GO terms are indicated on the left bars, and the top driver GO terms (corresponding to circled dots) with their adjusted p-value and fold enrichment are indicated on the right. MF: molecular function; BP: biological process; CC: cellular component. Driver GO terms were identified from g:GOSt analysis.

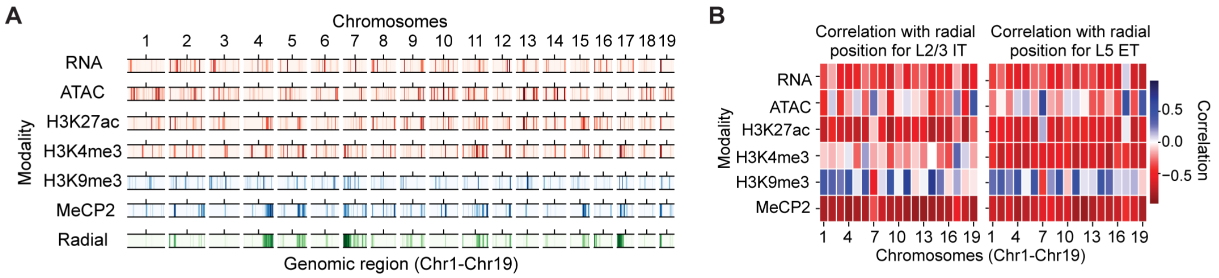

Fig. S18. Correlation between radial positions and various epigenetic modalities in neurons. (A) Transcriptional activity (RNA), chromatin accessibility (ATAC), histone modifications (H3K27ac, H3K4me3, and H3K9me3), MeCP2-binding, and nuclear radial positioning for individual genomic loci on Chr1-Chr19. For radial positioning, loci near the nuclear interior (30th percentile) are highlighted in color. For the other plots, loci enriched for the indicated signals (top 30th percentile) are highlighted in color. Transcriptional activity and chromatin accessibility were calculated from Ref.(*59*); H3K27ac, H3K4me3, and H3K9me3 levels were calculated from Ref.**(*76*)**; MeCP2-binding level was calculated from Ref. **(*77*)**. (B) The spearman correlation of transcriptional activity, chromatin accessibility, histone modification levels, and MeCP2-binding level with nuclear radial positioning for L2/3 IT (left) and L5 ET neurons (right). The Spearman correlation coefficient between the radial positions and the indicated quantities of all loci on the indicated chromosome are shown.

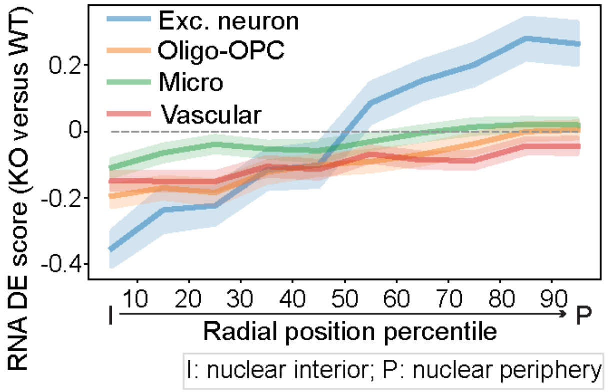

Fig. S19. Radial-position-dependent transcriptional regulation by MeCP2 when mapping to the radial positions in the visual cortex. Same as Fig. 5E for transcriptional changes (*83*), but with the nuclear radial position determined with DNA-MERFISH data of the visual cortex.

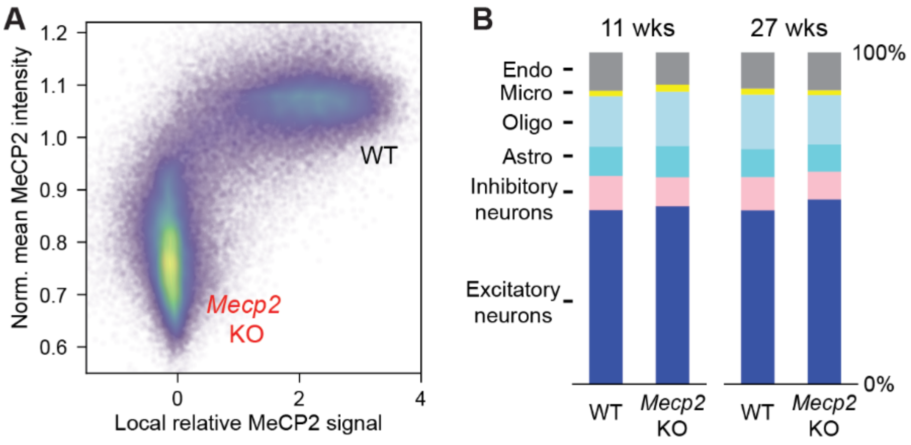

Fig. S20. Cell-type compositions of *Mecp2* WT and KO cells. (A) Identification of *Mecp2* WT and KO cells based on protein signals of MeCP2 in *Mecp2* +/- female mice. The dots in the scatterplot were colored by the estimated kernel density of the dot distribution. Normalized MeCP2 intensity (y axis) is the mean intensity of MeCP2 antibody signals per cell normalized by the mean signals of all cells belonging to the same cell types in the same experimental replicate. Local relative MeCP2 signal (x axis) is the relative MeCP2 antibody signal per cell against its local background. (B) Composition of six major cell types in WT and *Mecp*2 KO cells from the MOp region of *Mecp2* +/- female mice, at the indicated ages of the animals.

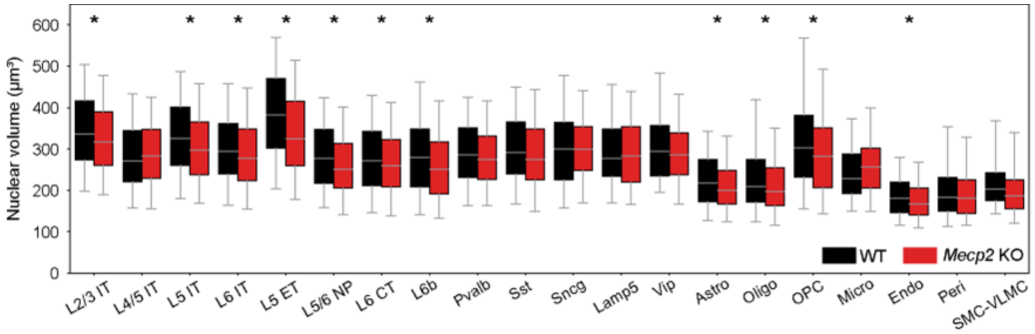

Fig. S21. The effects of *Mecp2* deletion on the nucleus sizes in different cell types. Boxplots for the distribution of nuclear volume of individual cells for WT and *Mecp2* KO cells in each indicated cell type. The center line, box, and whiskers in the boxplots represent the median, 25th-75th percentile, and the 5th-95th percentile, respectively. Mann-Whitney U test, corrected by the Benjamini-Hochberg method, was used for statistical significance analysis. *: $p<=0.05$.

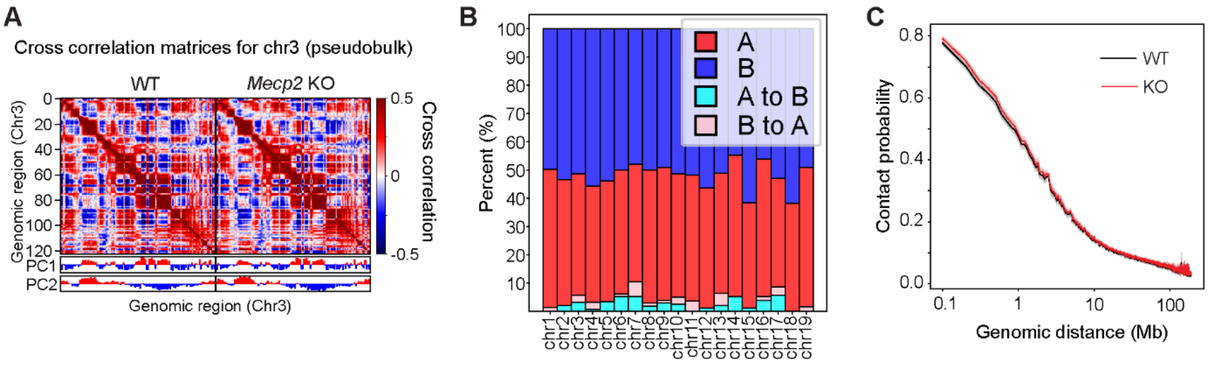

Fig. S22. Compartment analysis using DNA-MERFISH data after combining all imaged cells to generate pseudobulk data. (**A**) Cross-correlation matrices of an example chromosome (Chr3) for WT and *Mecp2* KO cells. The same principal component analysis was used for the matrices for both WT and KO and the first two PC values are plotted. The first PC gives A/B compartment boundaries, which are essentially identical between WT and *Mecp2* KO cells. (**B**) Distribution of the conservation and differences in compartment identities between WT and *Mecp2* KO cells across all 19 chromosomes. Overall, 96% of the imaged loci retained their A/B identity upon MeCP2 deletion and only ~4% loci have different A/B identities. (**C**) Probability of contact as a function of genomic distance for WT (black) and *Mecp2* KO (red) cells.

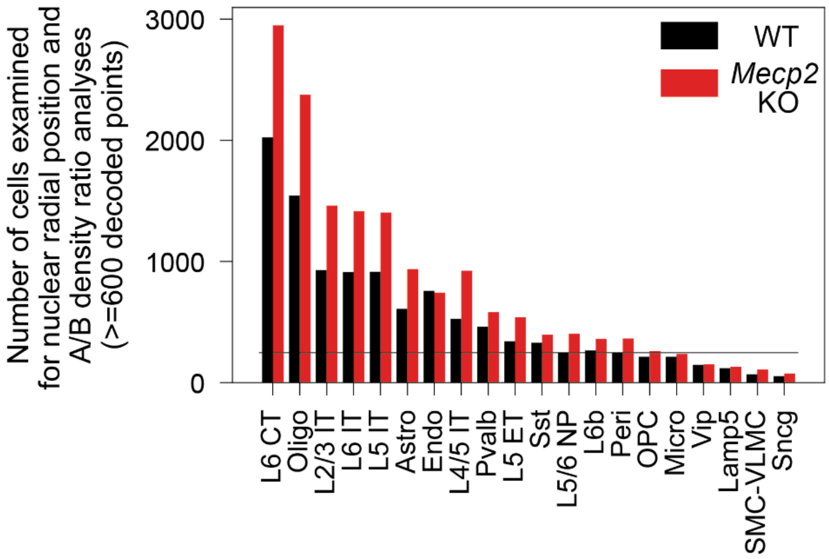

Fig. S23. Number of cells passing threshold for A/B density ratio and radial position analyses across cell types. The number of quantified cells (with at least 600 decoded chromatin loci per cell) for each cell type, for WT and *Mecp2* KO cells. A/B density ratio and radial position analyses were performed for cell types with image cell number above the cutoff line (black line).

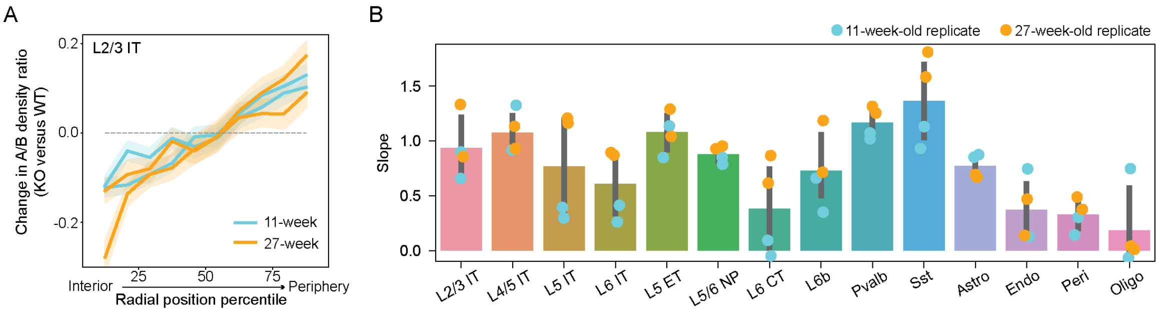

Fig. S24. The effect of *Mecp2* on chromatin organization across replicates. (**A**) Nuclear-radial-position-dependent changes in A/B density ratio upon *Mecp2* deletion in L2/3 IT neurons, separated by biological replicates and colored by ages. (**B**) Summary of the slope for changes in A/B density ratio over nuclear radial positions across examined cell types. Individual dot represents the slope calculated from each biological replicate and colored by age. We observed a trend in age-dependent effect in several neuronal types, but due to the relatively small sample size (2 biological replicates at each age), we refrain from concluding on age-dependent effect and grouped all replicates from both ages for analysis.

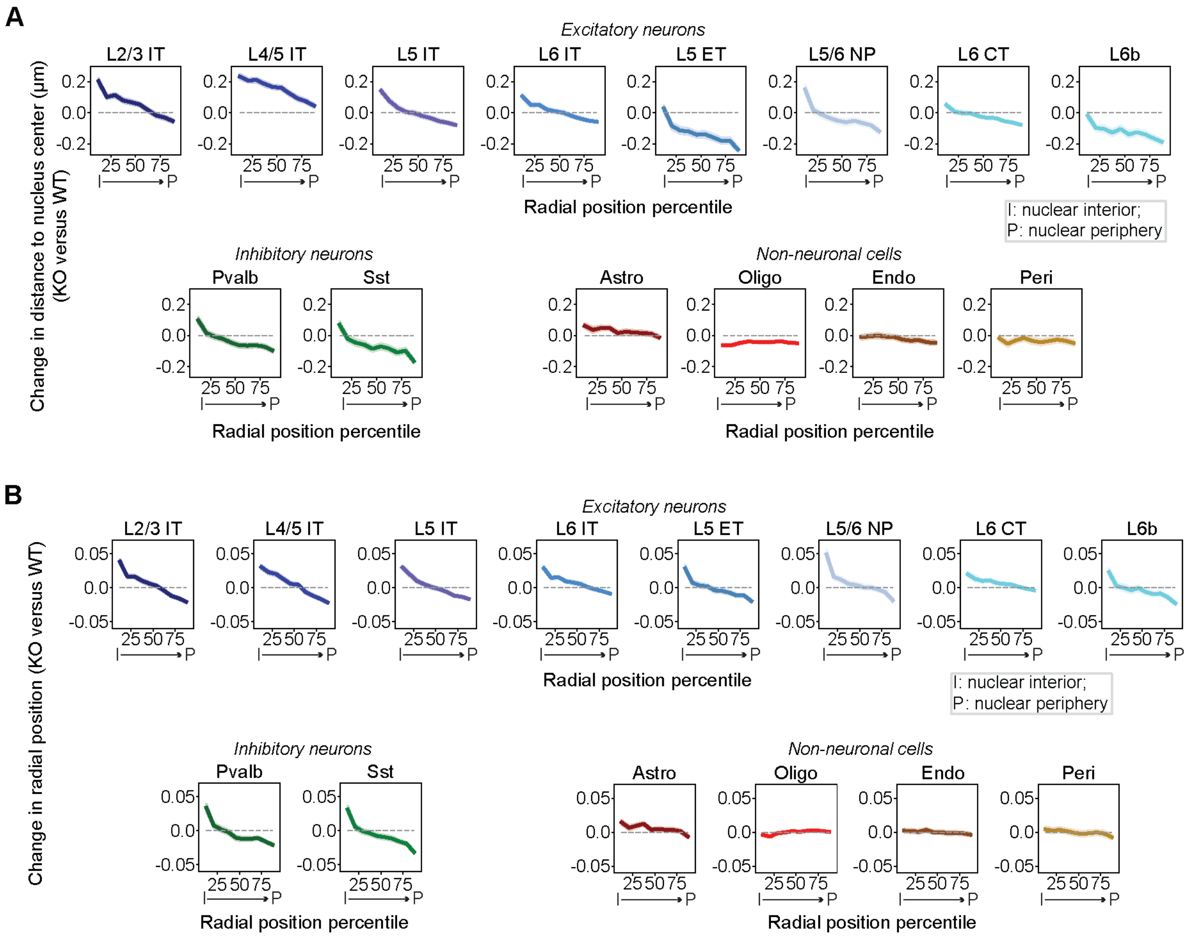

Fig. S25. Cell-type-dependent effects of *Mecp2* deletion on radial positioning. (**A**) Changes in distance to the nucleus center upon *Mecp2* deletion as a function of the normalized radial position of the imaged genomic loci in various cell types. All imaged loci were grouped into 10 equal bins based on their cell-type median of normalized radial positions in the WT cells. (**B**) Similar to (A), but for changes in normalized nuclear radial positions. For each locus, radial position change was calculated as the difference in the medians of normalized nuclear radial position in *Mecp2* KO over WT cells. In both (A) and (B), the line and shaded area represent the mean and the 95% confidence interval, respectively. The radial-position-dependent trend is similar in both measurements, but the absolute distance to the nuclear center metric could be influenced by the overall nuclear size or shape changes, whereas the normalized nuclear radial position metric removed these complications.

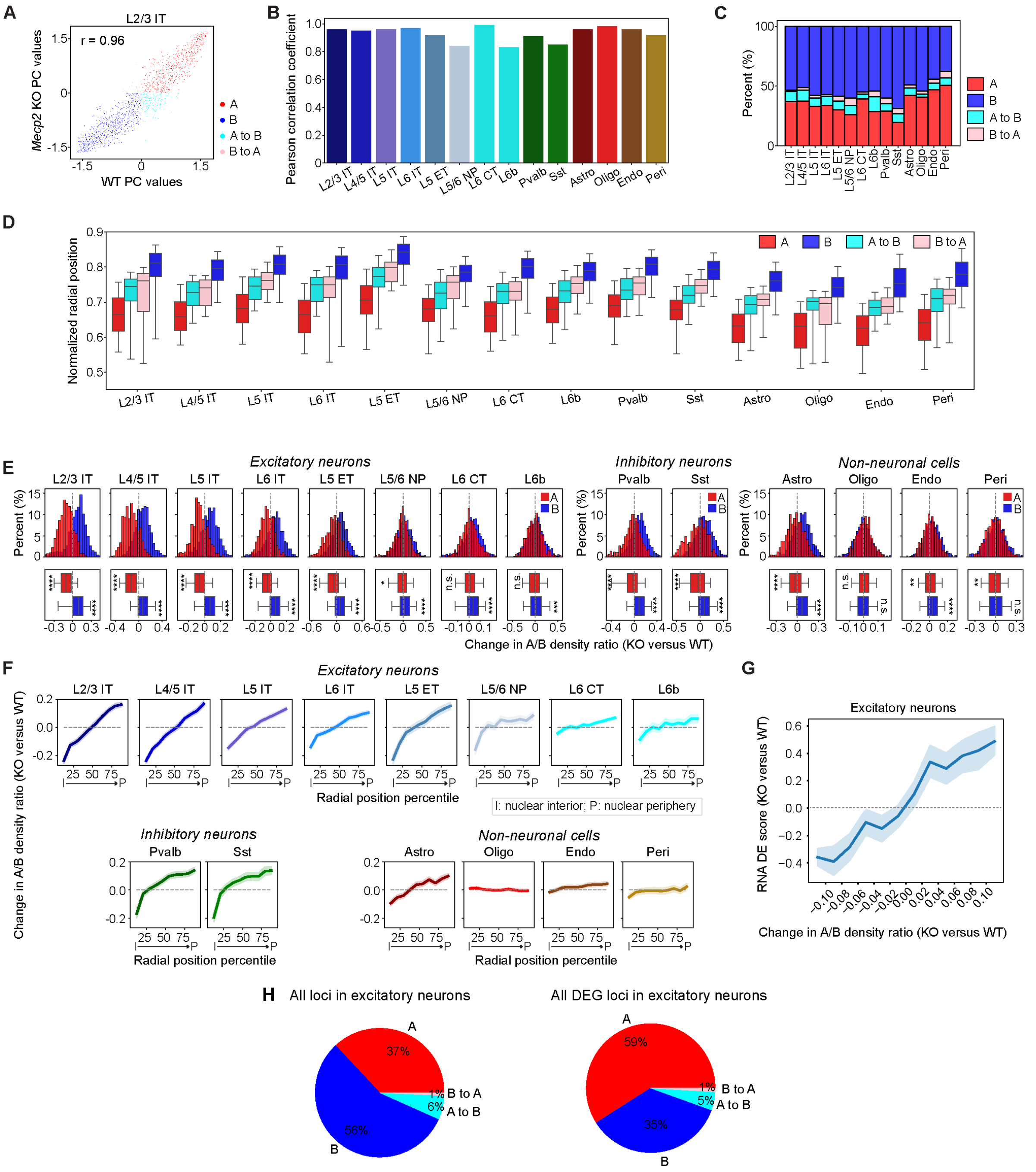

Fig. S26. The intermixing of A/B loci upon *Mecp2* deletion is independent of A/B switching. (**A**) Correlation between the compartment PC values from WT and *Mecp2* KO L2/3 IT neurons. The PC = 0 cutoff is used to define A and B compartments. A/B identity changes from WT to KO cells are color coded. Loci with A/B identity changes between WT and *Mecp2* KO cells usually have low compartment PC values. (**B**) Distribution of Pearson correlation coefficients between WT and *Mecp2* KO PC values across cell types. (**C**) Bar plots for the fraction of the A/B compartment conservations (e.g., A to A) and changes (e.g., A to B) from WT to *Mecp2* KO cells across cell types. (**D**) Boxplots for the distribution of normalized radial positions of loci with conserved A/B identities and changed A/B identities. (**E to G**) Similar to Fig. 6 D to F, but

only considering loci with conserved A/B identities. Student's t-tests against null hypothesis of mean 0 with Bonferroni corrections were used for statistical significance evaluation. *:p<=0.05; **: p<=0.01; ***: p<=0.001; ****: p<=0.0001; n.s.: p>0.05. (**H**) Distribution of A/B conservation and A/B changes among all imaged loci (left) and all imaged loci that harbor differentially expressed gene (DEG) between *Mecp2* KO and WT cells in excitatory neurons.

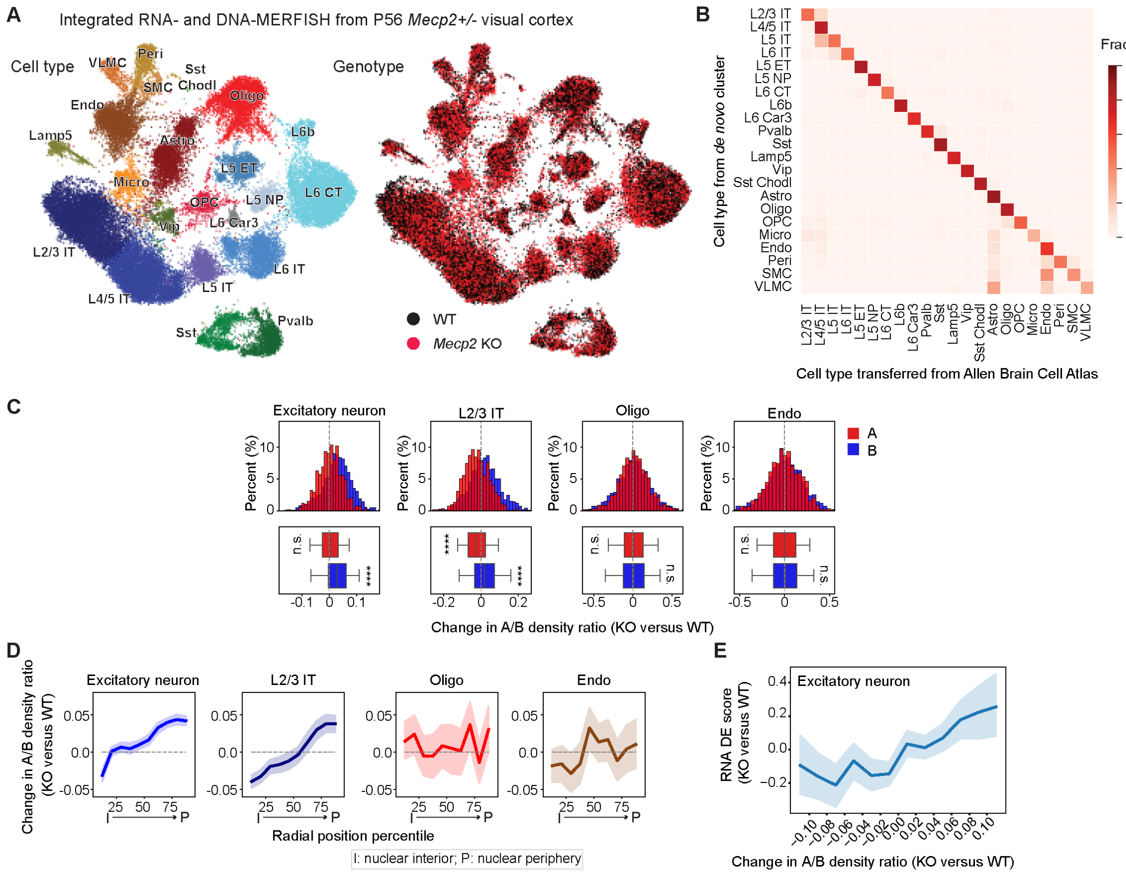

Fig. S27. The effect of *Mecp2* deletion on chromatin organization in the visual cortex. (**A**) UMAP representation of cell types (left) and genotypes (right) from integrated RNA- and DNA-MERFISH on the visual cortices of P56 *Mecp2+/-* mice. (**B**) Confusion matrix of the cell types identified from *de novo* clustering and from label transfer from Allen Brain Cell atlas (*57, 123*) (**C**) Histograms of local A/B density ratio changes upon *Mecp2* deletion for compartment-A (red) and compartment-B (blue) loci in representative cell types. Due to the relatively small number of cells imaged, subclass-level analysis was conducted on the most abundant neuronal type – L2/3 IT neurons and the most abundant non-neuronal cells – oligodendrocytes (Oligo) and endothelial (Endo) cells. Analysis was also conducted on all excitatory neurons. Student's t-tests against null hypothesis of mean 0 with Bonferroni corrections were used for statistical significance evaluation. ****: p<=0.0001; n.s.: p>0.05. (**D**) Change in A/B density ratio upon *Mecp2* deletion as a function of the normalized radial position of the genomic loci in the cell types as in (C) in the visual cortex. All imaged loci were grouped into 10 equal bins based on their cell-type median normalized radial positions in the WT cells. (**E**) Correlation between transcriptional changes and local A/B density ratio changes upon *Mecp2* deletion in excitatory neurons. Genes are grouped based on their local A/B density ratio changes indicated in the x-axis. For each gene, the differential expression (DE) score for transcription change between KO and WT cells was calculated using snRNA-seq data as in Fig. 5E. The effect upon *Mecp2* deletion observed for the visual cortex is relatively weak compared to that observed for the MOp as shown in Fig. 6, possibly because of the younger age of mice used for analysis in the visual cortex (8 weeks), compared to 11 weeks and 27 weeks old mice used for the MOp measurements.

Supplementary table captions

Table S1. Encoding-probe libraries for integrated RNA- and DNA-MERFISH.

A list of all the template oligonucleotides used to make the encoding probes for RNA- and DNA-MERFISH imaging. The file contains five spreadsheets – "RNA MERFISH probes", "Genome chromatin probes", "TSS chromatin probes", "Super-enhancer chromatin probes", and "TSS chromatin pbs (probes) by MERFISH". (1) "RNA MERFISH probes" contains all template sequences for making encoding probes for RNA-MERFISH. (2) "Genome chromatin probes" contains all template sequences for making encoding probes for DNA-MERFISH imaging of the 988 genomic loci evenly distributed across the genome. (3) "TSS chromatin probes" contains all template sequences for making encoding probes for sequential DNA-FISH imaging of the TSSs of 28 cell-type marker genes. (4) "Super-enhancer chromatin probes" contains all template sequences for making encoding probes for DNA-MERFISH imaging of the 965 super-enhancer loci. (5) "TSS chromatin pbs (probes) by MERFISH" contains all template sequences for making encoding probes for DNA-MERFSH imaging of TSSs of 28 cell-type marker genes.

Table S2. Adaptor probes for integrated RNA- and DNA-MERFISH.

The file contains four spreadsheets – "RNA MERFISH", "Genome chromatin", "TSS chromatin" and "Super-enhancer chromatin". Each spreadsheet corresponds to a list of the adaptor probes that were used to hybridize the readout sequences on the encoding probes, from each library described in Table S1. The names of these adaptor probes are formatted in the following way unless otherwise described. Using the first adaptor probe name from the "RNA MERFISH" sheet as an example ("Bit-1-RS0015_2xStv_82rc"), "Bit-1" corresponds to the first bit in the 22-bit RNA-MERFISH imaging. "RS0015" corresponds to the name of a unique readout sequence on the encoding probes (see "name" column in Table S1), and "2xStv_79rc" corresponds to two binding sequences of the dye-conjugated common readout probe named "Stv_79" (see "name" column in Table S3). The last adaptor probe name in the "RNA MERFISH" sheet ("Bit-polyT-RS2179_2xStv_82rc") corresponds to the adaptor probe that target polyA-anchor probe (/5Acryd/TTGAGTGGATGGAGTGTAATT+TT+TT+TT+TT+TT+TT+TT+TT+TT+T).

Table S3. Readout probes and PCR primers for integrated RNA- and DNA-MERFISH.

The file contains two spreadsheets – "Common readouts" and "Primers". (1). "Common readouts" contains the names and sequences of three dye-conjugated common readout probes, which were used to hybridize the two binding sequences on each adaptor probe, as described in Table S2. (2) "Primers" contains all forward and reverse primer pairs used for amplification of the template oligonucleotides, for each library described in Table S1. For "Genome chromatin" library, the template oligonucleotides were divided into three sets ("sets 1 and 2", "set 3", and "set 4) by a ratio of ~ 2:1:1 for the total number of probes from each set.

REFERENCES AND NOTES

1. K. H. Chow, R. E. Factor, K. S. Ullman, The nuclear envelope environment and its cancer connections. *Nat. Rev. Cancer* **12**, 196–209 (2012).

2. B. M. Skinner, E. E. P. Johnson, Nuclear morphologies: Their diversity and functional relevance. *Chromosoma* **126**, 195–212 (2017).

3. W. A. Bickmore, The spatial organization of the human genome. *Annu. Rev. Genomics Hum. Genet.* **14**, 67–84 (2013).

4. M. Levine, C. Cattoglio, R. Tjian, Looping back to leap forward: Transcription enters a new era. *Cell* **157**, 13–25 (2014).

5. B. van Steensel, A. S. Belmont, Lamina-associated domains: Links with chromosome architecture, heterochromatin, and gene repression. *Cell* **169**, 780–791 (2017).

6. D. Hnisz, K. Shrinivas, R. A. Young, A. K. Chakraborty, P. A. Sharp, A phase separation model for transcriptional control. *Cell* **169**, 13–23 (2017).

7. M. Mir, W. Bickmore, E. E. M. Furlong, G. Narlikar, Chromatin topology, condensates and gene regulation: Shifting paradigms or just a phase? *Development* **146**, dev182766 (2019).

8. M. Lakadamyali, M. P. Cosma, Visualizing the genome in high resolution challenges our textbook understanding. *Nat. Methods* **17**, 371–379 (2020).

9. T. Misteli, The self-organizing genome: Principles of genome architecture and function. *Cell* **183**, 28–45 (2020).

10. J. Dekker, F. Alber, S. Aufmkolk, B. J. Beliveau, B. G. Bruneau, A. S. Belmont, L. Bintu, A. Boettiger, R. Calandrelli, C. M. Disteche, D. M. Gilbert, T. Gregor, A. S. Hansen, B. Huang, D. Huangfu, R. Kalhor, C. S. Leslie, W. Li, Y. Li, J. Ma, W. S. Noble, P. J. Park, J. E. Phillips-Cremins, K. S. Pollard, S. M. Rafelski, B. Ren, Y. Ruan, Y. Shav-Tal, Y. Shen, J. Shendure, X. Shu, C. Strambio-De-Castillia, A. Vertii, H. Zhang,

S. Zhong, Spatial and temporal organization of the genome: Current state and future aims of the 4D nucleome project. *Mol. Cell* **83**, 2624–2640 (2023).

11. J. Dekker, L. Mirny, The 3D genome as moderator of chromosomal communication. *Cell* **164**, 1110–1121 (2016).

12. P. H. Krijger, W. de Laat, Regulation of disease-associated gene expression in the 3D genome. *Nat. Rev. Mol. Cell Biol.* **17**, 771–782 (2016).

13. M. Yu, B. Ren, The three-dimensional organization of mammalian genomes. *Annu. Rev. Cell Dev. Biol.* **33**, 265–289 (2017).

14. G. Cavalli, E. Heard, Advances in epigenetics link genetics to the environment and disease. *Nature* **571**, 489–499 (2019).

15. R. Kempfer, A. Pombo, Methods for mapping 3D chromosome architecture. *Nat. Rev. Genet.* **21**, 207–226 (2020).

16. X. Zhuang, Spatially resolved single-cell genomics and transcriptomics by imaging. *Nat. Methods* **18**, 18–22 (2021).

17. E. Lieberman-Aiden, N. L. van Berkum, L. Williams, M. Imakaev, T. Ragoczy, A. Telling, I. Amit, B. R. Lajoie, P. J. Sabo, M. O. Dorschner, R. Sandstrom, B. Bernstein, M. A. Bender, M. Groudine, A. Gnirke, J. Stamatoyannopoulos, L. A. Mirny, E. S. Lander, J. Dekker, Comprehensive mapping of long-range interactions reveals folding principles of the human genome. *Science* **326**, 289–293 (2009).

18. J. R. Dixon, S. Selvaraj, F. Yue, A. Kim, Y. Li, Y. Shen, M. Hu, J. S. Liu, B. Ren, Topological domains in mammalian genomes identified by analysis of chromatin interactions. *Nature* **485**, 376–380 (2012).

19. E. P. Nora, B. R. Lajoie, E. G. Schulz, L. Giorgetti, I. Okamoto, N. Servant, T. Piolot, N. L. van Berkum, J. Meisig, J. Sedat, J. Gribnau, E. Barillot, N. Bluthgen, J. Dekker, E.

Heard, Spatial partitioning of the regulatory landscape of the X-inactivation centre. *Nature* **485**, 381–385 (2012).

20. T. Sexton, E. Yaffe, E. Kenigsberg, F. Bantignies, B. Leblanc, M. Hoichman, H. Parrinello, A. Tanay, G. Cavalli, Three-dimensional folding and functional organization principles of the drosophila genome. *Cell* **148**, 458–472 (2012).

21. S. S. P. Rao, M. H. Huntley, N. C. Durand, E. K. Stamenova, I. D. Bochkov, J. T. Robinson, A. L. Sanborn, I. Machol, A. D. Omer, E. S. Lander, E. L. Aiden, A 3D map of the human genome at kilobase resolution reveals principles of chromatin looping. *Cell* **159**, 1665–1680 (2014).

22. J. Kind, L. Pagie, S. S. de Vries, L. Nahidiazar, S. S. Dey, M. Bienko, Y. Zhan, B. Lajoie, C. A. de Graaf, M. Amendola, G. Fudenberg, M. Imakaev, L. A. Mirny, K. Jalink, J. Dekker, A. van Oudenaarden, B. van Steensel, Genome-wide maps of nuclear lamina interactions in single human cells. *Cell* **163**, 134–147 (2015).

23. S. Y. Wang, J. H. Su, B. J. Beliveau, B. Bintu, J. R. Moffitt, C. T. Wu, X. W. Zhuang, Spatial organization of chromatin domains and compartments in single chromosomes. *Science* **353**, 598–602 (2016).

24. L. Z. Tan, D. Xing, C. H. Chang, H. Li, S. Xie, Three-dimensional genome structures of single diploid human cells. *Science* **361**, 924–928 (2018).

25. J. H. Su, P. Zheng, S. S. Kinrot, B. Bintu, X. W. Zhuang, Genome-scale imaging of the 3D organization and transcriptional activity of chromatin. *Cell* **182**, 1641–1659.e26 (2020).

26. T. J. Stevens, D. Lando, S. Basu, L. P. Atkinson, Y. Cao, S. F. Lee, M. Leeb, K. J. Wohlfahrt, W. Boucher, A. O'Shaughnessy-Kirwan, J. Cramard, A. J. Faure, M. Ralser, E. Blanco, L. Morey, M. Sansó, M. G. S. Palayret, B. Lehner, L. Di Croce, A. Wutz, B. Hendrich, D. Klenerman, E. D. Laue, 3D structures of individual mammalian genomes studied by single-cell Hi-C. *Nature* **544**, 59–64 (2017).

27. C. Bersaglieri, J. Kresoja-Rakic, S. Gupta, D. Bär, R. Kuzyakiv, M. Panatta, R. Santoro, Genome-wide maps of nucleolus interactions reveal distinct layers of repressive chromatin domains. *Nat. Commun.* **13**, 1483 (2022).

28. S. A. Quinodoz, N. Ollikainen, B. Tabak, A. Palla, J. M. Schmidt, E. Detmar, M. M. Lai, A. A. Shishkin, P. Bhat, Y. Takei, V. Trinh, E. Aznauryan, P. Russell, C. Cheng, M. Jovanovic, A. Chow, L. Cai, P. McDonel, M. Garber, M. Guttman, Higher-order inter-chromosomal hubs shape 3D genome organization in the nucleus. *Cell* **174**, 744–757.e24 (2018).

29. J. R. Dixon, I. Jung, S. Selvaraj, Y. Shen, J. E. Antosiewicz-Bourget, A. Y. Lee, Z. Ye, A. Kim, N. Rajagopal, W. Xie, Y. R. Diao, J. Liang, H. M. Zhao, V. V. Lobanenkov, J. R. Ecker, J. A. Thomson, B. Ren, Chromatin architecture reorganization during stem cell differentiation. *Nature* **518**, 331–336 (2015).

30. B. Bonev, N. M. Cohen, Q. Szabo, L. Fritsch, G. L. Papadopoulos, Y. Lubling, X. L. Xu, X. D. Lv, J. P. Hugnot, A. Tanay, G. Cavalli, Multiscale 3D genome rewiring during mouse neural development. *Cell* **171**, 557–572.e24 (2017).

31. W. Winick-Ng, A. Kukalev, I. Harabula, L. Zea-Redondo, D. Szabo, M. Meijer, L. Serebreni, Y. N. Zhang, S. Bianco, A. M. Chiariello, I. Irastorza-Azcarate, C. J. Thieme, T. M. Sparks, S. Carvalho, L. Fiorillo, F. Musella, E. Irani, E. T. Triglia, A. A. Kolodziejczyk, A. Abentung, G. Apostolova, E. J. Paul, V. Franke, R. Kempfer, A. Akalin, S. A. Teichmann, G. Dechant, M. A. Ungless, M. Nicodemi, L. Welch, G. Castelo-Branco, A. Pombo, Cell-type specialization is encoded by specific chromatin topologies. *Nature* **599**, 684–691 (2021).

32. L. Z. Tan, W. P. Ma, H. G. Wu, Y. H. Zheng, D. Xing, R. Chen, X. Li, N. Daley, K. Deisseroth, X. S. Xie, Changes in genome architecture and transcriptional dynamics progress independently of sensory experience during post-natal brain development. *Cell* **184**, 741–758.e17 (2021).

33. L. Tan, J. Shi, S. Moghadami, B. Parasar, C. P. Wright, Y. Seo, K. Vallejo, I. Cobos, L. Duncan, R. Chen, K. Deisseroth, Lifelong restructuring of 3D genome architecture in cerebellar granule cells. *Science* **381**, 1112–1119 (2023).

34. W. Tian, J. Zhou, A. Bartlett, Q. Zeng, H. Liu, R. G. Castanon, M. Kenworthy, J. Altshul, C. Valadon, A. Aldridge, J. R. Nery, H. Chen, J. Xu, N. D. Johnson, J. Lucero, J. K. Osteen, N. Emerson, J. Rink, J. Lee, Y. E. Li, K. Siletti, M. Liem, N. Claffey, C. O'Connor, A. M. Yanny, J. Nyhus, N. Dee, T. Casper, N. Shapovalova, D. Hirschstein, S. L. Ding, R. Hodge, B. P. Levi, C. D. Keene, S. Linnarsson, E. Lein, B. Ren, M. M. Behrens, J. R. Ecker, Single-cell DNA methylation and 3D genome architecture in the human brain. *Science* **382**, eadf5357 (2023).

35. H. Q. Liu, Q. R. Zeng, J. T. Zhou, A. Bartlett, B. A. Wang, P. Berube, W. Tian, M. Kenworthy, J. Altshul, J. R. Nery, H. M. Chen, R. G. Castanon, S. P. Zu, Y. E. Li, J. Lucero, J. K. Osteen, A. Pinto-Duarte, J. S. Lee, J. Rink, S. L. Cho, N. Emerson, M. Nunn, C. O'Connor, Z. H. Wu, I. Stoica, Z. Z. Yao, K. A. Smith, B. Tasic, C. Y. Luo, J. R. Dixon, H. K. Zeng, B. Ren, M. M. Behrens, J. R. Ecker, Single-cell DNA methylome and 3D multi-omic atlas of the adult mouse brain. *Nature* **624**, 366–377 (2023).

36. Y. Takei, S. W. Zheng, J. N. Yun, S. Shah, N. Pierson, J. White, S. Schindler, C. H. Tischbirek, G. C. Yuan, L. Cai, Single-cell nuclear architecture across cell types in the mouse brain. *Science* **374**, 586–594 (2021).

37. X. Z. Wen, Z. F. Luo, W. X. Zhao, R. Calandrelli, T. C. Nguyen, X. Y. Wan, J. L. C. Richard, S. Zhong, Single-cell multiplex chromatin and RNA interactions in ageing human brain. *Nature* **628**, 648–656 (2024).

38. T. M. Zhou, R. C. Zhang, D. Y. Jia, R. T. Doty, A. D. Munday, D. Gao, L. Xin, J. L. Abkowitz, Z. J. Duan, J. Ma, GAGE-seq concurrently profiles multiscale 3D genome organization and gene expression in single cells. *Nat. Genet.* **56**, 1701–1711 (2024).

39. R. Stadhouders, G. J. Filion, T. Graf, Transcription factors and 3D genome conformation in cell-fate decisions. *Nature* **569**, 345–354 (2019).

40. N. Gamarra, G. J. Narlikar, Collaboration through chromatin: Motors of transcription and chromatin structure. *J. Mol. Biol.* **433**, 166876 (2021).

41. J. D. Lewis, R. R. Meehan, W. J. Henzel, I. Maurer-Fogy, P. Jeppesen, F. Klein, A. Bird, Purification, sequence, and cellular localization of a novel chromosomal protein that binds to methylated DNA. *Cell* **69**, 905–914 (1992).

42. Y. Liu, A. Flamier, G. W. Bell, A. J. Diao, T. W. Whitfield, H. C. Wang, Y. Wu, F. Schulte, M. Friesen, R. Guo, M. Mitalipova, X. S. Liu, S. M. Vos, R. A. Young, R. Jaenisch, MECP2 directly interacts with RNA polymerase II to modulate transcription in human neurons. *Neuron* **112**, 1943–1958.e10 (2024).

43. L. A. Lavery, H. Y. Zoghbi, The distinct methylation landscape of maturing neurons and its role in Rett syndrome pathogenesis. *Curr. Opin. Neurobiol.* **59**, 180–188 (2019).

44. R. Tillotson, A. Bird, The molecular basis of MeCP2 function in the brain. *J. Mol. Biol.* **432**, 1602–1623 (2020).

45. R. E. Amir, I. B. Van den Veyver, M. Wan, C. Q. Tran, U. Francke, H. Y. Zoghbi, Rett syndrome is caused by mutations in X-linked MECP2, encoding methyl-CpG-binding protein 2. *Nat. Genet.* **23**, 185–188 (1999).

46. W. A. Gold, R. Krishnarajy, C. Ellaway, J. Christodoulou, Rett syndrome: A genetic update and clinical review focusing on comorbidities. *ACS Chem. Nerosci.* **9**, 167–176 (2018).

47. H. Y. Zoghbi, Rett syndrome and the ongoing legacy of close clinical observation. *Cell* **167**, 293–297 (2016).

48. H. Leonard, S. Cobb, J. Downs, Clinical and biological progress over 50 years in Rett syndrome. *Nat. Rev. Neurol.* **13**, 37–51 (2017).

49. B. Suter, D. Treadwell-Dearing, H. Zoghbi, D. Glaze, J. Neul, Brief report: *MECP2* mutations in people without rett syndrome. *J. Autism Dev. Disord.* **44**, 703–711 (2014).

50. C. H. Li, E. L. Coffey, A. Dall'Agnese, N. M. Hannett, X. Tang, J. E. Henninger, J. M. Platt, O. Oksuz, A. V. Zamudio, L. K. Afeyan, J. Schuijers, X. S. Liu, S. Markoulaki, T. Lungjangwa, G. LeRoy, D. S. Svoboda, E. Wogram, T. I. Lee, R. Jaenisch, R. A. Young, MeCP2 links heterochromatin condensates and neurodevelopmental disease. *Nature* **586**, 440–444 (2020).

51. L. Wang, M. Hu, M. Q. Zuo, J. Zhao, D. Wu, L. Huang, Y. Wen, Y. Li, P. Chen, X. Bao, M. Q. Dong, G. Li, P. Li, Rett syndrome-causing mutations compromise MeCP2-mediated liquid-liquid phase separation of chromatin. *Cell Res.* **30**, 393–407 (2020).

52. M. W. Linhoff, S. K. Garg, G. Mandel, A high-resolution imaging approach to investigate chromatin architecture in complex tissues. *Cell* **163**, 246–255 (2015).

53. A. Ito-Ishida, S. A. Baker, R. V. Sillitoe, Y. L. Sun, J. Zhou, Y. Ono, J. Iwakiri, M. Yuzaki, H. Y. Zoghbi, MeCP2 levels regulate the 3D structure of heterochromatic foci in mouse neurons. *J. Neurosci.* **40**, 8746–8766 (2020).

54. K. H. Chen, A. N. Boettiger, J. R. Moffitt, S. Y. Wang, X. W. Zhuang, Spatially resolved, highly multiplexed RNA profiling in single cells. *Science* **348**, aaa6090 (2015).

55. J. R. Moffitt, D. Bambah-Mukku, S. W. Eichhorn, E. Vaughn, K. Shekhar, J. D. Perez, N. D. Rubinstein, J. Hao, A. Regev, C. Dulac, X. W. Zhuang, Molecular, spatial, and functional single-cell profiling of the hypothalamic preoptic region. *Science* **362**, eaau5324 (2018).

56. R. X. Fang, C. L. Xia, J. L. Close, M. Zhan, J. He, Z. K. Huang, A. R. Halpern, B. Long, J. A. Miller, E. S. Lein, X. W. Zhuang, Conservation and divergence of cortical cell organization in human and mouse revealed by MERFISH. *Science* **377**, 56–62 (2022).

57. M. Zhang, X. J. Pan, W. Jung, A. R. Halpern, S. W. Eichhorn, Z. Y. Lei, L. Cohen, K. A. Smith, B. Tasic, Z. Z. Yao, H. K. Zeng, X. W. Zhuang, Molecularly defined and spatially resolved cell atlas of the whole mouse brain. *Nature* **624**, 343–354 (2023).

58. BRAIN Initiative Cell Census Network, A multimodal cell census and atlas of the mammalian primary motor cortex. *Nature* **598**, 86–102 (2021).

59. Z. Z. Yao, H. Q. Liu, F. M. Xie, S. Fischer, R. S. Adkins, A. I. Aldridge, S. A. Ament, A. Bartlett, M. M. Behrens, K. Van den Berge, D. Bertagnolli, H. R. de Bezieux, T. Biancalani, A. S. Booeshaghi, H. C. Bravo, T. Casper, C. Colantuoni, J. Crabtree, H. Creasy, K. Crichton, M. Crow, N. Dee, E. L. Dougherty, W. I. Doyle, S. Dudoit, R. X. Fang, V. Felix, O. Fong, M. Giglio, J. Goldy, M. Hawrylycz, B. R. Herb, R. Hertzano, X. M. Hou, Q. W. Hu, J. Kancherla, M. Kroll, K. Lathia, Y. E. Li, J. D. Lucero, C. Y. Luo, A. Mahurkar, D. McMillen, N. M. Nadaf, J. R. Nery, T. N. Nguyen, S. Y. Niu, V. Ntranos, J. Orvis, J. K. Osteen, T. Pham, A. Pinto-Duarte, O. Poirion, S. Preissl, E. Purdom, C. Rimorin, D. Risso, A. C. Rivkin, K. Smith, K. Street, J. Sulc, V. Svensson, M. Tieu, A. Torkelson, H. Tung, E. D. Vaishnav, C. R. Vanderburg, C. van Velthoven, X. X. Wang, O. R. White, Z. J. Huang, P. V. Kharchenko, L. Pachter, J. Ngai, A. Regev, B. Tasic, J. D. Welch, J. Gillis, E. Z. Macosko, B. Ren, J. R. Ecker, H. K. Zeng, E. A. Mukamel, A transcriptomic and epigenomic cell atlas of the mouse primary motor cortex. *Nature* **598**, 103–110 (2021).

60. H. Q. Liu, J. T. Zhou, W. Tian, C. Y. Luo, A. Bartlett, A. Aldridge, J. Lucero, J. K. Osteen, J. R. Nery, H. M. Chen, A. Rivkin, R. G. Castanon, B. Clock, Y. E. Li, X. M. Hou, O. B. Poirion, S. Preissl, A. Pinto-Duarte, C. O'Connor, L. Boggeman, C. Fitzpatrick, M. Nunn, E. A. Mukamel, Z. Z. Zhang, E. M. Callaway, B. Ren, J. R. Dixon, M. M. Behrens, J. R. Ecker, DNA methylation atlas of the mouse brain at single-cell resolution. *Nature* **598**, 120–128 (2021).

61. Y. E. Li, S. Preissl, X. M. Hou, Z. Y. Zhang, K. Zhang, Y. J. Qiu, O. B. Poirion, B. Li, J. Chiou, H. Q. Liu, A. Pinto-Duarte, N. Kubo, X. Y. Yang, R. X. Fang, X. X. Wang, J. Y. Han, J. Lucero, Y. M. Yan, M. Miller, S. Kuan, D. Gorkin, K. J. Gaulton, Y. Shen, M. Nunn, E. A. Mukamel, M. M. Behrens, J. R. Ecker, B. Ren, An atlas of gene regulatory elements in adult mouse cerebrum. *Nature* **598**, 129–136 (2021).

62. M. Zhang, S. W. Eichhorn, B. Zingg, Z. Z. Yao, K. Cotter, H. K. Zeng, H. W. Dong, X. W. Zhuang, Spatially resolved cell atlas of the mouse primary motor cortex by MERFISH. *Nature* **598**, 137–143 (2021).

63. J. R. Moffitt, J. J. Hao, D. Bambah-Mukku, T. Lu, C. Dulac, X. W. Zhuang, High-performance multiplexed fluorescence in situ hybridization in culture and tissue with matrix imprinting and clearing. *Proc. Natl. Acad. Sci. U.S.A.* **113**, 14456–14461 (2016).

64. W. A. Whyte, D. A. Orlando, D. Hnisz, B. J. Abraham, C. Y. Lin, M. H. Kagey, P. B. Rahl, T. I. Lee, R. A. Young, Master transcription factors and mediator establish super-enhancers at key cell identity genes. *Cell* **153**, 307–319 (2013).

65. D. Hnisz, B. J. Abraham, T. I. Lee, A. Lau, V. Saint-Andre, A. A. Sigova, H. A. Hoke, R. A. Young, Super-enhancers in the control of cell identity and disease. *Cell* **155**, 934–947 (2013).

66. N. R. Zemke, E. J. Armand, W. L. Wang, S. Y. Lee, J. T. Zhou, Y. E. Li, H. Q. Liu, W. Tian, J. R. Nery, R. G. Castanon, A. Bartlett, J. K. Osteen, D. F. Li, X. Y. Zhuo, V. C. Xu, L. Chang, K. Y. Dong, H. S. Indralingam, J. A. Rink, Y. Xie, M. Miller, F. M. Krienen, Q. G. Zhang, N. Taskin, J. A. T. Ting, G. P. Feng, S. A. Mccarroll, E. M. Callaway, T. Wang, E. S. Lein, M. M. Behrens, J. R. Ecker, B. Ren, Conserved and divergent gene regulatory programs of the mammalian neocortex. *Nature* **624**, 390–402 (2023).

67. B. B. Jia, A. Jussila, C. Kern, Q. Zhu, B. Ren, A spatial genome aligner for resolving chromatin architectures from multiplexed DNA FISH. *Nat. Biotechnol.* **41**, 1004–1017 (2023).

68. M. A. Garcia-Cabezas, H. Barbas, B. Zikopoulos, Parallel development of chromatin patterns, neuron morphology, and connections: Potential for disruption in autism. *Front. Neuroanat.* **12**, 70 (2018).

69. S. R. Y. Cajal, D. Azoulay, *Histology Of The Nervous System: Of Man And Vertebrates* (Oxford Academic, 1995).

70. T. E. Bakken, N. L. Jorstad, Q. Hu, B. B. Lake, W. Tian, B. E. Kalmbach, M. Crow, R. D. Hodge, F. M. Krienen, S. A. Sorensen, J. Eggermont, Z. Yao, B. D. Aevermann, A. I. Aldridge, A. Bartlett, D. Bertagnolli, T. Casper, R. G. Castanon, K. Crichton, T. L. Daigle, R. Dalley, N. Dee, N. Dembrow, D. Diep, S. L. Ding, W. Dong, R. Fang, S. Fischer, M. Goldman, J. Goldy, L. T. Graybuck, B. R. Herb, X. Hou, J. Kancherla, M. Kroll, K. Lathia, B. van Lew, Y. E. Li, C. S. Liu, H. Liu, J. D. Lucero, A. Mahurkar, D. McMillen, J. A. Miller, M. Moussa, J. R. Nery, P. R. Nicovich, S. Y. Niu, J. Orvis, J. K. Osteen, S. Owen, C. R. Palmer, T. Pham, N. Plongthongkum, O. Poirion, N. M. Reed, C. Rimorin, A. Rivkin, W. J. Romanow, A. E. Sedeno-Cortes, K. Siletti, S. Somasundaram, J. Sulc, M. Tieu, A. Torkelson, H. Tung, X. Wang, F. Xie, A. M. Yanny, R. Zhang, S. A. Ament, M. M. Behrens, H. C. Bravo, J. Chun, A. Dobin, J. Gillis, R. Hertzano, P. R. Hof, T. Hollt, G. D. Horwitz, C. D. Keene, P. V. Kharchenko, A. L. Ko, B. P. Lelieveldt, C. Luo, E. A. Mukamel, A. Pinto-Duarte, S. Preissl, A. Regev, B. Ren, R. H. Scheuermann, K. Smith, W. J. Spain, O. R. White, C. Koch, M. Hawrylycz, B. Tasic, E. Z. Macosko, S. A. McCarroll, J. T. Ting, H. Zeng, K. Zhang, G. Feng, J. R. Ecker, S. Linnarsson, E. S. Lein, Comparative cellular analysis of motor cortex in human, marmoset and mouse. *Nature* **598**, 111–119 (2021).

71. A. N. Boettiger, B. Bintu, J. R. Moffitt, S. Y. Wang, B. J. Beliveau, G. Fudenberg, M. Imakaev, L. A. Mirny, C. T. Wu, X. W. Zhuang, Super-resolution imaging reveals distinct chromatin folding for different epigenetic states. *Nature* **529**, 418–422 (2016).

72. X. X. Deng, W. X. Ma, V. Ramani, A. Hill, F. Yang, F. Ay, J. B. Berletch, C. A. Blau, J. Shendure, Z. J. Duan, W. S. Noble, C. M. Disteche, Bipartite structure of the inactive mouse X chromosome. *Genome Biol.* **16**, 152 (2015).

73. L. Giorgetti, B. R. Lajoie, A. C. Carter, M. Attia, Y. Zhan, J. Xu, C. J. Chen, N. Kaplan, H. Y. Chang, E. Heard, J. Dekker, Structural organization of the inactive X chromosome in the mouse. *Nature* **535**, 575–579 (2016).

74. A. Minajigi, J. Froberg, C. Wei, H. Sunwoo, B. Kesner, D. Colognori, D. Lessing, B. Payer, M. Boukhali, W. Haas, J. T. Lee, Chromosomes. A comprehensive Xist

interactome reveals cohesin repulsion and an RNA-directed chromosome conformation. *Science* **349**, aab2276 (2015).

75. M. A. J. Morgan, A. Shilatifard, Reevaluating the roles of histone-modifying enzymes and their associated chromatin modifications in transcriptional regulation. *Nat. Genet.* **52**, 1271–1281 (2020).

76. C. X. Zhu, Y. X. Zhang, Y. E. Li, J. Lucero, M. M. Behrens, B. Ren, Joint profiling of histone modifications and transcriptome in single cells from mouse brain. *Nat. Methods* **18**, 283–292 (2021).

77. H. T. Rube, W. Lee, M. Hejna, H. Y. Chen, D. H. Yasui, J. F. Hess, J. M. LaSalle, J. S. Song, Q. Z. Gong, Sequence features accurately predict genome-wide MeCP2 binding in vivo. *Nat. Commun.* **7**, 11025 (2016).

78. X. S. Nan, F. J. Campoy, A. Bird, MeCP2 is a transcriptional repressor with abundant binding sites in genomic chromatin. *Cell* **88**, 471–481 (1997).

79. M. Chahrour, S. Y. Jung, C. Shaw, X. Zhou, S. T. Wong, J. Qin, H. Y. Zoghbi, MeCP2, a key contributor to neurological disease, activates and represses transcription. *Science* **320**, 1224–1229 (2008).

80. M. J. Lyst, R. Ekiert, D. H. Ebert, C. Merusi, J. Nowak, J. Selfridge, J. Guy, N. R. Kastan, N. D. Robinson, F. de Lima Alves, J. Rappsilber, M. E. Greenberg, A. Bird, Rett syndrome mutations abolish the interaction of MeCP2 with the NCoR/SMRT co-repressor. *Nat. Neurosci.* **16**, 898–902 (2013).

81. Y. Li, H. Y. Wang, J. Muffat, A. W. Cheng, D. A. Orlando, J. Loven, S. M. Kwok, D. A. Feldman, H. S. Bateup, Q. Gao, D. Hockemeyer, M. Mitalipova, C. A. Lewis, M. G. Vander Heiden, M. Sur, R. A. Young, R. Jaenisch, Global transcriptional and translational repression in human-embryonic-stem-cell-derived rett syndrome neurons. *Cell Stem Cell* **13**, 446–458 (2013).

82. H. W. Gabel, B. Kinde, H. Stroud, C. S. Gilbert, D. A. Harmin, N. R. Kastan, M. Hemberg, D. H. Ebert, M. E. Greenberg, Disruption of DNA-methylation-dependent long gene repression in Rett syndrome. *Nature* **522**, 89–93 (2015).

83. W. Renthal, L. D. Boxer, S. Hrvatin, E. Li, A. Silberfeld, M. A. Nagy, E. C. Griffith, T. Vierbuchen, M. E. Greenberg, Characterization of human mosaic Rett syndrome brain tissue by single-nucleus RNA sequencing. *Nat. Neurosci.* **21**, 1670–1679 (2018).

84. B. S. Johnson, Y. T. Zhao, M. Fasolino, J. M. Lamonica, Y. J. Kim, G. Georgakilas, K. H. Wood, D. Bu, Y. Cui, D. Goffin, G. Vahedi, T. H. Kim, Z. L. Zhou, Biotin tagging of MeCP2 in mice reveals contextual insights into the Rett syndrome transcriptome. *Nat. Med.* **23**, 1203–1214 (2017).

85. L. D. Boxer, W. Renthal, A. W. Greben, T. Whitwam, A. Silberfeld, H. Stroud, E. Li, M. G. Yang, B. Kinde, E. C. Griffith, B. Bonev, M. E. Greenberg, MeCP2 represses the rate of transcriptional initiation of highly methylated long genes. *Mol. Cell* **77**, 294–309.e9 (2020).

86. R. Tillotson, J. Cholewa-Waclaw, K. Chhatbar, J. C. Connelly, S. A. Kirschner, S. Webb, M. V. Koerner, J. Selfridge, D. A. Kelly, D. De Sousa, K. Brown, M. J. Lyst, S. Kriaucionis, A. Bird, Neuronal non-CG methylation is an essential target for MeCP2 function. *Mol. Cell* **81**, 1260–1275.e12 (2021).

87. M. J. Sperlazza, S. M. Bilinovich, L. M. Sinanan, F. R. Javier, D. C. Williams, Structural basis of MeCP2 distribution on non-CpG methylated and hydroxymethylated DNA. *J. Mol. Biol.* **429**, 1581–1594 (2017).

88. J. L. Neul, P. Fang, J. Barrish, J. Lane, E. B. Caeg, E. O. Smith, H. Zoghbi, A. Percy, D. G. Glaze, Specific mutations in methyl-CpG-binding protein 2 confer different severity in Rett syndrome. *Neurology* **70**, 1313–1321 (2008).

89. N. A. Quaderi, R. R. Meehan, P. H. Tate, S. H. Cross, A. P. Bird, A. Chatterjee, G. E. Herman, S. D. M. Brown, Genetic and physical mapping of a gene encoding a methyl

Cpg binding-protein, Mecp2 to the mouse X-chromosome. *Genomics* **22**, 648–651 (1994).

90. M. F. Lyon, Gene action in the X-chromosome of the mouse (*Mus musculus* L.). *Nature* **190**, 372–373 (1961).

91. C. Uhler, G. V. Shivashankar, Nuclear mechanopathology and cancer diagnosis. *Trends Cancer* **4**, 320–331 (2018).

92. L. Lu, X. Liu, W. K. Huang, P. Giusti-Rodriguez, J. Cui, S. Zhang, W. Xu, Z. Wen, S. Ma, J. D. Rosen, Z. Xu, C. F. Bartels, R. Kawaguchi, M. Hu, P. C. Scacheri, Z. Rong, Y. Li, P. F. Sullivan, H. Song, G. L. Ming, Y. Li, F. Jin, Robust Hi-C maps of enhancer-promoter interactions reveal the function of non-coding genome in neural development and diseases. *Mol. Cell* **79**, 521–534.e15 (2020).

93. K. Abramo, A. L. Valton, S. V. Venev, H. Ozadam, A. N. Fox, J. Dekker, A chromosome folding intermediate at the condensin-to-cohesin transition during telophase. *Nat. Cell Biol.* **21**, 1393–1402 (2019).

94. T. Nagano, Y. Lubling, C. Vaarnai, C. Dudley, W. Leung, Y. Baran, N. M. Cohen, S. Wingett, P. Fraser, A. Tanay, Cell-cycle dynamics of chromosomal organization at single-cell resolution. *Nature* **547**, 61–67 (2017).

95. S. Leidescher, J. Ribisel, S. Ullrich, Y. Feodorova, E. Hildebrand, A. Galitsyna, S. Bultmann, S. Link, K. Thanisch, C. Mulholland, J. Dekker, H. Leonhardt, L. Mirny, I. Solovei, Spatial organization of transcribed eukaryotic genes. *Nat. Cell Biol.* **24**, 327–339 (2022).

96. S. H. Ahanger, R. N. Delgado, E. Gil, M. A. Cole, J. Zhao, S. J. Hong, A. R. Kriegstein, T. J. Nowakowski, A. A. Pollen, D. A. Lim, Distinct nuclear compartment-associated genome architecture in the developing mammalian brain. *Nat. Neurosci.* **24**, 1235–1242 (2021).

97. T. Isoda, A. J. Moore, Z. He, V. Chandra, M. Aida, M. Denholtz, J. Piet van Hamburg, K. M. Fisch, A. N. Chang, S. P. Fahl, D. L. Wiest, C. Murre, Non-coding transcription instructs chromatin folding and compartmentalization to dictate enhancer-promoter communication and T cell fate. *Cell* **171**, 103–119.e18 (2017).

98. L. Brueckner, P. A. Zhao, T. van Schaik, C. Leemans, J. Sima, D. Peric-Hupkes, D. M. Gilbert, B. van Steensel, Local rewiring of genome-nuclear lamina interactions by transcription. *EMBO J.* **39**, e103159 (2020).

99. T. Nikitina, X. Shi, R. P. Ghosh, R. A. Horowitz-Scherer, J. C. Hansen, C. L. Woodcock, Multiple modes of interaction between the methylated DNA binding protein MeCP2 and chromatin. *Mol. Cell. Biol.* **27**, 864–877 (2007).

100. G. N. L. Chua, J. W. Watters, P. D. B. Olinares, M. Begum, L. E. Vostal, J. A. Luo, B. T. Chait, S. X. Liu, Differential dynamics specify MeCP2 function at nucleosomes and methylated DNA. *Nat. Struct. Mol. Biol.* **31**, 1789–1797 (2024).

101. P. T. Georgel, R. A. Horowitz-Scherer, N. Adkins, C. L. Woodcock, P. A. Wade, J. C. Hansen, Chromatin compaction by human MeCP2 - Assembly of novel secondary chromatin structures in the absence of DNA methylation. *J. Biol. Chem.* **278**, 32181–32188 (2003).

102. J. Y. Sonn, W. Kim, M. Iwanaszko, Y. Aoi, Y. Li, L. Parkitny, J. L. Brissette, L. Weiner, I. Al-Ramahi, J. Botas, A. Shilatifard, H. Y. Zoghbi, MeCP2 interacts with the super elongation complex to regulate transcription. *bioRxiv*, 2024.06.30.601446 (2024).

103. W. K. Cho, J. H. Spille, M. Hecht, C. Lee, C. Li, V. Grube, I. I. Cisse, Mediator and RNA polymerase II clusters associate in transcription-dependent condensates. *Science* **361**, 412–415 (2018).

104. M. Boehning, C. Dugast-Darzacq, M. Rankovic, A. S. Hansen, T. Yu, H. Marie-Nelly, D. T. McSwiggen, G. Kokic, G. M. Dailey, P. Cramer, X. Darzacq, M. Zweckstetter, RNA

polymerase II clustering through carboxy-terminal domain phase separation. *Nat. Struct. Mol. Biol.* **25**, 833–840 (2018).

105. Y. E. Guo, J. C. Manteiga, J. E. Henninger, B. R. Sabari, A. Dall'Agnese, N. M. Hannett, J. H. Spille, L. K. Afeyan, A. V. Zamudio, K. Shrinivas, B. J. Abraham, A. Boija, T. M. Decker, J. K. Rimel, C. B. Fant, T. I. Lee, I. I. Cisse, P. A. Sharp, D. J. Taatjes, R. A. Young, Pol II phosphorylation regulates a switch between transcriptional and splicing condensates. *Nature* **572**, 543–548 (2019).

106. R. Pantier, M. Brown, S. Han, K. Paton, S. Meek, T. Montavon, N. Shukeir, T. McHugh, D. A. Kelly, T. Hochepied, C. Libert, T. Jenuwein, T. Burdon, A. Bird, MeCP2 binds to methylated DNA independently of phase separation and heterochromatin organisation. *Nat. Commun.* **15**, 3880 (2024).

107. T. Lu, C. E. Ang, X. W. Zhuang, Spatially resolved epigenomic profiling of single cells in complex tissues. *Cell* **185**, 4448–4464.e17 (2022).

108. Y. Zhang, L. Boninsegna, M. Y. Yang, T. Misteli, F. Alber, J. Ma, Computational methods for analysing multiscale 3D genome organization. *Nat. Rev. Genet.* **25**, 123–141 (2024).

109. B. Chen, L. A. Gilbert, B. A. Cimini, J. Schnitzbauer, W. Zhang, G. W. Li, J. Park, E. H. Blackburn, J. S. Weissman, L. S. Qi, B. Huang, Dynamic imaging of genomic loci in living human cells by an optimized CRISPR/Cas system. *Cell* **155**, 1479–1491 (2013).

110. H. Wang, M. Han, L. S. Qi, Engineering 3D genome organization. *Nat. Rev. Genet.* **22**, 343–360 (2021).

111. J. Guy, B. Hendrich, M. Holmes, J. E. Martin, A. Bird, A mouse Mecp2-null mutation causes neurological symptoms that mimic Rett syndrome. *Nat. Genet.* **27**, 322–326 (2001).

112. Q. Wang, S. L. Ding, Y. Li, J. Royall, D. Feng, P. Lesnar, N. Graddis, M. Naeemi, B. Facer, A. Ho, T. Dolbeare, B. Blanchard, N. Dee, W. Wakeman, K. E. Hirokawa, A.

Szafer, S. M. Sunkin, S. W. Oh, A. Bernard, J. W. Phillips, M. Hawrylycz, C. Koch, H. Zeng, J. A. Harris, L. Ng, The allen mouse brain common coordinate framework: A 3D reference atlas. *Cell* **181**, 936–953.e20 (2020).

113. Y. Zhang, T. Liu, C. A. Meyer, J. Eeckhoute, D. S. Johnson, B. E. Bernstein, C. Nusbaum, R. M. Myers, M. Brown, W. Li, X. S. Liu, Model-based analysis of ChIP-seq (MACS). *Genome Biol.* **9**, R137 (2008).

114. J. Loven, H. A. Hoke, C. Y. Lin, A. Lau, D. A. Orlando, C. R. Vakoc, J. E. Bradner, T. I. Lee, R. A. Young, Selective inhibition of tumor oncogenes by disruption of super-enhancers. *Cell* **153**, 320–334 (2013).

115. Q. K. Xu, M. R. Schlabach, G. J. Hannon, S. J. Elledge, Design of 240,000 orthogonal 25mer DNA barcode probes. *Proc. Natl. Acad. Sci. U.S.A.* **106**, 2289–2294 (2009).

116. C. Camacho, G. Coulouris, V. Avagyan, N. Ma, J. Papadopoulos, K. Bealer, T. L. Madden, BLAST plus: Architecture and applications. *BMC Bioinformatics* **10**, 421 (2009).

117. G. Emanuel, seichhorn, H. Babcock, leonardosepulveda, timblosser, ZhuangLab/MERlin: MERlin v0.1.6 (Zenodo 2020); doi:10.5281/zenodo.3758540.

118. J. Kennedy-Darling, L. M. Smith, Measuring the formaldehyde protein-DNA cross-link reversal rate. *Anal. Chem.* **86**, 5678–5681 (2014).

119. M. Kozubek, P. Matula, An efficient algorithm for measurement and correction of chromatic aberrations in fluorescence microscopy. *J. Microsc.-Oxford* **200**, 206–217 (2000).

120. M. Pachitariu, C. Stringer, Cellpose 2.0: How to train your own model. *Nat. Methods* **19**, 1634–1641 (2022).

121. F. A. Wolf, P. Angerer, F. J. Theis, SCANPY: Large-scale single-cell gene expression data analysis. *Genome Biol.* **19**, 15 (2018).

122. S. L. Wolock, R. Lopez, A. M. Klein, Scrublet: Computational identification of cell doublets in single-cell transcriptomic data. *Cell Syst.* **8**, 281–291.e9 (2019).

123. Z. Z. Yao, C. T. J. van Velthoven, M. Kunst, M. Zhang, D. Mcmillen, C. K. Y. Lee, W. Jung, J. Goldy, A. Abdelhak, M. Aitken, K. Baker, P. Baker, E. Barkan, D. Bertagnolli, A. Bhandiwad, C. Bielstein, P. Bishwakarma, J. Campos, D. Carey, T. Casper, A. B. Chakka, R. Chakrabarty, S. Chavan, M. Chen, M. Clark, J. Close, K. Crichton, S. Daniel, P. Divalentin, T. Dolbeare, L. Ellingwood, E. Fiabane, T. Fliss, J. Gee, J. Gerstenberger, A. Glandon, J. Gloe, J. Gould, J. Gray, N. Guilford, J. Guzman, D. Hirschstein, W. Y. Ho, M. Hooper, M. K. Huang, M. Hupp, K. L. Y. Jin, M. Kroll, K. Lathia, A. Leon, S. Li, B. Long, Z. Madigan, J. Malloy, J. Malone, Z. Maltzer, N. Martin, R. Mccue, R. Mcginty, N. C. L. Mei, J. Melchor, E. Meyerdierks, T. Mollenkopf, S. Moonsman, T. N. Nguyen, S. Otto, T. Pham, C. Rimorin, A. Ruiz, R. Sanchez, L. Sawyer, N. Shapovalova, N. Shepard, C. Slaughterbeck, J. Sulc, M. Tieu, A. Torkelson, H. R. Tung, N. V. Cuevas, S. Vance, K. Wadhwani, K. Ward, B. Levi, C. Farrell, R. Young, B. Staats, M. Q. M. Wang, C. L. Thompson, S. Mufti, C. M. Pagan, L. Kruse, N. Dee, S. M. Sunkin, L. Esposito, M. J. Hawrylycz, J. Waters, L. Ng, K. Smith, B. Tasic, X. W. Zhuang, H. K. Zeng, A high-resolution transcriptomic and spatial atlas of cell types in the whole mouse brain. *Nature* **624**, 317–332 (2023).

124. Y. Zhang, S. A. Sloan, L. E. Clarke, C. Caneda, C. A. Plaza, P. D. Blumenthal, H. Vogel, G. K. Steinberg, M. S. Edwards, G. Li, J. A. Duncan, 3rd, S. H. Cheshier, L. M. Shuer, E. F. Chang, G. A. Grant, M. G. Gephart, B. A. Barres, Purification and characterization of progenitor and mature human astrocytes reveals transcriptional and functional differences with mouse. *Neuron* **89**, 37–53 (2016).

125. E. Crane, Q. Bian, R. P. McCord, B. R. Lajoie, B. S. Wheeler, E. J. Ralston, S. Uzawa, J. Dekker, B. J. Meyer, Condensin-driven remodelling of X chromosome topology during dosage compensation. *Nature* **523**, 240–244 (2015).

126. U. Raudvere, L. Kolberg, I. Kuzmin, T. Arak, P. Adler, H. Peterson, J. Vilo, g:Profiler: A web server for functional enrichment analysis and conversions of gene lists (2019 update). *Nucleic Acids Res.* **47**, W191–W198 (2019).

127. M. I. Love, W. Huber, S. Anders, Moderated estimation of fold change and dispersion for RNA-seq data with DESeq2. *Genome Biol.* **15**, 550 (2014).

128. Y. S. Zhang, C. Pak, Y. Han, H. Ahlenius, Z. J. Zhang, S. Chanda, S. Marro, C. Patzke, C. Acuna, J. Covy, W. Xu, N. Yang, T. Danko, L. Chen, M. Wernig, T. C. Sudhof, Rapid single-step induction of functional neurons from human pluripotent stem cells. *Neuron* **78**, 785–798 (2013).

129. P. J. Skene, R. S. Illingworth, S. Webb, A. R. W. Kerr, K. D. James, D. J. Turner, R. Andrews, A. P. Bird, Neuronal MeCP2 is expressed at near histone-octamer levels and globally alters the chromatin state. *Mol. Cell* **37**, 457–468 (2010).
